# A new acidic microenvironment related lncRNA signature predicts the prognosis of liver cancer patients

**DOI:** 10.3389/fonc.2022.1016721

**Published:** 2022-10-31

**Authors:** Peng Jiang, Wenbo Xue, Cheng Xi, Lin Zhuang, Zhiping Yuan, Zhilin Liu, Tao Sun, Xuezhong Xu, Yulin Tan, Wei Ding

**Affiliations:** ^1^ Department of General Surgery, Wujin Hospital Affiliated with Jiangsu University, Changzhou, China; ^2^ Department of General Surgery, The Wujin Clinical College of Xuzhou Medical University, Changzhou, China; ^3^ Department of Gastroenterology, Wujin Hospital Affiliated with Jiangsu University, Changzhou, China; ^4^ Department of Gastrointestinal Surgery, The Third Affiliated Hospital of Soochow University, Changzhou, China; ^5^ Department of Hepatopancreatobiliary Surgery, The Third Affiliated Hospital of Soochow University, Changzhou, China; ^6^ Changzhou Key Laboratory of Molecular Diagnostics and Precision Cancer Medicine, Wujin Hospital Affiliated with Jiangsu University, Changzhou, China

**Keywords:** acidic microenvironment, lncRNAs, liver cancer, immune infiltration, drug therapy

## Abstract

**Background:**

The acidic microenvironment (AME), like hypoxia, inflammation, or immunoreaction, is a hallmark of the tumor microenvironment (TME). This work aimed to develop a prediction signature dependent on AME-associated lncRNAs in order to predict the prognosis of LC individuals.

**Methods:**

We downloaded RNA-seq information and the corresponding clinical and predictive data from The Cancer Genome Atlas (TCGA) dataset and conducted univariate and multivariate Cox regression analyses to identify AME-associated lncRNAs for the construction of a prediction signature The Kaplan-Meier technique was utilized to determine the overall survival (OS) rate of the high (H)-risk and low (L)-risk groups. Using gene set enrichment analysis (GSEA) the functional variations between the H- and L-risk groups were investigated. The association between the prediction signature and immunological state was investigated using single-sample GSEA (ssGSEA). Additionally, the association between the predicted signature and the therapeutic response of LC individuals was evaluated. Lastly, quantitative reverse transcription polymerase chain reaction (qRT-PCR) was performed to verify the risk model.

**Results:**

We generated a signature comprised of seven AME-associated lncRNAs (LINC01116, AC002511.2, LINC00426, ARHGAP31-AS1, LINC01060, TMCC1-AS1, AC012065.1). The H-risk group had a worse prognosis than the L- risk group. The AME-associated lncRNA signature might determine the prognosis of individuals with LC independently. The AME-related lncRNA signature shows a greater predictive effectiveness than clinic-pathological factors, with an area under the receiver operating characteristic (ROC) curve of 0.806%. When participants were categorized based on several clinico-pathological characteristics, the OS of high-risk individuals was shorter compared to low-risk patients. GSEA demonstrated that the metabolism of different acids and the PPAR signaling pathway are closely associated with low-risk individuals. The prognostic signature was substantially associated with the immunological status of LC individuals, as determined by ssGSEA. High risk individuals were more sensitive to some immunotherapies (including anti-TNFSF4 anti-SIRPA, anti-CD276 and anti-TNFSF15) and some conventional chemotherapy drugs (including lapatinib and paclitaxel). Finally, the expression levels of the seven lncRNAs comprising the signature were tested by qRT-PCR.

**Conclusions:**

A basis for the mechanism of AME-associated lncRNAs in LC is provided by the prediction signature, which also offers clinical therapeutic recommendations for LC individuals.

## Introduction

Globally, liver cancer (LC) accounts for the 4^th^ most prevalent tumor-related death and ranks sixth in terms of cancer morbidity, the incidence of which is on the rise (1, 2). Surgery is currently the most popular type of therapy for LC, although most individuals are already in advanced stages when they’re found, and some patients with severe cirrhosis are not suitable candidates for surgical treatment (3). Despite advances in curative LC treatment in recent years, the five-year survival rate (SR) for LC individuals is still low owing to the disease’s spread, metastasis, and recurrence rate (4). Therefore, understanding the molecular mechanisms underlying the progression and searching for diagnostic indicators of LC can be crucial for detecting recurrences of LC and identifying novel treatment strategies.

Because tumor cells are typically reprogrammed for glycolysis, which generates lactic acid (LA) even under aerobic conditions, and tumor vasculature is typically dysfunctional, the tumor microenvironment (TME) has an acidic pH, sometimes known as acidic microenvironment (AME) (5). The acidic microenvironment, like hypoxia, inflammation, or immunoreaction, is a hallmark of the TME (6–8). Recent research has demonstrated that AME can promote numerous crucial oncogenic pathways, such as angiogenesis, tissue invasion/metastasis, and medication resistance (9). By promoting autophagy in an acidic environment, FOXO3a prevented the development of human gastric adenocarcinoma cell (10). Acidic microenvironment can increase hepatocellular carcinoma (HCC) cell-derived exosomal miR-21 and miR-10b levels, hence stimulating HCC cell motility and invasion (11).

Long noncoding RNA (lncRNA) was typically described as RNA with a limited ability to for code protein, which was strongly associated with the inactivation of cancer suppressor genes and the activation of oncogenes in HCC (12). lncRNA-PDPK2P increases the progression of HCC *via* the PDK1/AKT/Caspase 3 pathway (13). BCAR4 increases LC development by elevating ANAPC11 expression *via* miR1261 sponging (14). LncRNA NBR2 suppresses carcinogenesis in HCC through controlling autophagy (15). There are currently fewer investigations on lncRNAs associated to acidic microenvironment, and no study has been published on AME-associated lncRNAs in LC. In this investigation, a prediction signature based on AME-associated lncRNAs was constructed and assessed for prognosis, chemotherapeutic response, and tumor immune infiltration in LC individuals. Internal validation was also performed. As a next step, we conducted gene enrichment analysis (GSEA) to investigate possible mechanisms.

## Methods

### Patients and tissue samples

We gathered gene expression RNAseq and accompanying medical and predictive data for The Cancer Genome Atlas liver cancer (TCGA-LIHC) database from the UCSC Xena website (https://xenabrowser.net/datapages/); data for 424 samples were obtained. A total of 788 AME-related genes (relevance score > 7) were downloaded from GeneCards website (https://www.genecards.org/). 345 patients who met the eligibility requirements of being followed for more than 30 days were taken part in the investigation. All participants were assigned in a random fashion into two groups: training (n = 173) and testing (n = 172), with a ratio of 1:1. The demographic characteristics of patients in the two groups are shown in **Table 1**.

**Table 1:** The clinical characteristics of patients in different groups.

Variables	Training group(n=173)	Testing group (n=172)	Combined group(n=345)	*P* value(Training vs. Testing)
Age				
≤65	115	104	219	0.247
>65	58	68	126	
Gender				
Male	122	114	236	0.397
Female	51	58	109	
Grade				
I-II	106	109	215	0.856
III-IV	64	61	125	
Unknow	3	2	5	
T stage				
T1-T2	128	127	255	0.841
T3-T4	44	43	87	
TX-Unknow	1	2	3	
N Stage				
N0	122	119	241	0.828
N1	1	2	3	
NX-Unknow	50	51	101	
M stage				
M0	124	122	246	0.217
M1	0	3	3	
MX	49	47	96	
Stage				
I-II	124	117	241	0.125
III-IV	43	40	83	
Unknow	6	15	21	

T, tumor; M, metastasis; N, lymph node.

From January 2021 to August 2021, 10 human HCC samples were collected at Wujin Hospital Affiliated with Jiangsu University (Changzhou, China). Neither chemotherapy nor radiotherapy had been administered to any of these patients before surgery. The study was approved by the Wujin Hospital Institutional Ethical Review Board (2022-SR-086), and informed consent was obtained from each patient.

### Functional enrichment analysis (FEA) of differentially expressed AME-associated genes

As screening criteria for identifying differentially expressed genes (DEGs) related with AME, we applied a false discovery rate< 0.05 and log_2_|fold change (FC)| > 1. The “clusterProfiler” software (Ver. 4.4.4) was employed to assess Gene Ontology (GO) and Kyoto Encyclopedia of Genes and Genomes (KEGG) evaluations.

### Construction of the prognostic signature for AME-associated lncRNA

Using the “limma” program (Ver. 3.52.2), the association between AME-associated genes and lncRNAs was computed. On the basis of a correlation coefficient r^2^ > 0.5 and *P*< 0.001, 542 AME-associated lncRNAs with expression values were identified. Firstly, we employed univariate Cox regression (UCR) assessment to identify AME-associated lncRNAs correlated to the prognosis of LC individuals., Next, we used least absolute shrinkage and selection operator (LASSO) regression assessment to exclude high-impact factors, Eventually, the risk score model was developed using multivariate Cox regression (MCR)evaluation. The risk score equation was built as the following:


$$Risk\;score=\sum_{i=0}^{n}\beta_{i}*Exp_{i}$$

In this equation, *β* is the regression coefficient acquired from the multivariate Cox regression analysis and *Exp* is the expression value of selected AME-associated lncRNAs. Each LC patient received a risk score according to this equation.

### Construction of nomogram for overall survival (OS)

On the basis of the risk score and clinicopathological parameters of age, gender, tumor stage (T, N and M), we created a nomogram that predicts one-, three-, and five-year survival in LC individuals. We utilized a calibration curve to determine if the expected SR was in line accordance with the actual SR.

### FEA of the AME-associated lncRNA prognostic signature

LC individuals were categorized into high (H)- and low (L)-risk groups on the basis of the median value of their risk scores. Gene enrichment analysis with GSEA was employed to determine which pathways were enriched the most (16). The analysis was conducted using GSEA 4.2.3 (http://www.gsea-msigdb.org/gsea/). We considered nominal *p*< 0.05 and FDR<0.25 to be statistically significance.

### Immune infiltration and immune checkpoint analysis of AME-related lncRNA predictive signature

Utilizing “GSVA” package (Version 1.44.2) by single-sample gene set enrichment analysis (ssGSEA), the infiltration scores of 28 immune cells were computed (17). The phenotypic genes of immune cells were downloaded from the TISIDB website (http://cis.hku.hk/TISIDB/). A total of 40 immune checkpoints (**Supplementary Table S1**) were assessed with the Wilcoxon signed-rank test (WS-RT).

### The function of the prognostic signature in predicting medical therapeutic response

The half-maximal inhibitory concentrations (IC50) of well-known chemotherapy agents were computed to evaluate whether the predictive signature predicts the outcome of LC therapy. The WS-RT was performed to evaluate the IC50 values across the H- and L-risk groups.

### RNA extraction and quantitative RT-PCR

Total RNA was extracted from the liver tissues using TRIzol™ (Invitrogen, Carlsbad, CA, USA), and 2.0 μg of total RNA were applied to reverse transcription using the PrimeScript™ RT reagent Kit with gDNA Eraser (TaKaRa, Tokyo, Japan). Quantitative PCR was conducted using the TB Green^®^ Premix Ex Taq™ II (TaKaRa, Tokyo, Japan). LncRNAs expression were quantified using the 2^−ΔΔCt^ method and standardized to GAPDH. The primer sequences are listed in **Supplementary Table S2**.

### Statistical analysis

All statistical evaluations were conducted using R (ver 4.2.0) and GraphPad Prism (ver 9.0.1), and all statistical tests were two-tailed, with a *p* value< 0.05 deemed statistical significance. The Kaplan-Meier (K-M) technique and log-rank test were utilized to evaluate the OS of individuals in the H- and L-risk groups. The “timeROC” (ver. 0.4) program was utilized to plot the ROC curves and compute the area under the curve (AUC) results. Principal component analysis (PCA) was employed to evaluate the distribution of participant with various risk scores.

## Results

### Enrichment analysis of AME-associated genes

**Figure 1:**
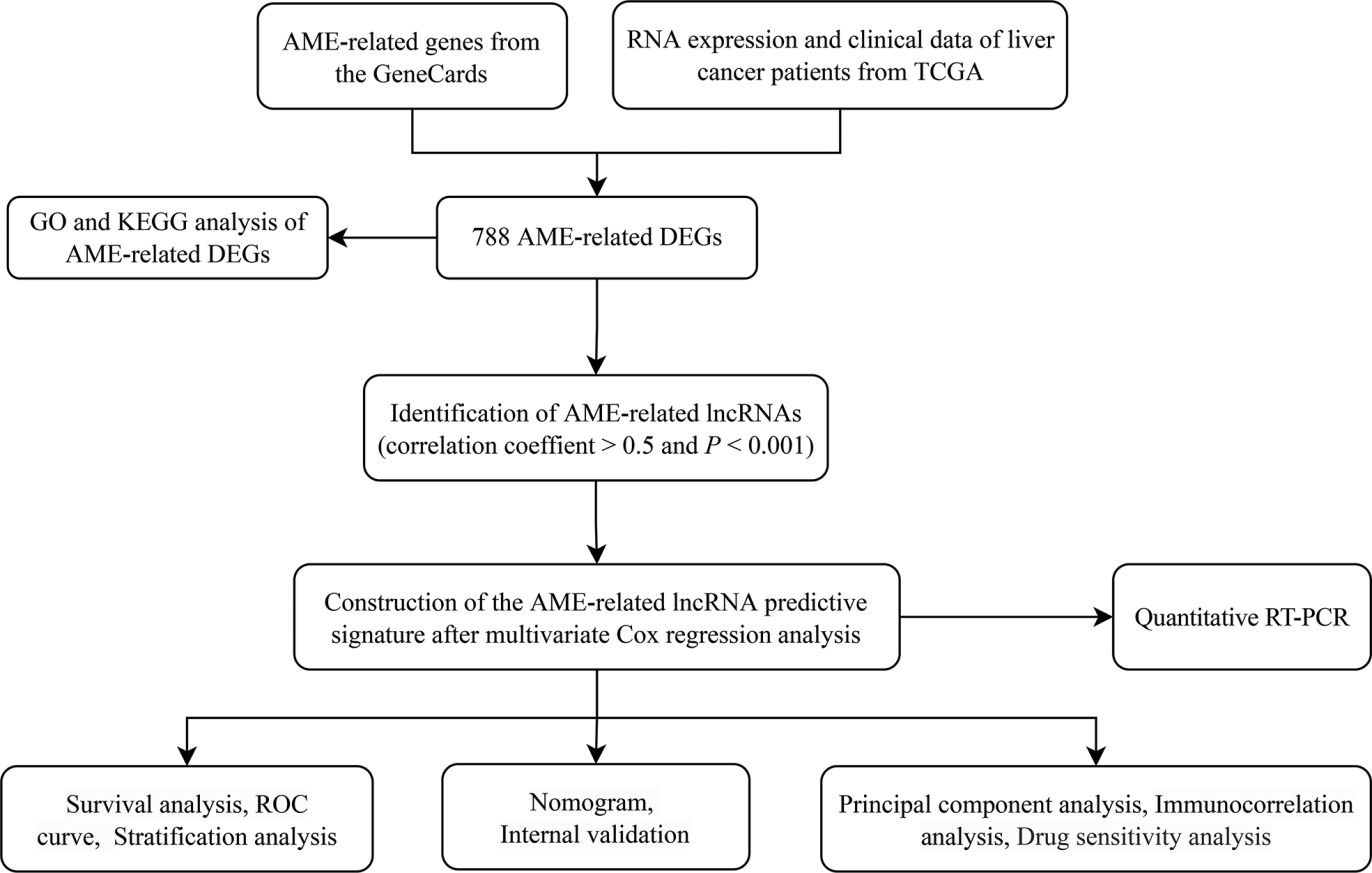
The flowchart of our research. TCGA, The Cancer Genome Atlas; DFS, disease-free survival; DEGs, differentially expressed genes; GO, Gene Ontology; KEGG, Kyoto Encyclopedia of Genes and Genomes; lncRNAs, long noncoding RNAs.

**Figure 2:**
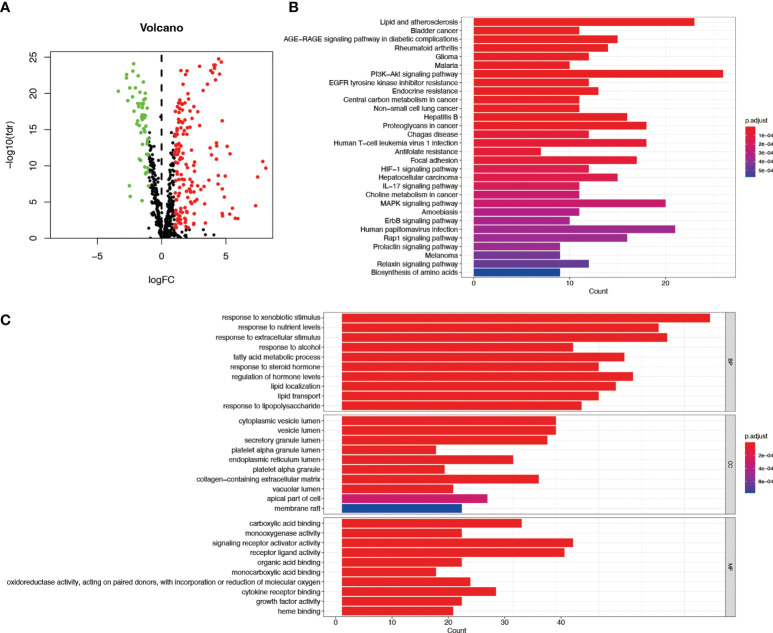
GO and KEGG assessments of AME-associated DEGs in tumor and adjacent tissues. **(A)** Volcano plot of 788 AME-associated genes in liver cancer. Red dots show up-regulated genes, while green dots indicate down-regulated genes. **(B)** KEGG assessment of AME-associated DEGs. **(C)** GO evaluation of AME-associated DEGs. FC, fold change; fdr, false discovery rate; BP, biological process; CC, cellular components; MF, molecular function.

**Figure 1** depicts the pipeline of the present investigation. We obtained 222 AME-associated DEGs (**Figure 2A**). KEGG and GO analyses were performed on DEGs associated with AME. KEGG pathway evaluation demonstrated that AME- associated DEGs were predominantly enriched in lipid and atherosclerosis, bladder cancer, AGE−RAGE signaling pathway in diabetes - related complications, rheumatoid arthritis, glioma, PI3K-Akt signaling pathway, central carbon metabolism in tumor, non-small cell lung cancer, etc. (**Figure 2B**). In the domain of biological process, GO findings revealed that DEGs were predominantly enriched in reaction to xenobiotic stimulation, response to nutritional concentrations, response to extracellular stimuli, etc. DEGs were predominantly abundant in cytoplasmic vesicle lumen, secretory granule lumen, platelet alpha granule lumen, and other lumens of cellular organelles. In the area of molecular activity, the DEGs were primarily enriched for carboxylic acid binding, monooxygenase activity, signaling receptor activator activity, etc. (**Figure 2C**).

### Construction of prognostic signature for the AME-associated lncRNA

**Figure 3:**
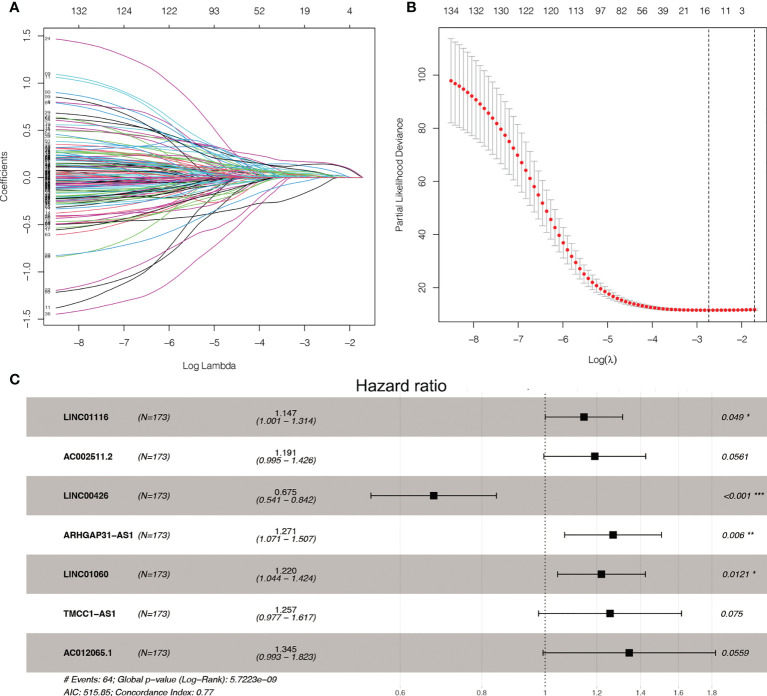
LASSO and Cox regression for tuning parameter selection. **(A)** LASSO coefficient profiles of the 136 AME-related lncRNAs. **(B)** Plots of the ten-fold cross-validation error rates. **(C)** Forest map of the seven prognostic lncRNAs by multivariate Cox regression.

**Figure 4:**
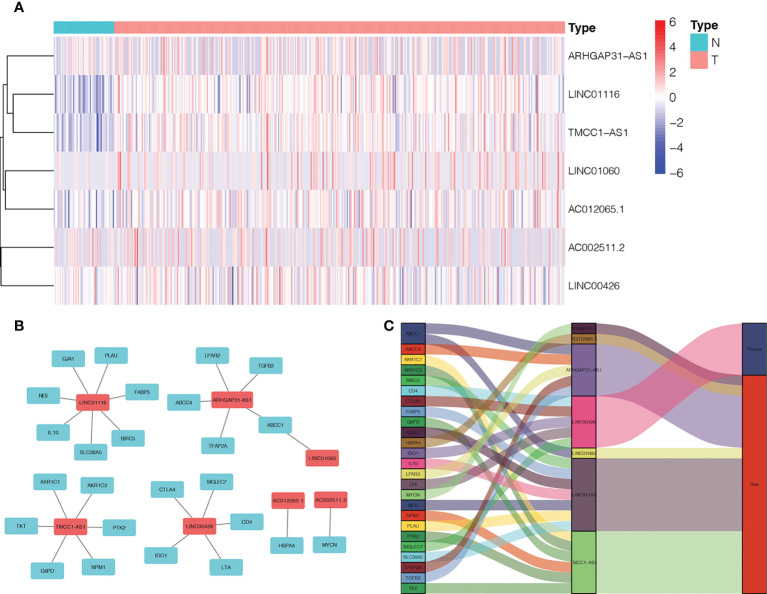
Expression levels and lncRNA-mRNA network of seven AME-associated lncRNAs in the predicted signature. **(A)** The expression levels of seven AME-related lncRNAs in liver cancer and normal tissues. **(B)** The co-expression network of prognostic AME-associated lncRNAs. **(C)** Sankey diagram of prognostic AME-based lncRNAs. T, tumor; N, non-tumor.

We found 542 AME-associated lncRNAs (**Supplementary Table S3**). UCR analysis demonstrated that 136 lncRNAs were correlated with the prognosis of LC individuals (**Supplementary Table S4**). LASSO analysis screened out 16 high-impact AME-related lncRNAs (**Figures 3A, B**). MCR analysis showed that a seven AME-related lncRNAs (LINC01116, AC002511.2, LINC00426, ARHGAP31-AS1, LINC01060, TMCC1-AS1, AC012065.1) prognostic signature (ALPS) was identified (**Figure 3C**). **Figure 4A** depicts the levels of expression of seven AME-associated lncRNAs in LC individuals. On the basis of Pearson’s correlation (r^2^ > 0.5 and *P*< 0.001), the lncRNA-mRNA co-expression network was created. **Figures 4B, C** illustrates additional visualizations of the network using Cytoscape and the “ggalluvial” module. ARHGAP31-AS1 was co-expressed with five AME-associated genes (LPAR2, ABCC1, ABCC4, TGFB2 and TFAP2), LINC00426 was co-expressed with five AME-related genes (IDO1, SIGLEC7, CD4, CTLA4 and LTA), LINC01116 was co-expressed with seven AME-associated genes (FABP5, PLAU, IL10, BIRC5, SLC38A5, GJA1 and NES), TMCC1-AS1 was co-expressed with six AME-related genes (G6PD, AKR1C1, PTK2, NPM1, TKT and AKR1). AC002511.2 was co-expressed with MYCN, AC012065.1 was co-expressed with HSPA4, LINC01060 was co-expressed with ABCC1. The score for risk was computed as follows:


$$\text{Risk score}=\left(0.137*{\text{EXP}}_{\text{LINC}01116}\right)+\left(0.175*{\text{EXP}}_{\text{AC}002511.2}\right)+\left(-0.393*{\text{EXP}}_{\text{LINC}00426}\right)+\left(0.239*{\text{EXP}}_{\text{ARHGAP}31-\text{AS}1}\right)+\left(0.198*{\text{EXP}}_{\text{LINC}01060}\right)+\left(0.229*{\text{EXP}}_{\text{TMCC}1-\text{AS}1}\right)+\left(0.297*{\text{EXP}}_{\text{AC}012065.1}\right)$$

### Association between the ALPS, clinic-pathological features and the prognosis of LC patients

**Figure 5:**
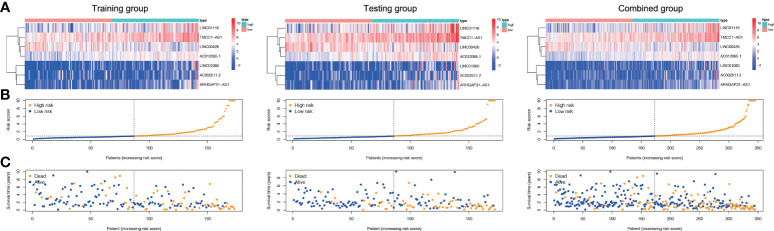
Risk score of the three groups. **(A)** Heatmap of the three-gene signature in the training, testing, and combined groups. **(B)** Allocation of individuals with various risk scores in the training, testing, and combined groups. **(C)** Survival status of individuals with various risk scores in the training, testing, and combined groups.

**Figure 6:**
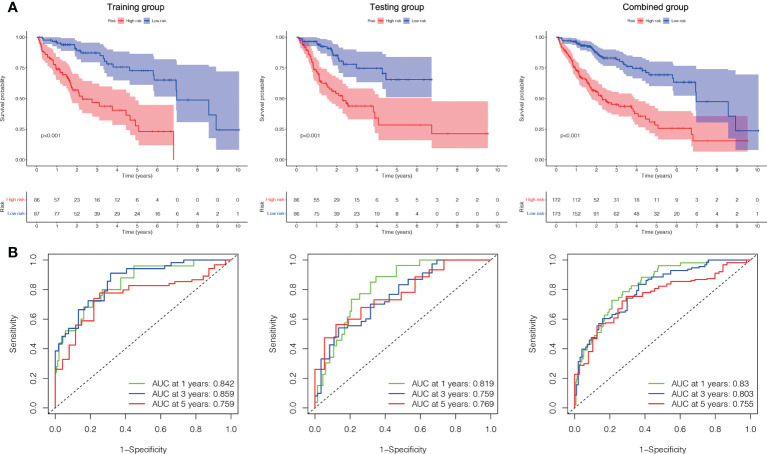
Internal validation of the ALPS for OS according to the entire TCGA database. **(A)** Kaplan-Meier survival curve in the training, testing and combined groups. **(B)** ROC curve and AUCs at one-year, three-years and five-years survival in the training, testing and combined groups. ALPS, AME-associated lncRNAs prognostic signature.

Based on their median risk ratings (0.8587, determined in the training group), the individuals were categorized into 2 groups (H-risk and L-risk) (**Figure 5**). **Figure 6A** demonstrates that in the training, testing and combined groups, individuals in the H-risk group showed poorer prognoses, as shown in **Figure 6A**. Additionally the accuracy of the ALPS in predicting the prognosis of LC in all groups was assessed using ROC curve analysis (**Figure 6B**). Our outcomes showed that the ALPS could be a reliable marker of LC prognosis.

**Figure 7:**
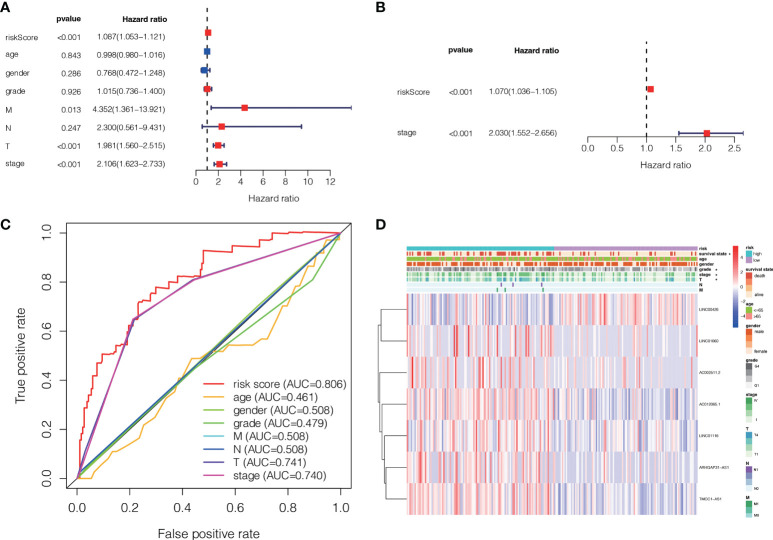
The association between the ALPS and the prognosis of liver individuals. **(A)** Forest plot for univariate Cox regression assessment. **(B)** Forest plot for multivariate Cox regression analysis. **(C)** The ROC curve of the risk score and clinio-pathological factors. **(D)** Heatmap of the ALPS and clinical significance in the combined group. T, tumor; N, non-tumor.

A Cox regression analysis was undertaken to evaluate if the predictive signature is an independent prognostic variable for LC individuals. Both T and M stage, as well as risk score, were substantially related to the OS of LC individuals, according to UCR analysis. (**Figure 7A**). Analysis using MCR revealed that stage and risk score were independent predictors of OS in individuals with LC (**Figure 7B**). In terms of estimating the prognosis of LC individuals, the AUC of the risk score was 0.806%, that was superior to those of clinicopathological factors (**Figure 7C**). We evaluated the variation in clinicopathological factors between the H- and L- risk groups and reported that tumor stage (*P*< 0.05), T stage (*P*< 0.05), grade (*P*< 0.01) and state (*P*< 0.05) were substantially different (**Figure 7D**).

**Figure 8:**
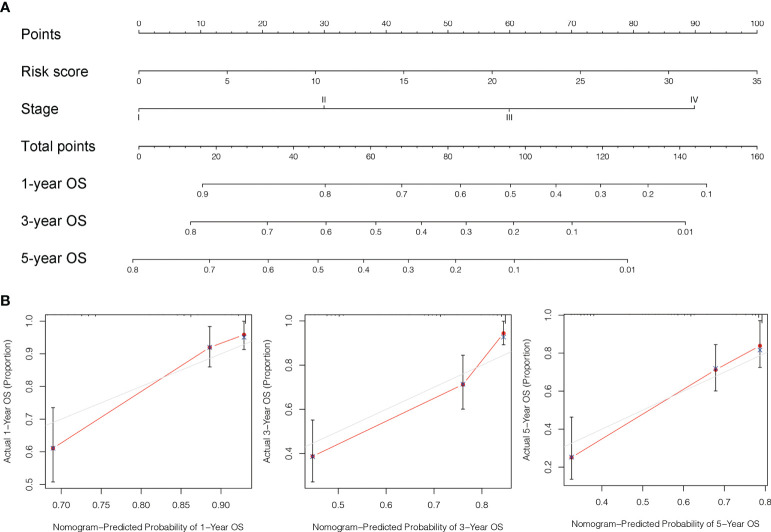
Design and validation of the nomogram. **(A)** A nomogram integrating combining clinio-pathological factors and risk score predicts one, three, and five- years OS of liver cancer individuals. **(B)** The calibration curves assess consistency among the actual OS rates and the expected survival rates at one-, three- and five-years.

To further estimate the one, three, and five-years prognosis of LC individuals, we developed a nomogram using stage and the risk score (**Figure 8A**). The calibration curves demonstrated good consistency among actual OS rates and expected survival rates at one-, three- and five-years (**Figures 8B**).

**Figure 9:**
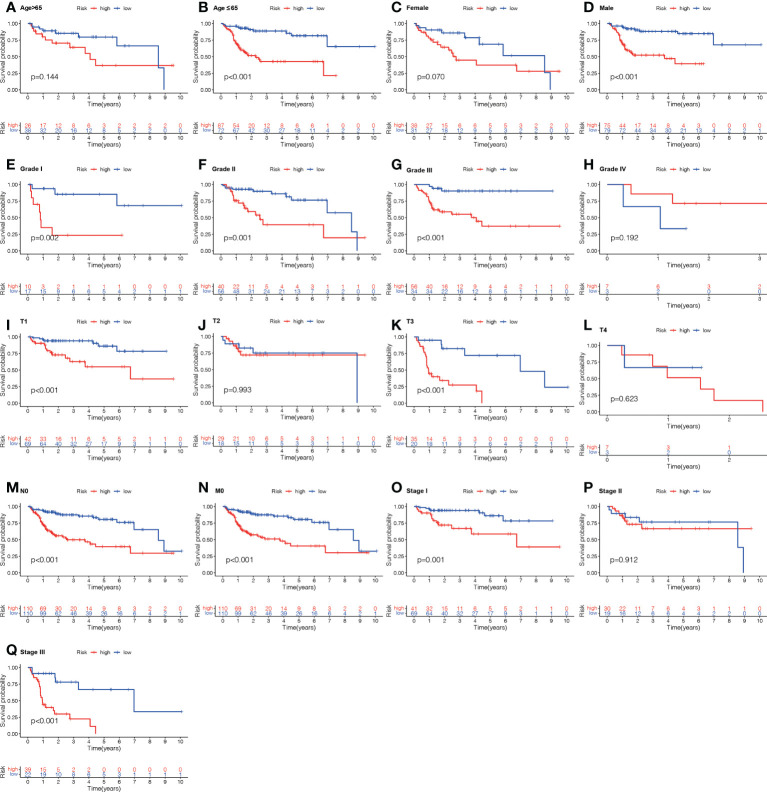
Kaplan-Meier survival graphs for high (H)- and low (L)-risk individual subgroups based on various clinic-pathological factors. **(A, B)** Age. **(C, D)** Gender. **(E–H)** Grade. **(I–L)** T stage. **(M)** N stage. **(N)** M stage. **(O–Q)** Stage. T, tumor; N, Node; M, metastasis.

To examine the correlation between the ALPS and the prognosis of LC individuals categorized by various clinicopathological factors, LC individuals were allocated into groups based on age, sex, and stage (T, N, and M). The OS of individuals in the H- risk group was considerably shorter relative to those of individuals in the L- risk group across all classes (**Figure 9**). The findings demonstrated that the ALPS could predict the outcome of individuals with LC regardless of their clinicopathological characteristics.

### Immune cell infiltration (ICI) and immune checkpoint

**Figure 10:**
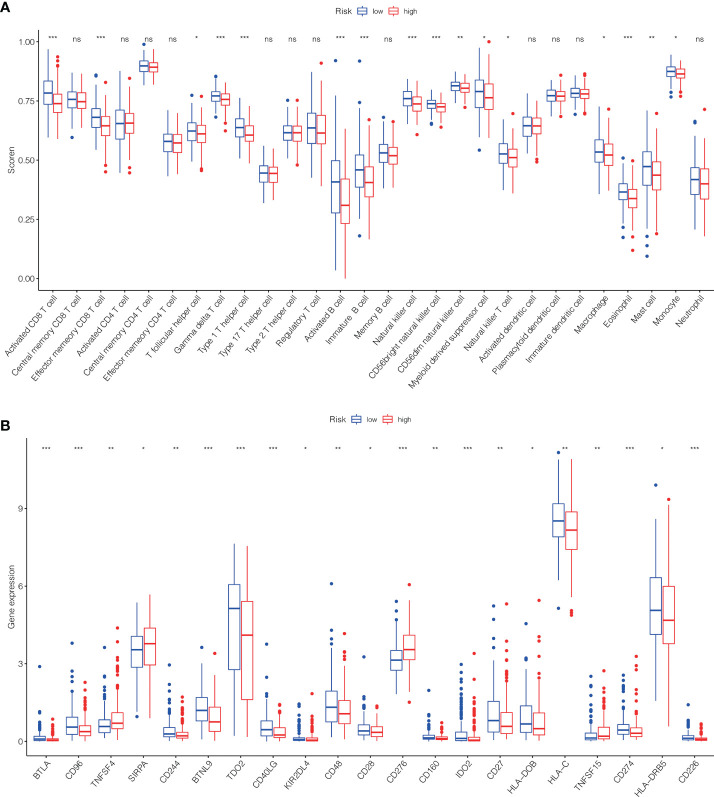
The scores of immune infiltrating cells (IIC) and immunological checkpoint in H- and L- risk groups. **(A)** The infiltration levels of 28 immune cells in H- and L- risk groups. **(B)** The expression levels of 21 immune checkpoint in H- and L- risk groups. *P < 0.05; **P < 0.01; ***P < 0.001; ns, no significant.

To further investigate the association among risk scores and immune cells, we assessed the enrichment scores of ssGSEA for several subgroups of immune cells. The results showed that activated CD8 T cell, effector memeory CD8 T cell, T follicular helper cell, gamma delta T cell, type 1 T helper cell, activated B cell, immature B cell, natural killer cell (NKC), CD56bright NKC, CD56dim NKC, myeloid derived suppressor cell, NK T cell, macrophage, eosinophil, mast cell and Monocyte were substantially different in the H- and L-risk groups (**Figure 10A**). Then we compared the relation between risk score and the expression of immune checkpoint, the results revealed that the expression of TNFSF4, SIRPA, CD276 and TNFSF15 in the H- risk group were substantially greater (**Figure 10B**), indicated that high risk individuals have a potential response to the immunotherapy by targeting them.

### Correlation between the ALPS and LC therapy

**Figure 11:**
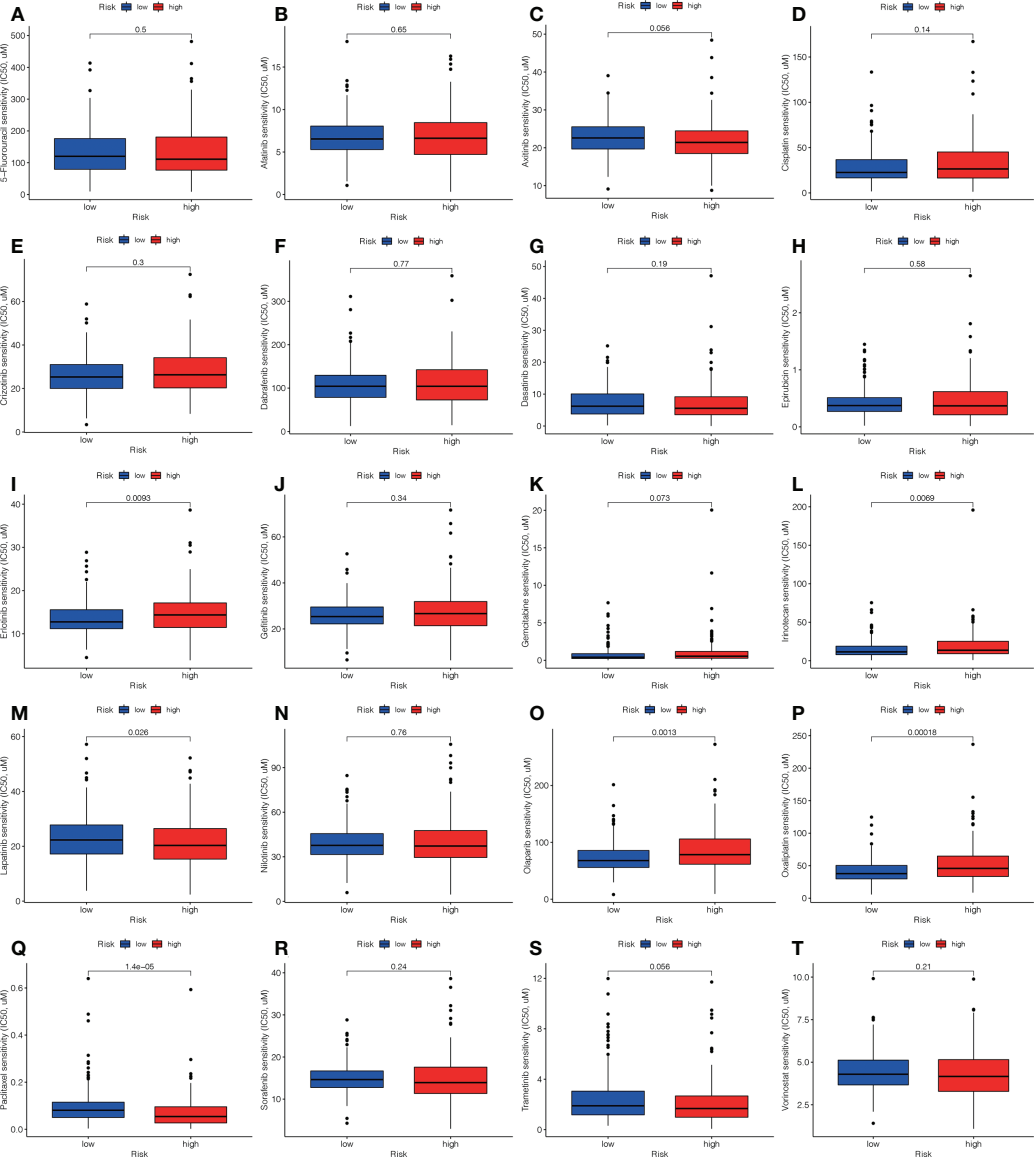
Comparison of the treatment medication sensitivity of individuals at high (H)- and low (L)-risk. IC50s of **(A)** 5-fluorouracil, of **(B)** afatinib, of **(C)** axitinib, of **(D)** cisplatin, of **(E)** crizotinib, of **(F)** dabrafenib, of **(G)** dasatinib, of **(H)** epirubicin, of **(I)** erlotinib, of **(J)** gefitinib, of **(K)** gemcitabine, of **(L)** irinotecan, of **(M)** lapatinib, of **(N)** nilotinib, of **(O)** olaparib, of **(P)** oxaliplatin, of **(Q)** paclitaxel, of **(R)** sorafenib, of **(S)** trametinib, of **(T)** vorinostat in H and L risk groups.

In addition to immuno-therapy, we investigated the relationship between the ALPS and the effectiveness of conventional chemotherapy for LC. In the L-risk group, the IC50s of erlotinib, irinotecan, olaparib and oxaliplatin were shown to be lower, while the IC50s of lapatinib and paclitaxel were shown to be lower in H-risk group (**Figure 11**). This information was useful for investigating therapeutic alternatives for H- and L- risk groups.

### Principal component analysis and GSEA

**Figure 12:**
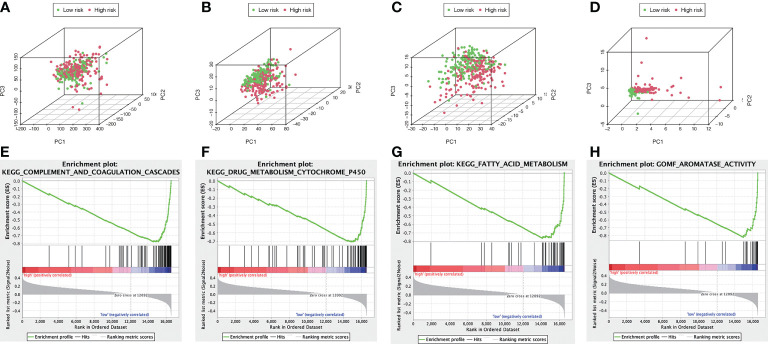
The metabolic condition of individuals with high and low risk scores varies. PCA maps depict the allocation of individuals according to the **(A)** whole genes **(B)** AME-associated gene sets; **(C)** AME-associated lncRNAs; and **(D)** the ALPS. GSEA showed significant enrichment of the **(E)** complement and coagulation cascades, **(F)** drug metabolism cytochrome p450, **(G)** fatty acid metabolism, **(H)** aromatase activity.

We visualized the individuals distribution according to the whole genome, AME-correlated gene sets, AME-related lncRNAs, and the ALPS using PCA maps. According to our findings, the ALPS was the best for individuals. Due to the disparate prognoses of individuals in the H- and L- risk groups, we utilized GSEA to investigate potential variation between the both groups (**Figures 12A–D**). Our outcomes revealed that complement and coagulation cascades, drug metabolism cytochrome p450, fatty acid metabolism, retinol metabolism, linoleic acid metabolism, tryptophan metabolism, glycine serine and threonine metabolism, primary bile acid biosynthesis, PPAR signaling pathway, valine leucine and isoleucine degradation, peroxisome, metabolism of xenobiotics by cytochrome p450, steroid hormone biosynthesis, tyrosine metabolism, histidine metabolism, arachidonic acid metabolism, adipocytokine signaling pathway and aromatase activity were substantially enriched in the low risk group (**Figures 12E–H** and **Table 2**), demonstrating that L- risk individuals are tightly correlated to metabolism of different acids and PPAR signaling pathway.

**Table 2:** The high-risk group enriched gene sets.

Gene set	ES	NES	NOM *p*-val	FDR *q*-val
Complement and coagulation cascades	-0.780	-1.984	0.002	0.129
Drug metabolism cytochrome p450	-0.705	-1.928	0.002	0.114
Fatty acid metabolism	-0.765	-1.877	0.016	0.134
Retinol metabolism	-0.708	-1.863	0.002	0.113
Linoleic acid metabolism	-0.641	-1.848	0.002	0.104
Tryptophan metabolism	-0.675	-1.835	0.002	0.096
Glycine serine and threonine metabolism	-0.766	-1.821	0.008	0.092
Primary bile acid biosynthesis	-0.853	-1.759	0.006	0.126
PPAR signaling pathway	-0.588	-1.741	0.011	0.131
Valine leucine and isoleucine degradation	-0.679	-1.706	0.049	0.151
Peroxisome	-0.567	-1.694	0.046	0.150
Metabolism of xenobiotics by cytochrome p450	-0.598	-1.686	0.039	0.135
Steroid hormone biosynthesis	-0.596	-1.673	0.035	0.136
Tyrosine metabolism	-0.527	-1.637	0.048	0.152
Arachidonic acid metabolism	-0.463	-1.573	0.023	0.179
Adipocytokine signaling pathway	-0.400	-1.512	0.028	0.212
Aromatase activity	-0.824	-1.943	0.004	0.245

ES, enrichment score; NES, normalized enrichment score; NOM, nominal; FDR, false discovery rate; PPAR, peroxisome proliferators-activated receptor.

### Validation of expression of the ALPS

**Figure 13:**
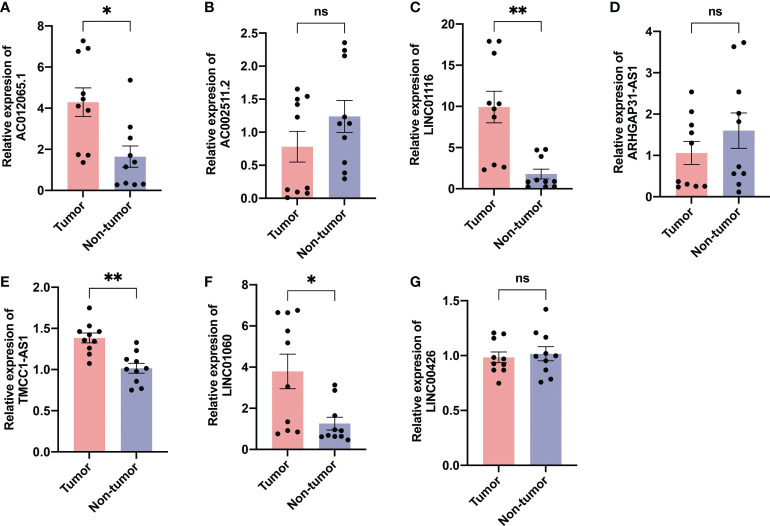
Validation of expression of the ALPS in human tissues. Expression analysis of **(A)** AC012065.1, **(B)** AC002511.2, **(C)** LINC01116, **(D)** ARHGAP31-AS1, **(E)** TMCC1-AS1, **(F)** LINC01060 and **(G)** LINC00426 in 10 pairs of liver cancer tissue samples. *P < 0.05; **P < 0.01; ns, not significant.

We evaluated the expression level of the seven AME-related lncRNAs in clinical samples retrieved from HCC patients in our hospital. Interestingly, the qRT-PCR results indicated that the expression of AC012065.1, LINC01116, TMCC1-AS1 and LINC01060, was significantly higher in tumor tissue while expression of AC002511.2, LINC00426 and ARHGAP31-AS1 was similar between tumor and normal tissue (**Figures 13A–G**). Considering the tight association between our risk model and metabolism and immunity, we believe that the metabolic and immune environment surrounding tumor cells may affect lncRNA expression. According to these results, we speculate that AME-related lncRNAs, especially the four differentially expressed lncRNAs, may play a role in liver cancer regulation.

## Discussion

Liver cancer is one of the most prevalent malignant tumors of digestive system, majority of which starting from hepatocytes. The effect of the AME in tumor is complicated. More and more investigations have shown that AME has a vital part in the incidence and development of tumor, yet the present investigation focuses mainly on the contribution of AME in tumor therapy. There is few research on its role in tumor prognosis. In recent years, there have been no studies to predict the prognosis of LC individuals by establishing lncRNA predictive features related to AME.

In the current investigation, we first gathered 788 AME-associated genes and 542 AME-related lncRNAs. KEGG findings revealed that the DEGs were primarily the DEGs were mainly enriched in AGE-RAGE, PI3K-Akt as well as HIF-1 signaling pathway. According to studies, sRAGE and CML-AGE levels are inversely correlated to the development of HCC (18). Wu et al. reported that the oncogenic PI3K/AKT/mTOR pathway was a typical dysregulated pathway in the pathogenesis of HCC (19). Vincent et al. identified that hypoxia drove the stabilization of hypoxia-inducible factors (HIFs) that behave as central regulators to suppress dampen the innate immune system of LC (20). Such findings show that AME-associated genes could regulate the progress of LC *via* the AGE-RAGE and PI3K/AKT/mTOR pathway. Additionally, AME-related genes were also associated with hypoxia. Even so, more studies are needed to validate the function of AME-associated genes in LC.

Numerous research has reported that a strongly extracellular acidic environment can cause double-stranded DNA breaks and clastogenic effects (21). Research in cell culture as well as *in vivo* also supported the notion that acidosis could affect tumor cell epigenetics and RNA processing (22). Thus, it can be seen that AME-associated lncRNAs might have a significant part in LC. We allocated 345 LC participants into three groups: a training, testing, and combined. 136 lncRNAs were identified as being associated with the prediction of LC individuals. LINC01116, AC002511.2, LINC00426, ARHGAP31-AS1, LINC01060, TMCC1-AS1 and AC012065.1 were included in the ALPS built for the training group using LASSO and MCR analysis. In all three groups, individuals with a high-risk score had a shorter OS compared to those with a low-risk score. According to the ROC analysis, the ALPS accurately predicted the prognosis of LC in all three groups. Multivariate Cox analysis showed a significant independent relationship between the ALPS and LC outcome. Lastly, we developed a nomogram to estimate the one-, three-, and five-year survival of individuals with LC. In addition, the calibration curves demonstrated that the nomogram had excellent predictive ability. Of these seven lncRNAs, LINC01116 was tightly associated to immune regulation. LINC01116 could stimulate cell proliferation, cell cycle progression, and tumor metastasis in HCC (23). Shi et al. found that LINC01060 prevented pancreatic cancer growth and invasion *in-vitro* and *in-vivo* through modulating vinculin expression (24). Li et al. reported that exosomes containing LINC01060 from hypoxic glioma stem cells increase glioma growth *via* modulating the MZF1/c-Myc/HIF1α axis (25). Study showed that autophagy-related TMCC1-AS1 predicted poor prognosis in LC (26). In addition, the importance of AC002511.2, LINC00426, ARHGAP31-AS1, LINC01060 in LC is seldom documented in the literature; hence, future study will concentrate on these 3 lncRNAs. GSEA revealed that metabolism of different acids and the PPAR signaling pathway are tightly associated with low-risk individuals. The impact of the complement and coagulation cascades signaling pathway in the aetiology of malignancies is yet unknown. Zhang et al. identified that C8B in the complement and coagulation cascades signaling pathway is a survival indicator in HBV-associated HCC individuals (27). Khamis et al. reported that cytochrome P450-2D6 enzyme (CYP2D6) might act as a putative marker in LC health inequalities, with a negative correlation to IL6 proclaimed a complex relation between CYP2D6 and inflammation in the ethnic differences observed in Asian Americans and Caucasian Americans LC individuals (28). Seo et al. indicated that fatty-acid-induced FABP5 overexpression caused HCC development *via* HIF-1-driven reprogramming of lipid metabolism (29). Lai et al. suggested that greater -carotene and retinol levels are correlated with incidence LC (30). Brown et al. reported that carnitine palmitoyltransferase gene overexpression with linoleic acid promotes CD4+ T cell apoptosis hence promoting the development of HCC (31). Cui et al. concluded that ERRFI1-induced apoptosis rendered HCC cells more sensitive to tryptophan shortage, and ERRFI1 interacted with PDCD2 to cause apoptosis in HCC cells (32). Thomas et al. identified that increased primary bile acids and taurine over glycine-conjugated ratios were highly correlated with HCC risk, while the secondary bile acids over primary bile acids ratios were inversely correlated with HCC risk (33). By decreasing PPAR-mediated glycolysis, simvastatin re-sensitizes HCC cells to sorafenib, according to Feng et al. (34). Therefore, these metabolic changes may account for the good prognosis in the L-risk group.

The acidic microenvironment of tumors also has a significant influence in drug research and development. When tumor cells are not well-nourished in culture, conditions like hypoxia or acidity can affect drug efficacy (35). The acid- dependent drug release avoid premature drug release at physiological pH, allowing for the successful delivery of the largest therapeutic cargo to target tumor cells (which are known to have an acidic internal pH compared to normal cells) (36). Previous studies have shown that multistage delivery nanoparticles (MDNP) are substantially more effective in targeting tumors compared to conventional delivery carriers. This is attributable to the fact that MDNP is capable of evading cellular absorption at neutral pH (as in blood), whereas it successfully penetrates cells at acidic pH (as in cancer tissues) (37). Additionally, therapeutic reversal of tumor acidity using RNAi nanoparticles can restore the anticancer capabilities of T cells and enhance checkpoint blockade treatment (38). In this research, we found most of the immune cells were higher in the low-risk group. It might indicate that in the low-risk group, more immune cells play an anti-tumor role in the tumor tissue, which makes them have a lower risk and better prognosis. Moreover, our study indicates that high-risk individuals are likely to be susceptible to some immunotherapies (including anti-TNFSF4, anti-SIRPA, anti-CD276 and anti-TNFSF15) and some conventional chemotherapy drugs (including lapatinib and paclitaxel). The findings suggest that people at high risk may benefit from the combination of relatively sensitive immuno- and chemo- therapy, which sets the foundation for accurate and personalized LC therapy.

Nevertheless, our research has two drawbacks. Firstly, we utilized only the TCGA dataset information for internal validation, and other databases are still needed for peer approval to determine the relevance of the predicted signature. Secondly, the mechanism of the AME-associated lncRNAs in LC requires further confirmation by experiments.

## Conclusion

In summary, the AME-associated lncRNA signature could independently diagnose the prognosis of LC individuals and establish a foundation for the response to medical therapy, however it will require future experimental confirmation.

## Data availability statement

The datasets presented in this study can be found in online repositories. The names of the repository/repositories and accession number(s) can be found in the article/**Supplementary material**.

## Ethics statement

The studies involving human participants were reviewed and approved by Wujin Hospital Institutional Ethical Review Board. The patients/participants provided their written informed consent to participate in this study.

## Author contributions

PJ, WX, CX, and WD: Study design, Data collection, Data analysis, Writing. LZ: Study design, Data collection. ZY: Study design, Data analysis. ZL and TS: biological experiment. WD, XX, and YT: Study design, Data collection, Data analysis, Revision. All authors contributed to the article and approved the submitted version.

## Funding

This work was supported by the Changzhou Sci&Tech Program (CJ20210013, CJ20220008), Young Talent Development Plan of Changzhou Health Commission (CZQM2020118, CZQM2021028), the Development Foundation of Affiliated Hospital of Xuzhou Medical University (XYFY2020016), Medical Research Project of Jiangsu Health Commission (No.Z2019027), Changzhou HighLevel Medical Talents Training Project (2022CZBJ105).

## Acknowledgments

Thanks for the support of Changzhou High-Level Medical Talents Training Project.

## Conflict of interest

The authors declare that the research was conducted in the absence of any commercial or financial relationships that could be construed as a potential conflict of interest.

## Publisher’s note

All claims expressed in this article are solely those of the authors and do not necessarily represent those of their affiliated organizations, or those of the publisher, the editors and the reviewers. Any product that may be evaluated in this article, or claim that may be made by its manufacturer, is not guaranteed or endorsed by the publisher.
