# Supplementary material for: A new acidic microenvironment related lncRNA signature predicts the prognosis of liver cancer patients
Source: Front Oncol. 2022 Oct 31;12:1016721. doi: 10.3389/fonc.2022.1016721 (PMC9660327; doi:10.3389/fonc.2022.1016721)
Supplement: Supplementary file 1 [file Table_1.docx]

**Supplementary Table S1** immune checkpoints.

| Genes |
| --- |
| BTLA |
| BTNL9 |
| CD152 |
| CD160 |
| CD226 |
| CD233 |
| CD244 |
| CD27 |
| CD274 |
| CD276 |
| CD279 |
| CD28 |
| CD40LG |
| CD47 |
| CD48 |
| CD80 |
| CD86 |
| CD70 |
| CD96 |
| HLA-A |
| HLA-C |
| HLA-DMA |
| HLA-DMB |
| HLA-DOA |
| HLA-DOB |
| HLA-DPA1 |
| HLA-DPB1 |
| HLA-DRA |
| HLA-DRB1 |
| HLA-DRB5 |
| IDO2 |
| KIR2DL4 |
| SIRPA |
| TDO2 |
| TIM-3 |
| TNFSF14 |
| TNFSF15 |
| TNFSF18 |
| TNFSF4 |
| TNFSF9 |
