# Supplementary material for: A new acidic microenvironment related lncRNA signature predicts the prognosis of liver cancer patients
Source: Front Oncol. 2022 Oct 31;12:1016721. doi: 10.3389/fonc.2022.1016721 (PMC9660327; doi:10.3389/fonc.2022.1016721)
Supplement: Supplementary file 2 [file Table_2.docx]

**Supplementary Table S2: The primers for 7 AME-related lncRNAs.**

| LncRNAs | Forward | Reverse |
| --- | --- | --- |
| AC002511.2 | 5’-GCTTGTCTTGCGTGGAAATC-3’ | 5’-TGGAAAGATGACAGAGAGGCAG-3’ |
| AC012065.1 | 5’-ATGCTCTCCTGGGCTTACTCC-3’ | 5’-CATCTGTGCTTGTGTTCTTGGG-3’ |
| ARHGAP31-AS1 | 5’-CACGCAATCCCAAGACAGC-3’ | 5’-TGCCAGTGCCTAGCATAATGTT-3’ |
| GAPDH | 5’-TGAAGGTCGGAGTCAACGGATTTGGT-3’ | 5’-CATGTGGGCCATGAGGTCC ACCAC-3’ |
| LINC00426 | 5’-AGCCTCCCTTGTTAGCCACTA-3’ | 5’-CGACATTGTTGCGGGTGAT-3’ |
| LINC01060 | 5’-CCCGAAAGGAAGAAGCTATACG | 5’-TGCGACACTTTATCTAATGAGTGTG |
| LINC01116 | 5’-TTCTTTCCCTCCAAGTGATAACC-3’ | 5’-TGAATTAGCAAGTCAGCAAGTCCT-3’ |
| TMCC1-AS1 | 5’-GGTAGGGTAGCAGGTCAGCATATC-3’ | 5’-TTGTCACAGGCCAGACTACCAG-3’ |
