# Supplementary material for: A new acidic microenvironment related lncRNA signature predicts the prognosis of liver cancer patients
Source: Front Oncol. 2022 Oct 31;12:1016721. doi: 10.3389/fonc.2022.1016721 (PMC9660327; doi:10.3389/fonc.2022.1016721)
Supplement: Supplementary file 3 [file Table_3.docx]

**Supplementary Table S3** AME-associated lncRNAs.

| gene | lncRNA | cor | pvalue |
| --- | --- | --- | --- |
| DNAH8 | LINC01587 | 0.68211357 | 1.63E-52 |
| LTA | RRN3P2 | 0.54035724 | 9.74E-30 |
| PTGS2 | DLEU2L | 0.57836648 | 9.05E-35 |
| MMP1 | DLEU2L | 0.6452846 | 2.01E-45 |
| TNFRSF11A | DLEU2L | 0.51507209 | 9.95E-27 |
| GNRH1 | DLEU2L | 0.53096275 | 1.37E-28 |
| ITGA2 | DLEU2L | 0.54985533 | 6.18E-31 |
| IDO1 | LINC01260 | 0.59193997 | 9.89E-37 |
| SIGLEC7 | LINC01260 | 0.63033782 | 8.14E-43 |
| BDNF | LINC01260 | 0.51237249 | 2.02E-26 |
| CD4 | LINC01260 | 0.51699818 | 5.99E-27 |
| CTLA4 | LINC01260 | 0.63959001 | 2.06E-44 |
| ADA | LINC01260 | 0.55773492 | 5.86E-32 |
| CCL3 | LINC01260 | 0.58030302 | 4.81E-35 |
| LTA | LINC01260 | 0.72347308 | 7.77E-62 |
| CYP1A2 | LINC00525 | 0.52015679 | 2.59E-27 |
| AKR1C1 | PART1 | 0.54131179 | 7.41E-30 |
| NCOR2 | C1orf147 | 0.50060796 | 4.07E-25 |
| PRKCD | C1orf147 | 0.56600145 | 4.63E-33 |
| FABP5 | LINC01116 | 0.65030675 | 2.48E-46 |
| PLAU | LINC01116 | 0.78886661 | 1.16E-80 |
| IL10 | LINC01116 | 0.72584201 | 2.02E-62 |
| BIRC5 | LINC01116 | 0.56561473 | 5.22E-33 |
| SLC38A5 | LINC01116 | 0.51160203 | 2.46E-26 |
| GJA1 | LINC01116 | 0.69863811 | 4.79E-56 |
| NES | LINC01116 | 0.68446185 | 5.31E-53 |
| ACACA | SNHG16 | 0.52038057 | 2.44E-27 |
| CDK4 | SNHG16 | 0.50112861 | 3.57E-25 |
| NPM1 | SNHG16 | 0.53721239 | 2.38E-29 |
| RPLP0 | SNHG16 | 0.52914907 | 2.26E-28 |
| BGLAP | SNHG11 | 0.51042647 | 3.34E-26 |
| FGFR3 | SNHG11 | 0.51168874 | 2.41E-26 |
| FGFR4 | SNHG11 | 0.56297282 | 1.18E-32 |
| LPAR2 | TMEM51-AS1 | 0.50147035 | 3.28E-25 |
| CS | TMEM51-AS1 | 0.50873819 | 5.16E-26 |
| TFAP2A | TMEM51-AS1 | 0.51859121 | 3.93E-27 |
| GNRH1 | TMEM51-AS1 | 0.56989803 | 1.36E-33 |
| PKM | TMEM51-AS1 | 0.51415891 | 1.26E-26 |
| HAS3 | TMEM51-AS1 | 0.56193349 | 1.63E-32 |
| LPAR2 | LINC01106 | 0.54009076 | 1.05E-29 |
| ABCC1 | LINC01106 | 0.6307458 | 6.94E-43 |
| TFAP2A | LINC01106 | 0.53110301 | 1.32E-28 |
| GNRH1 | LINC01106 | 0.54722157 | 1.34E-30 |
| PKM | LINC01106 | 0.54944611 | 6.97E-31 |
| NPM1 | DLEU1 | 0.50351664 | 1.96E-25 |
| CHGA | MIR7-3HG | 0.64105723 | 1.14E-44 |
| BCAT1 | LINC01561 | 0.62341631 | 1.17E-41 |
| BDNF | LINC01561 | 0.90178143 | 1.36E-137 |
| IL11 | LINC01561 | 0.99701806 | 0 |
| LGALS1 | LINC01561 | 0.61777142 | 9.87E-41 |
| PKM | LINC01561 | 0.56834998 | 2.22E-33 |
| STRA6 | LINC01561 | 0.84822333 | 1.02E-104 |
| AKR1C1 | DLGAP1-AS1 | 0.5713511 | 8.61E-34 |
| TKT | DLGAP1-AS1 | 0.50962904 | 4.11E-26 |
| BCAT1 | ZFAS1 | 0.50143379 | 3.31E-25 |
| BAX | ZFAS1 | 0.51226515 | 2.07E-26 |
| NPM1 | ZFAS1 | 0.5518586 | 3.42E-31 |
| CHGA | ZFAS1 | 0.57488304 | 2.79E-34 |
| RPLP0 | ZFAS1 | 0.74301127 | 7.42E-67 |
| RPLP2 | ZFAS1 | 0.74276779 | 8.62E-67 |
| ABCC1 | CASC2 | 0.57077039 | 1.03E-33 |
| BDNF | FAM87B | 0.68293652 | 1.10E-52 |
| FBN1 | FAM87B | 0.50872045 | 5.19E-26 |
| IL11 | FAM87B | 0.71878683 | 1.07E-60 |
| LGALS1 | FAM87B | 0.51488914 | 1.04E-26 |
| STRA6 | FAM87B | 0.63434976 | 1.68E-43 |
| BCAT1 | AC108142.1 | 0.56797028 | 2.50E-33 |
| PLAU | AC108142.1 | 0.67207003 | 1.77E-50 |
| IL10 | AC108142.1 | 0.68027601 | 3.90E-52 |
| BDNF | AC108142.1 | 0.74723464 | 5.31E-68 |
| COL1A1 | AC108142.1 | 0.53352705 | 6.71E-29 |
| IL11 | AC108142.1 | 0.84126467 | 2.13E-101 |
| GJA1 | AC108142.1 | 0.66819008 | 1.03E-49 |
| LGALS1 | AC108142.1 | 0.67255444 | 1.42E-50 |
| PKM | AC108142.1 | 0.66041803 | 3.24E-48 |
| STRA6 | AC108142.1 | 0.71229536 | 3.74E-59 |
| IL1B | PIK3CD-AS1 | 0.61806979 | 8.83E-41 |
| BCAT1 | PIK3CD-AS1 | 0.54721593 | 1.34E-30 |
| IL10 | PIK3CD-AS1 | 0.50108081 | 3.61E-25 |
| SIGLEC7 | PIK3CD-AS1 | 0.70165911 | 1.02E-56 |
| BDNF | PIK3CD-AS1 | 0.648321 | 5.70E-46 |
| CD4 | PIK3CD-AS1 | 0.56042311 | 2.59E-32 |
| CTLA4 | PIK3CD-AS1 | 0.63873292 | 2.91E-44 |
| IL11 | PIK3CD-AS1 | 0.54458386 | 2.89E-30 |
| CCL3 | PIK3CD-AS1 | 0.64266457 | 5.90E-45 |
| LGALS1 | PIK3CD-AS1 | 0.51860503 | 3.91E-27 |
| PKM | PIK3CD-AS1 | 0.52717138 | 3.89E-28 |
| STRA6 | PIK3CD-AS1 | 0.56213592 | 1.53E-32 |
| LTA | PIK3CD-AS1 | 0.68581652 | 2.76E-53 |
| FABP4 | ACTA2-AS1 | 0.62279164 | 1.49E-41 |
| CETP | ACTA2-AS1 | 0.51035783 | 3.40E-26 |
| IL1B | C9orf139 | 0.51168532 | 2.41E-26 |
| FABP5 | C9orf139 | 0.51035173 | 3.41E-26 |
| IL10 | C9orf139 | 0.56378875 | 9.19E-33 |
| SIGLEC7 | C9orf139 | 0.61469466 | 3.09E-40 |
| CD4 | C9orf139 | 0.50271006 | 2.40E-25 |
| CTLA4 | C9orf139 | 0.52750881 | 3.54E-28 |
| LGALS1 | C9orf139 | 0.59066967 | 1.52E-36 |
| PKM | C9orf139 | 0.56670391 | 3.72E-33 |
| LTA | C9orf139 | 0.59761579 | 1.40E-37 |
| CS | WDFY3-AS2 | 0.54735119 | 1.29E-30 |
| PFKM | WDFY3-AS2 | 0.5474596 | 1.25E-30 |
| BRCA1 | LINC00471 | 0.55356101 | 2.06E-31 |
| HMMR | LINC00471 | 0.50143362 | 3.31E-25 |
| CNR1 | CELF2-AS1 | 0.5838728 | 1.49E-35 |
| PTGS2 | B3GALT5-AS1 | 0.67404295 | 7.16E-51 |
| MMP1 | B3GALT5-AS1 | 0.66118115 | 2.32E-48 |
| FFAR4 | LINC00313 | 0.64699615 | 9.90E-46 |
| BCAT1 | LINC00158 | 0.62794162 | 2.07E-42 |
| SIGLEC7 | LINC00158 | 0.52464171 | 7.74E-28 |
| BDNF | LINC00158 | 0.83541807 | 9.90E-99 |
| IL11 | LINC00158 | 0.88477888 | 2.05E-125 |
| LGALS1 | LINC00158 | 0.5878504 | 3.94E-36 |
| PKM | LINC00158 | 0.59120976 | 1.27E-36 |
| STRA6 | LINC00158 | 0.78782715 | 2.61E-80 |
| LTA | LINC00158 | 0.52786874 | 3.21E-28 |
| IL1B | LINC00839 | 0.52856764 | 2.65E-28 |
| FABP5 | LINC00839 | 0.51023239 | 3.51E-26 |
| BCAT1 | LINC00839 | 0.6434743 | 4.24E-45 |
| PLAU | LINC00839 | 0.52138743 | 1.86E-27 |
| IL10 | LINC00839 | 0.54934968 | 7.18E-31 |
| BDNF | LINC00839 | 0.64876808 | 4.73E-46 |
| IL11 | LINC00839 | 0.56374492 | 9.32E-33 |
| GJA1 | LINC00839 | 0.51265816 | 1.87E-26 |
| ANXA2 | LINC00839 | 0.54017994 | 1.02E-29 |
| LGALS1 | LINC00839 | 0.60433405 | 1.32E-38 |
| PKM | LINC00839 | 0.63492803 | 1.33E-43 |
| KAT2A | KTN1-AS1 | 0.51381449 | 1.38E-26 |
| FABP5 | LINC01551 | 0.51633951 | 7.13E-27 |
| PLAU | LINC01551 | 0.9679578 | 2.41E-225 |
| IL10 | LINC01551 | 0.83453837 | 2.44E-98 |
| BIRC5 | LINC01551 | 0.5606993 | 2.38E-32 |
| SLC38A5 | LINC01551 | 0.63046869 | 7.73E-43 |
| GJA1 | LINC01551 | 0.71551182 | 6.51E-60 |
| NES | LINC01551 | 0.72132029 | 2.61E-61 |
| GLS | LINC00265 | 0.52785281 | 3.22E-28 |
| SRC | LINC00265 | 0.50174981 | 3.05E-25 |
| NCOR2 | LINC00265 | 0.56224149 | 1.48E-32 |
| GNRH1 | LINC00265 | 0.5461904 | 1.81E-30 |
| ACVRL1 | C22orf34 | 0.51347134 | 1.51E-26 |
| FFAR4 | CYP51A1-AS1 | 0.61504131 | 2.72E-40 |
| ABCC1 | PAX8-AS1 | 0.56442177 | 7.56E-33 |
| ANXA2 | PAX8-AS1 | 0.50268517 | 2.41E-25 |
| TH | LINC00943 | 0.64002523 | 1.73E-44 |
| BCAT1 | LINC00943 | 0.65597071 | 2.23E-47 |
| CHGA | LINC00943 | 0.87517988 | 2.34E-119 |
| ALOX12 | LINC01560 | 0.53651919 | 2.90E-29 |
| BRCA1 | LINC01560 | 0.50259644 | 2.47E-25 |
| CS | LINC01560 | 0.5250634 | 6.90E-28 |
| NCOR2 | LINC01560 | 0.57602044 | 1.93E-34 |
| CDK4 | LINC01560 | 0.50106985 | 3.62E-25 |
| ACLY | LINC01560 | 0.53641202 | 2.99E-29 |
| RPLP0 | SNHG17 | 0.64193539 | 7.95E-45 |
| RPLP2 | SNHG17 | 0.569553 | 1.52E-33 |
| PLCG1 | RAMP2-AS1 | 0.52818502 | 2.94E-28 |
| GLS | C2orf27A | 0.50377448 | 1.83E-25 |
| CDK4 | C2orf27A | 0.57179172 | 7.48E-34 |
| PRKCD | C2orf27A | 0.52004111 | 2.67E-27 |
| FABP5 | SNHG12 | 0.51225075 | 2.08E-26 |
| KAT2A | SNHG12 | 0.52924924 | 2.20E-28 |
| GNRH1 | SNHG12 | 0.60204384 | 2.98E-38 |
| PKM | SNHG12 | 0.53440819 | 5.24E-29 |
| KAT2A | C3orf35 | 0.52170858 | 1.71E-27 |
| PPARG | PDCD4-AS1 | 0.56066405 | 2.40E-32 |
| FABP4 | PDCD4-AS1 | 0.56212027 | 1.54E-32 |
| CETP | PDCD4-AS1 | 0.51261243 | 1.89E-26 |
| GNRH1 | IGBP1-AS1 | 0.55450131 | 1.55E-31 |
| PTHLH | LINC00970 | 0.75690754 | 1.03E-70 |
| BCAT1 | LINC00862 | 0.55050376 | 5.10E-31 |
| BDNF | LINC00862 | 0.69095386 | 2.25E-54 |
| IL11 | LINC00862 | 0.74329303 | 6.23E-67 |
| LGALS1 | LINC00862 | 0.5351816 | 4.22E-29 |
| PKM | LINC00862 | 0.5425058 | 5.26E-30 |
| STRA6 | LINC00862 | 0.67222272 | 1.65E-50 |
| GLUL | LINC00272 | 0.63560769 | 1.02E-43 |
| BRCA1 | ATP1A1-AS1 | 0.56249487 | 1.37E-32 |
| HMMR | ATP1A1-AS1 | 0.52907127 | 2.31E-28 |
| RPLP0 | SNHG5 | 0.59246354 | 8.27E-37 |
| RPLP2 | SNHG5 | 0.64987162 | 2.98E-46 |
| HRAS | ARRDC1-AS1 | 0.52216533 | 1.51E-27 |
| EHMT2 | ARRDC1-AS1 | 0.56345807 | 1.02E-32 |
| CTLA4 | PSMB8-AS1 | 0.54326257 | 4.23E-30 |
| LPAR2 | LINC01123 | 0.63699234 | 5.86E-44 |
| SPP1 | LINC01123 | 0.50767019 | 6.79E-26 |
| ABCC1 | LINC01123 | 0.64100947 | 1.16E-44 |
| TGFB2 | LINC01123 | 0.54907834 | 7.77E-31 |
| TFAP2A | LINC01123 | 0.67165931 | 2.14E-50 |
| GNRH1 | LINC01123 | 0.56600779 | 4.62E-33 |
| ITGA2 | LINC01123 | 0.54403678 | 3.38E-30 |
| CS | STARD7-AS1 | 0.57812316 | 9.79E-35 |
| NCOR2 | STARD7-AS1 | 0.54449902 | 2.96E-30 |
| CDK4 | STARD7-AS1 | 0.52154367 | 1.78E-27 |
| EHMT2 | STARD7-AS1 | 0.50338693 | 2.02E-25 |
| LPAR2 | FAM201A | 0.60148015 | 3.63E-38 |
| FGFR3 | FAM201A | 0.62451612 | 7.72E-42 |
| MYCN | LINC01121 | 0.52917852 | 2.24E-28 |
| AKR1C1 | LINC01460 | 0.61696326 | 1.33E-40 |
| AKR1C3 | LINC01460 | 0.50960551 | 4.13E-26 |
| IDO1 | HCP5 | 0.53712285 | 2.44E-29 |
| CTLA4 | HCP5 | 0.50731009 | 7.45E-26 |
| KAT2A | THUMPD3-AS1 | 0.56728976 | 3.09E-33 |
| EHMT2 | THUMPD3-AS1 | 0.52235053 | 1.44E-27 |
| NCOR2 | AC253576.2 | 0.54719689 | 1.35E-30 |
| DNMT1 | AC253576.2 | 0.53883007 | 1.51E-29 |
| BGLAP | LINC01089 | 0.53895427 | 1.45E-29 |
| MAPK3 | AC016747.3 | 0.53027072 | 1.66E-28 |
| CS | AC016747.3 | 0.52893324 | 2.40E-28 |
| AKR1C1 | AC016747.3 | 0.5206161 | 2.29E-27 |
| ANXA2 | AC016747.3 | 0.51205875 | 2.19E-26 |
| AKR1C3 | AC016747.3 | 0.55430655 | 1.65E-31 |
| NPM1 | AC022210.2 | 0.54204424 | 6.01E-30 |
| RPLP0 | AC022210.2 | 0.5189519 | 3.57E-27 |
| ACACA | ZNF337-AS1 | 0.53359577 | 6.58E-29 |
| BRCA1 | ZNF337-AS1 | 0.54868937 | 8.71E-31 |
| PARP1 | ZNF337-AS1 | 0.51145592 | 2.56E-26 |
| KAT2A | ZNF337-AS1 | 0.50160438 | 3.17E-25 |
| ALOX12 | LINC01521 | 0.55448215 | 1.56E-31 |
| PRKCA | LINC01521 | 0.577774 | 1.10E-34 |
| CS | LINC01521 | 0.55788634 | 5.60E-32 |
| SLC22A5 | AC074286.1 | 0.52278523 | 1.28E-27 |
| FFAR4 | UCA1 | 0.75166338 | 3.15E-69 |
| HSPG2 | PAXIP1-AS2 | 0.50999711 | 3.73E-26 |
| SLC22A5 | AC024560.3 | 0.5078743 | 6.45E-26 |
| BRCA1 | AC024560.3 | 0.54845439 | 9.34E-31 |
| NCOR2 | AC024560.3 | 0.54973233 | 6.41E-31 |
| KAT2A | AC024560.3 | 0.51473459 | 1.09E-26 |
| DNMT1 | AC024560.3 | 0.54443608 | 3.01E-30 |
| GNRH1 | AC024560.3 | 0.50311969 | 2.16E-25 |
| EZH2 | AC024560.3 | 0.52739256 | 3.66E-28 |
| SLC22A5 | AC005562.1 | 0.51483026 | 1.06E-26 |
| GLS | AC005562.1 | 0.5146756 | 1.10E-26 |
| BRCA1 | AC005562.1 | 0.60344865 | 1.81E-38 |
| PRKCA | AC005562.1 | 0.51854911 | 3.97E-27 |
| PARP1 | AC005562.1 | 0.53683098 | 2.65E-29 |
| NCOR2 | AC005562.1 | 0.51277871 | 1.81E-26 |
| DNMT1 | AC005562.1 | 0.55762608 | 6.06E-32 |
| CAMK2G | AC005562.1 | 0.54719075 | 1.35E-30 |
| ACLY | AC005562.1 | 0.52840657 | 2.77E-28 |
| EZH2 | AC005562.1 | 0.5016982 | 3.09E-25 |
| RPLP0 | AC010468.1 | 0.56842769 | 2.17E-33 |
| RPLP2 | AC010468.1 | 0.50383259 | 1.81E-25 |
| LTA | LINC00243 | 0.79686632 | 2.00E-83 |
| PCNA | HLA-F-AS1 | 0.50211495 | 2.79E-25 |
| MAPK3 | CD27-AS1 | 0.56552893 | 5.36E-33 |
| CS | CD27-AS1 | 0.57579114 | 2.08E-34 |
| ABCC1 | CD27-AS1 | 0.54624703 | 1.78E-30 |
| PFKM | CD27-AS1 | 0.52328262 | 1.12E-27 |
| ANXA2 | CD27-AS1 | 0.50266121 | 2.43E-25 |
| GNRH1 | CD27-AS1 | 0.50592369 | 1.06E-25 |
| PKM | CD27-AS1 | 0.50059387 | 4.08E-25 |
| DVL2 | ALOX12-AS1 | 0.66538577 | 3.62E-49 |
| ACSL1 | DHRS4-AS1 | 0.52015569 | 2.59E-27 |
| ACACA | MCM3AP-AS1 | 0.52369647 | 9.99E-28 |
| BRCA1 | MCM3AP-AS1 | 0.53747808 | 2.21E-29 |
| CS | MCM3AP-AS1 | 0.51869224 | 3.82E-27 |
| FGFR3 | MCM3AP-AS1 | 0.53626831 | 3.11E-29 |
| NCOR2 | MCM3AP-AS1 | 0.54561579 | 2.14E-30 |
| KAT2A | MCM3AP-AS1 | 0.556243 | 9.20E-32 |
| CDK4 | MCM3AP-AS1 | 0.55578994 | 1.05E-31 |
| EHMT2 | MCM3AP-AS1 | 0.53601337 | 3.34E-29 |
| DNMT1 | MCM3AP-AS1 | 0.55398076 | 1.82E-31 |
| CAMK2G | MCM3AP-AS1 | 0.54447577 | 2.98E-30 |
| ACLY | MCM3AP-AS1 | 0.58055684 | 4.43E-35 |
| EZH2 | MCM3AP-AS1 | 0.51698481 | 6.01E-27 |
| DVL2 | MCM3AP-AS1 | 0.50032281 | 4.37E-25 |
| CEL | AATBC | 0.52139301 | 1.86E-27 |
| CNR1 | LINC01139 | 0.62191465 | 2.08E-41 |
| IL1B | C7orf65 | 0.52394839 | 9.34E-28 |
| ABCC1 | C7orf65 | 0.60221205 | 2.80E-38 |
| CYP3A4 | LINC01124 | 0.61115019 | 1.14E-39 |
| GLUL | LINC01124 | 0.68466928 | 4.81E-53 |
| BMP4 | LINC01124 | 0.59815155 | 1.16E-37 |
| GNRH1 | AC005104.3 | 0.59068573 | 1.51E-36 |
| PTGS2 | CLDN10-AS1 | 0.51927324 | 3.28E-27 |
| MMP1 | CLDN10-AS1 | 0.62109956 | 2.83E-41 |
| ITGA2 | CLDN10-AS1 | 0.5043848 | 1.57E-25 |
| CS | NUTM2A-AS1 | 0.53695367 | 2.56E-29 |
| PARP1 | NUTM2A-AS1 | 0.50550443 | 1.18E-25 |
| NCOR2 | NUTM2A-AS1 | 0.53948656 | 1.25E-29 |
| CDK4 | NUTM2A-AS1 | 0.57141595 | 8.43E-34 |
| NPM1 | NUTM2A-AS1 | 0.50170041 | 3.09E-25 |
| EHMT2 | NUTM2A-AS1 | 0.51939428 | 3.17E-27 |
| RPLP0 | NUTM2A-AS1 | 0.52780616 | 3.27E-28 |
| CAMK2G | NUTM2A-AS1 | 0.57112795 | 9.24E-34 |
| FGFR4 | NUTM2A-AS1 | 0.5063668 | 9.48E-26 |
| DVL2 | NUTM2A-AS1 | 0.53665821 | 2.79E-29 |
| GNRH1 | AC093690.1 | 0.50904411 | 4.77E-26 |
| IL1B | HLA-DQB1-AS1 | 0.55213409 | 3.15E-31 |
| IDO1 | HLA-DQB1-AS1 | 0.56418546 | 8.13E-33 |
| SIGLEC7 | HLA-DQB1-AS1 | 0.54366738 | 3.76E-30 |
| CTLA4 | HLA-DQB1-AS1 | 0.62121367 | 2.71E-41 |
| CCL3 | HLA-DQB1-AS1 | 0.50133306 | 3.39E-25 |
| ALOX12 | LINC00630 | 0.50226386 | 2.68E-25 |
| SLC22A5 | LINC00630 | 0.53125969 | 1.26E-28 |
| BRCA1 | LINC00630 | 0.60946638 | 2.09E-39 |
| PRKCA | LINC00630 | 0.5266109 | 4.53E-28 |
| CS | LINC00630 | 0.55653144 | 8.44E-32 |
| NCOR2 | LINC00630 | 0.6142334 | 3.67E-40 |
| DNMT1 | LINC00630 | 0.5825384 | 2.31E-35 |
| CDK1 | LINC00630 | 0.5154681 | 8.97E-27 |
| TNFRSF11A | LINC00630 | 0.51308728 | 1.67E-26 |
| MKI67 | LINC00630 | 0.54456655 | 2.90E-30 |
| CAMK2G | LINC00630 | 0.50081264 | 3.86E-25 |
| BCHE | LINC01208 | 0.51714489 | 5.76E-27 |
| FABP4 | MIR503HG | 0.61956461 | 5.04E-41 |
| CETP | MIR503HG | 0.60990371 | 1.79E-39 |
| HCAR1 | MIR503HG | 0.60440332 | 1.29E-38 |
| TYRP1 | MIR503HG | 0.55409223 | 1.76E-31 |
| RNASE1 | MIR503HG | 0.5709231 | 9.86E-34 |
| KAT2A | LINC00205 | 0.56791449 | 2.54E-33 |
| EHMT2 | LINC00205 | 0.55094491 | 4.48E-31 |
| CDK1 | LINC00205 | 0.52489311 | 7.23E-28 |
| DVL2 | LINC00205 | 0.50421082 | 1.64E-25 |
| GLUL | LGALS8-AS1 | 0.53726549 | 2.35E-29 |
| PTGS2 | LINC01342 | 0.54772062 | 1.16E-30 |
| MMP1 | LINC01342 | 0.66858685 | 8.62E-50 |
| TFAP2A | LINC01342 | 0.52716048 | 3.90E-28 |
| BCAT1 | ROR1-AS1 | 0.63012066 | 8.86E-43 |
| BDNF | ROR1-AS1 | 0.90313461 | 1.17E-138 |
| COL1A1 | ROR1-AS1 | 0.51459582 | 1.13E-26 |
| IL11 | ROR1-AS1 | 0.98590964 | 5.53E-291 |
| LGALS1 | ROR1-AS1 | 0.66200574 | 1.62E-48 |
| PKM | ROR1-AS1 | 0.59883577 | 9.17E-38 |
| STRA6 | ROR1-AS1 | 0.85024239 | 1.03E-105 |
| GNRH1 | MIR181A2HG | 0.53904038 | 1.42E-29 |
| SOX9 | MIR181A2HG | 0.59166115 | 1.09E-36 |
| NPM1 | EPB41L4A-AS1 | 0.60525983 | 9.50E-39 |
| RPLP0 | EPB41L4A-AS1 | 0.66437369 | 5.68E-49 |
| FGFR4 | EPB41L4A-AS1 | 0.51248362 | 1.96E-26 |
| RPLP2 | EPB41L4A-AS1 | 0.55136062 | 3.96E-31 |
| COL2A1 | EGFR-AS1 | 0.51334923 | 1.56E-26 |
| FABP4 | WARS2-IT1 | 0.50162217 | 3.15E-25 |
| CETP | WARS2-IT1 | 0.50659992 | 8.93E-26 |
| TYRP1 | WARS2-IT1 | 0.50674583 | 8.61E-26 |
| PTGS2 | LINC01133 | 0.57173068 | 7.63E-34 |
| MMP1 | LINC01133 | 0.65790481 | 9.69E-48 |
| BRCA1 | PRKAR2A-AS1 | 0.54764491 | 1.18E-30 |
| HMMR | PRKAR2A-AS1 | 0.5334506 | 6.85E-29 |
| IL4I1 | LINC00539 | 0.50162083 | 3.15E-25 |
| SIGLEC7 | LINC00539 | 0.62861421 | 1.59E-42 |
| BDNF | LINC00539 | 0.53378659 | 6.24E-29 |
| CD4 | LINC00539 | 0.5416974 | 6.64E-30 |
| CTLA4 | LINC00539 | 0.73460719 | 1.21E-64 |
| ADA | LINC00539 | 0.50370773 | 1.86E-25 |
| CCL3 | LINC00539 | 0.55839789 | 4.80E-32 |
| PRKCD | LINC00539 | 0.51448105 | 1.16E-26 |
| LTA | LINC00539 | 0.61505516 | 2.71E-40 |
| ESR1 | HTR2A-AS1 | 0.53822817 | 1.79E-29 |
| PLAU | LINC01117 | 0.57053858 | 1.11E-33 |
| IL10 | LINC01117 | 0.55326985 | 2.24E-31 |
| GJA1 | LINC01117 | 0.56158061 | 1.82E-32 |
| NES | LINC01117 | 0.52191101 | 1.62E-27 |
| RPLP2 | AC007387.2 | 0.50281162 | 2.34E-25 |
| PRKAA2 | RASAL2-AS1 | 0.54119941 | 7.66E-30 |
| CNR1 | AC090945.1 | 0.50084345 | 3.83E-25 |
| AGPAT4 | AC090945.1 | 0.51792942 | 4.68E-27 |
| GAP43 | AC090945.1 | 0.51905129 | 3.47E-27 |
| IL1RAPL2 | AC015987.1 | 0.57064748 | 1.08E-33 |
| HAS3 | AC015987.1 | 0.68195607 | 1.76E-52 |
| MMP9 | TMEM72-AS1 | 0.64422754 | 3.11E-45 |
| ABCC1 | TMEM72-AS1 | 0.52390777 | 9.44E-28 |
| TGFB2 | TMEM72-AS1 | 0.73649854 | 3.92E-65 |
| TFAP2A | TMEM72-AS1 | 0.56519288 | 5.95E-33 |
| IBSP | TMEM72-AS1 | 0.69123242 | 1.96E-54 |
| REN | AC011754.1 | 0.50376254 | 1.84E-25 |
| CAMK2G | LINC00863 | 0.56893605 | 1.85E-33 |
| TH | PGM5-AS1 | 0.50066996 | 4.00E-25 |
| CHGA | PGM5-AS1 | 0.67245644 | 1.48E-50 |
| SLC22A5 | INE1 | 0.52414636 | 8.85E-28 |
| NCOR2 | INE1 | 0.52428037 | 8.53E-28 |
| CAMK2G | INE1 | 0.50539504 | 1.21E-25 |
| GNRH1 | INE1 | 0.5392539 | 1.33E-29 |
| SPARC | AC000067.1 | 0.50625906 | 9.75E-26 |
| NES | AC000067.1 | 0.50753888 | 7.03E-26 |
| MMP1 | MIR137HG | 0.6166899 | 1.48E-40 |
| TFAP2A | TRAPPC12-AS1 | 0.57080743 | 1.02E-33 |
| ITGA2 | TRAPPC12-AS1 | 0.50385227 | 1.80E-25 |
| HAS3 | TRAPPC12-AS1 | 0.51677347 | 6.36E-27 |
| PLAU | LINC00705 | 0.67431353 | 6.32E-51 |
| IL10 | LINC00705 | 0.60365399 | 1.68E-38 |
| BIRC5 | LINC00705 | 0.53781235 | 2.01E-29 |
| GJA1 | LINC00705 | 0.55162343 | 3.66E-31 |
| NES | LINC00705 | 0.54926094 | 7.37E-31 |
| GNRH1 | LHFPL3-AS2 | 0.59476888 | 3.75E-37 |
| OXT | AC078942.1 | 0.62486003 | 6.77E-42 |
| MAPK3 | NRSN2-AS1 | 0.50200687 | 2.86E-25 |
| CS | NRSN2-AS1 | 0.58043326 | 4.61E-35 |
| ABCC1 | NRSN2-AS1 | 0.57433958 | 3.32E-34 |
| PFKM | NRSN2-AS1 | 0.69811258 | 6.25E-56 |
| PKM | NRSN2-AS1 | 0.50874481 | 5.16E-26 |
| BCAT1 | BOLA3-AS1 | 0.59289868 | 7.13E-37 |
| CDK4 | BOLA3-AS1 | 0.52831452 | 2.84E-28 |
| CHGA | BOLA3-AS1 | 0.59649145 | 2.07E-37 |
| PKM | BOLA3-AS1 | 0.51566638 | 8.51E-27 |
| NES | BOLA3-AS1 | 0.53373104 | 6.34E-29 |
| FGFR3 | NUTM2B-AS1 | 0.5381226 | 1.84E-29 |
| CDK4 | NUTM2B-AS1 | 0.50074462 | 3.93E-25 |
| TGFB2 | NUTM2B-AS1 | 0.52001702 | 2.69E-27 |
| CAMK2G | NUTM2B-AS1 | 0.62408308 | 9.11E-42 |
| SOX9 | NUTM2B-AS1 | 0.5219492 | 1.60E-27 |
| CNR1 | ZNF385D-AS1 | 0.50806572 | 6.14E-26 |
| FABP5 | FAM225B | 0.50446562 | 1.54E-25 |
| PLAU | FAM225B | 0.54742559 | 1.26E-30 |
| IL10 | FAM225B | 0.58133282 | 3.43E-35 |
| BDNF | FAM225B | 0.60045252 | 5.21E-38 |
| IL11 | FAM225B | 0.67169082 | 2.11E-50 |
| GJA1 | FAM225B | 0.58164198 | 3.10E-35 |
| LGALS1 | FAM225B | 0.69374884 | 5.63E-55 |
| PKM | FAM225B | 0.66543001 | 3.55E-49 |
| STRA6 | FAM225B | 0.63400912 | 1.92E-43 |
| ACACA | FGD5-AS1 | 0.54525194 | 2.38E-30 |
| SLC22A5 | FGD5-AS1 | 0.5329139 | 7.96E-29 |
| BRCA1 | FGD5-AS1 | 0.60725365 | 4.65E-39 |
| PRKCA | FGD5-AS1 | 0.53924256 | 1.34E-29 |
| CS | FGD5-AS1 | 0.52748747 | 3.56E-28 |
| NCOR2 | FGD5-AS1 | 0.64136942 | 1.00E-44 |
| CDK4 | FGD5-AS1 | 0.51266402 | 1.87E-26 |
| DNMT1 | FGD5-AS1 | 0.53384699 | 6.14E-29 |
| PLCG1 | FGD5-AS1 | 0.55795825 | 5.48E-32 |
| HMMR | FGD5-AS1 | 0.50472316 | 1.44E-25 |
| CAMK2G | FGD5-AS1 | 0.60501701 | 1.04E-38 |
| ACLY | FGD5-AS1 | 0.63448011 | 1.59E-43 |
| DVL2 | FGD5-AS1 | 0.50110692 | 3.59E-25 |
| SLC6A3 | PLAC4 | 0.68869721 | 6.82E-54 |
| FABP5 | MIAT | 0.54250584 | 5.26E-30 |
| PLAU | MIAT | 0.95616191 | 1.67E-200 |
| IL10 | MIAT | 0.89420191 | 6.59E-132 |
| BIRC5 | MIAT | 0.55396692 | 1.82E-31 |
| SLC38A5 | MIAT | 0.59175988 | 1.05E-36 |
| GJA1 | MIAT | 0.74374589 | 4.71E-67 |
| NES | MIAT | 0.68912769 | 5.52E-54 |
| KAT2A | RUSC1-AS1 | 0.50894234 | 4.90E-26 |
| IL1B | AC023590.1 | 0.50917757 | 4.61E-26 |
| SIGLEC7 | AC023590.1 | 0.57008547 | 1.28E-33 |
| CTLA4 | AC023590.1 | 0.64639603 | 1.27E-45 |
| ADA | AC023590.1 | 0.50328353 | 2.07E-25 |
| CCL3 | AC023590.1 | 0.53350827 | 6.74E-29 |
| LTA | AC023590.1 | 0.54032298 | 9.84E-30 |
| TH | LAMP5-AS1 | 0.67694337 | 1.87E-51 |
| BCAT1 | LAMP5-AS1 | 0.56872248 | 1.97E-33 |
| KCNJ11 | LAMP5-AS1 | 0.55418812 | 1.71E-31 |
| CHGA | LAMP5-AS1 | 0.91028316 | 1.50E-144 |
| FABP5 | LINC00937 | 0.5779234 | 1.04E-34 |
| PLAU | LINC00937 | 0.70380905 | 3.34E-57 |
| IL10 | LINC00937 | 0.71701836 | 2.85E-60 |
| BIRC5 | LINC00937 | 0.53403586 | 5.82E-29 |
| GJA1 | LINC00937 | 0.60146282 | 3.65E-38 |
| PKM | LINC00937 | 0.5089676 | 4.87E-26 |
| NES | LINC00937 | 0.50455085 | 1.51E-25 |
| PTGS2 | AP4B1-AS1 | 0.57127159 | 8.83E-34 |
| MMP1 | AP4B1-AS1 | 0.65238391 | 1.03E-46 |
| GNRH1 | AP4B1-AS1 | 0.50083212 | 3.85E-25 |
| MMP9 | ZNF32-AS1 | 0.54350784 | 3.94E-30 |
| FGFR3 | ZNF32-AS1 | 0.53688583 | 2.61E-29 |
| TGFB2 | ZNF32-AS1 | 0.61804103 | 8.93E-41 |
| TFAP2A | ZNF32-AS1 | 0.60190003 | 3.13E-38 |
| CAMK2G | ZNF32-AS1 | 0.57310438 | 4.93E-34 |
| GNRH1 | ZNF32-AS1 | 0.5670141 | 3.37E-33 |
| IBSP | ZNF32-AS1 | 0.57150593 | 8.19E-34 |
| AGPAT4 | ISM1-AS1 | 0.51369634 | 1.43E-26 |
| AKR1C1 | LINC00351 | 0.5510513 | 4.34E-31 |
| LGALS1 | AC058791.1 | 0.5230275 | 1.20E-27 |
| PKM | AC058791.1 | 0.57640266 | 1.71E-34 |
| TKT | SLC16A1-AS1 | 0.52683684 | 4.26E-28 |
| SLC6A3 | LINC00323 | 0.57291355 | 5.24E-34 |
| HAS3 | LINC00323 | 0.69350289 | 6.36E-55 |
| CNR1 | AC126365.1 | 0.54493339 | 2.61E-30 |
| PLCG1 | PLCG1-AS1 | 0.69907454 | 3.83E-56 |
| CNR1 | TEX41 | 0.68516925 | 3.78E-53 |
| ALOX12 | ENTPD1-AS1 | 0.50054749 | 4.13E-25 |
| BRCA1 | ENTPD1-AS1 | 0.50366715 | 1.88E-25 |
| PRKCA | ENTPD1-AS1 | 0.52707826 | 3.99E-28 |
| NCOR2 | ENTPD1-AS1 | 0.51138389 | 2.61E-26 |
| PLCG1 | ENTPD1-AS1 | 0.50452353 | 1.52E-25 |
| CAMK2G | ENTPD1-AS1 | 0.56701592 | 3.37E-33 |
| KAT2A | LENG8-AS1 | 0.61059056 | 1.39E-39 |
| GNRH1 | LENG8-AS1 | 0.60828261 | 3.21E-39 |
| GLS | FAM66C | 0.53749232 | 2.20E-29 |
| GNRH1 | FAM66C | 0.51014122 | 3.60E-26 |
| RPLP0 | DANCR | 0.51620009 | 7.40E-27 |
| IL1B | SLC8A1-AS1 | 0.65134187 | 1.60E-46 |
| BCAT1 | SLC8A1-AS1 | 0.71672725 | 3.35E-60 |
| ABCC1 | SLC8A1-AS1 | 0.5080021 | 6.24E-26 |
| BDNF | SLC8A1-AS1 | 0.85753341 | 1.97E-109 |
| IL11 | SLC8A1-AS1 | 0.63947402 | 2.16E-44 |
| ANXA2 | SLC8A1-AS1 | 0.5225503 | 1.36E-27 |
| PKM | SLC8A1-AS1 | 0.58913518 | 2.56E-36 |
| STRA6 | SLC8A1-AS1 | 0.57965477 | 5.95E-35 |
| SIGLEC7 | ITGB2-AS1 | 0.56817832 | 2.34E-33 |
| CTLA4 | ITGB2-AS1 | 0.69444066 | 3.98E-55 |
| LTA | ITGB2-AS1 | 0.70096119 | 1.46E-56 |
| ACLY | LMCD1-AS1 | 0.52583026 | 5.60E-28 |
| SLC2A1 | SLC2A1-AS1 | 0.74692946 | 6.43E-68 |
| BCHE | SLC2A1-AS1 | 0.68127176 | 2.44E-52 |
| GAPDH | SLC2A1-AS1 | 0.51487212 | 1.05E-26 |
| MKI67 | SLC2A1-AS1 | 0.53497088 | 4.48E-29 |
| PLAU | AC018647.3 | 0.60676469 | 5.54E-39 |
| IL10 | AC018647.3 | 0.53418645 | 5.58E-29 |
| CXCL12 | AC018647.3 | 0.56384112 | 9.05E-33 |
| GJA1 | AC018647.3 | 0.50930773 | 4.46E-26 |
| PDGFRB | AC018647.3 | 0.5117462 | 2.37E-26 |
| DCN | AC018647.3 | 0.54603875 | 1.89E-30 |
| NES | AC018647.3 | 0.54210135 | 5.91E-30 |
| GLUL | LINC01132 | 0.53074444 | 1.45E-28 |
| SPP1 | SOX21-AS1 | 0.57776168 | 1.10E-34 |
| HAS3 | SOX21-AS1 | 0.57012923 | 1.27E-33 |
| RPLP2 | AC005884.1 | 0.57070511 | 1.06E-33 |
| IL1B | AC108463.2 | 0.57970644 | 5.85E-35 |
| FABP5 | AC108463.2 | 0.50597641 | 1.05E-25 |
| BCAT1 | AC108463.2 | 0.52704799 | 4.02E-28 |
| PLAU | AC108463.2 | 0.52783161 | 3.24E-28 |
| IL10 | AC108463.2 | 0.55522999 | 1.25E-31 |
| GLS | AC108463.2 | 0.50302002 | 2.22E-25 |
| ABCC1 | AC108463.2 | 0.64081635 | 1.25E-44 |
| BDNF | AC108463.2 | 0.55057398 | 5.00E-31 |
| BIRC5 | AC108463.2 | 0.51720872 | 5.67E-27 |
| GJA1 | AC108463.2 | 0.53296019 | 7.86E-29 |
| ANXA2 | AC108463.2 | 0.62313427 | 1.31E-41 |
| PKM | AC108463.2 | 0.63826638 | 3.51E-44 |
| BRCA1 | LINC01515 | 0.50475336 | 1.43E-25 |
| PRKCA | LINC01515 | 0.54009126 | 1.05E-29 |
| CAMK2G | LINC01515 | 0.53242842 | 9.11E-29 |
| PTGS2 | AC012368.1 | 0.54107102 | 7.94E-30 |
| MMP1 | AC012368.1 | 0.56902776 | 1.79E-33 |
| PLAU | RNF144A-AS1 | 0.5927221 | 7.57E-37 |
| IL10 | RNF144A-AS1 | 0.51849878 | 4.02E-27 |
| NES | RNF144A-AS1 | 0.686004 | 2.53E-53 |
| SPARC | HCG11 | 0.50653049 | 9.09E-26 |
| PTGS2 | HCG11 | 0.76402498 | 8.62E-73 |
| MMP1 | HCG11 | 0.74413023 | 3.71E-67 |
| FBN1 | HCG11 | 0.52987982 | 1.85E-28 |
| HSPG2 | HCG11 | 0.54582577 | 2.01E-30 |
| DCN | HCG11 | 0.57119189 | 9.05E-34 |
| KAT2A | LINC01004 | 0.50915864 | 4.63E-26 |
| GNRH1 | LINC01004 | 0.58731689 | 4.71E-36 |
| AKR1C1 | AP006222.2 | 0.70873382 | 2.51E-58 |
| TKT | AP006222.2 | 0.56892519 | 1.85E-33 |
| AKR1C3 | AP006222.2 | 0.5680527 | 2.44E-33 |
| LTA | LINC01013 | 0.61346133 | 4.87E-40 |
| SLC16A3 | CCDC183-AS1 | 0.50533311 | 1.23E-25 |
| FABP5 | HOTAIR | 0.51125519 | 2.70E-26 |
| PLAU | HOTAIR | 0.83697618 | 1.97E-99 |
| IL10 | HOTAIR | 0.73961693 | 5.95E-66 |
| BIRC5 | HOTAIR | 0.54777613 | 1.14E-30 |
| AGPAT4 | HOTAIR | 0.51798466 | 4.61E-27 |
| GJA1 | HOTAIR | 0.67384753 | 7.83E-51 |
| NES | HOTAIR | 0.65996084 | 3.96E-48 |
| MMP9 | AC079779.4 | 0.81677083 | 7.09E-91 |
| TGFB2 | AC079779.4 | 0.88620315 | 2.33E-126 |
| TFAP2A | AC079779.4 | 0.56139754 | 1.92E-32 |
| ACAN | AC079779.4 | 0.51842336 | 4.11E-27 |
| IBSP | AC079779.4 | 0.87285757 | 5.74E-118 |
| SLC22A12 | PCDH9-AS2 | 0.59423759 | 4.51E-37 |
| GLUL | LINC01344 | 0.54188182 | 6.30E-30 |
| LPAR2 | VIM-AS1 | 0.51215585 | 2.13E-26 |
| MMP9 | VIM-AS1 | 0.74764833 | 4.09E-68 |
| ABCC1 | VIM-AS1 | 0.59667511 | 1.94E-37 |
| ACE | VIM-AS1 | 0.53706872 | 2.48E-29 |
| TGFB2 | VIM-AS1 | 0.77635806 | 1.43E-76 |
| TFAP2A | VIM-AS1 | 0.64044444 | 1.46E-44 |
| ACAN | VIM-AS1 | 0.50101169 | 3.68E-25 |
| IBSP | VIM-AS1 | 0.70883382 | 2.38E-58 |
| FGFR3 | ANKRD10-IT1 | 0.53357623 | 6.62E-29 |
| TFAP2A | ANKRD10-IT1 | 0.5226102 | 1.34E-27 |
| GNRH1 | ANKRD10-IT1 | 0.58186582 | 2.88E-35 |
| SOX9 | ANKRD10-IT1 | 0.54986211 | 6.17E-31 |
| CTLA4 | LINC00582 | 0.52465726 | 7.71E-28 |
| RPLP2 | AC092155.1 | 0.59416528 | 4.62E-37 |
| SLC22A12 | SACS-AS1 | 0.50423715 | 1.63E-25 |
| IL10 | LINC01150 | 0.51714358 | 5.76E-27 |
| SIGLEC7 | LINC01150 | 0.65020863 | 2.59E-46 |
| CD4 | LINC01150 | 0.54489724 | 2.64E-30 |
| LGALS1 | LINC01150 | 0.55615657 | 9.45E-32 |
| PKM | LINC01150 | 0.54558311 | 2.16E-30 |
| MMP9 | AC013460.1 | 0.50726674 | 7.53E-26 |
| TFAP2A | AC013460.1 | 0.55998514 | 2.96E-32 |
| GNRH1 | UBE2Q1-AS1 | 0.54539817 | 2.28E-30 |
| PLAU | EMX2OS | 0.93805347 | 2.57E-173 |
| IL10 | EMX2OS | 0.89433896 | 5.25E-132 |
| BIRC5 | EMX2OS | 0.51348677 | 1.51E-26 |
| SLC38A5 | EMX2OS | 0.54785785 | 1.11E-30 |
| GJA1 | EMX2OS | 0.69407068 | 4.79E-55 |
| NES | EMX2OS | 0.67138686 | 2.42E-50 |
| CNR1 | AC007128.1 | 0.56104358 | 2.14E-32 |
| HMMR | ALMS1-IT1 | 0.52662147 | 4.52E-28 |
| G6PD | PRRT3-AS1 | 0.59186985 | 1.01E-36 |
| AKR1C1 | PRRT3-AS1 | 0.53993712 | 1.10E-29 |
| TKT | PRRT3-AS1 | 0.60357 | 1.73E-38 |
| MMP9 | TMEM254-AS1 | 0.60544159 | 8.90E-39 |
| TGFB2 | TMEM254-AS1 | 0.68208113 | 1.66E-52 |
| IBSP | TMEM254-AS1 | 0.6467816 | 1.08E-45 |
| FABP4 | HOXB-AS1 | 0.66118674 | 2.32E-48 |
| CETP | HOXB-AS1 | 0.60003301 | 6.03E-38 |
| LGALS1 | HOXB-AS1 | 0.53718714 | 2.40E-29 |
| PTHLH | LINC00443 | 0.84377275 | 1.41E-102 |
| IL1B | NFE4 | 0.59278929 | 7.40E-37 |
| BCAT1 | NFE4 | 0.54852326 | 9.15E-31 |
| BDNF | NFE4 | 0.64022362 | 1.59E-44 |
| ANXA2 | NFE4 | 0.5066314 | 8.86E-26 |
| GAP43 | NFE4 | 0.54655029 | 1.63E-30 |
| PTGS2 | XXYLT1-AS2 | 0.73089816 | 1.08E-63 |
| MMP1 | XXYLT1-AS2 | 0.8879364 | 1.59E-127 |
| MMP9 | AC009234.1 | 0.81879773 | 1.10E-91 |
| TGFB2 | AC009234.1 | 0.8492289 | 3.27E-105 |
| ACAN | AC009234.1 | 0.52930517 | 2.16E-28 |
| IBSP | AC009234.1 | 0.87594428 | 8.04E-120 |
| KAT2A | DSCR9 | 0.50519361 | 1.28E-25 |
| GLS | ZNF32-AS2 | 0.57319992 | 4.78E-34 |
| BRCA1 | ZNF32-AS2 | 0.51700631 | 5.98E-27 |
| FGFR3 | ZNF32-AS2 | 0.54459893 | 2.87E-30 |
| ABCC4 | ZNF32-AS2 | 0.51509861 | 9.88E-27 |
| NCOR2 | ZNF32-AS2 | 0.51650137 | 6.83E-27 |
| KAT2A | ZNF32-AS2 | 0.55264826 | 2.70E-31 |
| DNMT1 | ZNF32-AS2 | 0.51739044 | 5.40E-27 |
| TFAP2A | ZNF32-AS2 | 0.56350503 | 1.00E-32 |
| CAMK2G | ZNF32-AS2 | 0.60343764 | 1.82E-38 |
| GNRH1 | ZNF32-AS2 | 0.60125081 | 3.93E-38 |
| SOX9 | ZNF32-AS2 | 0.57761509 | 1.15E-34 |
| MMP9 | AC159540.1 | 0.58752393 | 4.40E-36 |
| GLS | AC159540.1 | 0.51448593 | 1.16E-26 |
| FGFR3 | AC159540.1 | 0.57460698 | 3.05E-34 |
| TGFB2 | AC159540.1 | 0.64781396 | 7.04E-46 |
| TFAP2A | AC159540.1 | 0.61324601 | 5.27E-40 |
| GNRH1 | AC159540.1 | 0.57806482 | 9.98E-35 |
| IBSP | AC159540.1 | 0.61952085 | 5.13E-41 |
| FABP4 | DNM3OS | 0.58408723 | 1.38E-35 |
| SPARC | DNM3OS | 0.6156877 | 2.14E-40 |
| CETP | DNM3OS | 0.57656924 | 1.62E-34 |
| IL10 | DNM3OS | 0.57694627 | 1.43E-34 |
| CXCL12 | DNM3OS | 0.60642058 | 6.27E-39 |
| HCAR1 | DNM3OS | 0.53779394 | 2.02E-29 |
| PDGFRB | DNM3OS | 0.59412834 | 4.68E-37 |
| TYRP1 | DNM3OS | 0.51979447 | 2.85E-27 |
| RNASE1 | DNM3OS | 0.50050495 | 4.17E-25 |
| DCN | DNM3OS | 0.59979403 | 6.56E-38 |
| HSPA4 | AC092171.4 | 0.50658311 | 8.97E-26 |
| LPAR2 | FOXD3-AS1 | 0.57006824 | 1.29E-33 |
| LOX | LINC01293 | 0.50050948 | 4.17E-25 |
| CDK4 | ZNF674-AS1 | 0.53472978 | 4.79E-29 |
| CS | HCG18 | 0.58334158 | 1.77E-35 |
| NCOR2 | HCG18 | 0.51090446 | 2.95E-26 |
| CDK4 | HCG18 | 0.58121111 | 3.57E-35 |
| NPM1 | HCG18 | 0.51876102 | 3.75E-27 |
| EHMT2 | HCG18 | 0.78373788 | 5.96E-79 |
| DNMT1 | HCG18 | 0.50799674 | 6.25E-26 |
| CAMK2G | HCG18 | 0.52278055 | 1.28E-27 |
| TUBB | HCG18 | 0.66352943 | 8.26E-49 |
| ACLY | HCG18 | 0.53595867 | 3.39E-29 |
| DVL2 | HCG18 | 0.50029591 | 4.40E-25 |
| SPP1 | LINC01436 | 0.58121349 | 3.57E-35 |
| SLC22A12 | LINC01508 | 0.6459121 | 1.55E-45 |
| G6PD | LINC01508 | 0.51721715 | 5.65E-27 |
| TKT | LINC01508 | 0.56111337 | 2.09E-32 |
| SLC6A3 | AC007099.1 | 0.6432812 | 4.59E-45 |
| HSPB1 | FARP1-AS1 | 0.57630269 | 1.77E-34 |
| MMP1 | TRIM31-AS1 | 0.5849827 | 1.03E-35 |
| TNFRSF11A | TRIM31-AS1 | 0.50580858 | 1.09E-25 |
| BCAT1 | FAM225A | 0.52802391 | 3.08E-28 |
| BDNF | FAM225A | 0.55271162 | 2.65E-31 |
| IL11 | FAM225A | 0.56331028 | 1.07E-32 |
| LGALS1 | FAM225A | 0.61874241 | 6.87E-41 |
| PKM | FAM225A | 0.58506684 | 9.99E-36 |
| STRA6 | FAM225A | 0.63749917 | 4.78E-44 |
| DNMT1 | DLEU2 | 0.53953267 | 1.23E-29 |
| CDK1 | DLEU2 | 0.57307512 | 4.97E-34 |
| CNR1 | FSIP2-AS1 | 0.53877566 | 1.53E-29 |
| PPARG | DIRC3 | 0.50154365 | 3.22E-25 |
| FABP4 | DIRC3 | 0.64342699 | 4.32E-45 |
| CETP | DIRC3 | 0.55694195 | 7.45E-32 |
| PLG | LINC00574 | 0.54243109 | 5.38E-30 |
| HSPB1 | AC079922.2 | 0.5082841 | 5.80E-26 |
| RPLP2 | AC079922.2 | 0.65399999 | 5.19E-47 |
| CSNK2B | AC079922.2 | 0.50486525 | 1.39E-25 |
| SLC16A3 | PIK3CD-AS2 | 0.53432119 | 5.37E-29 |
| FABP5 | LINC01198 | 0.51600289 | 7.79E-27 |
| PLAU | LINC01198 | 0.94502553 | 1.12E-182 |
| IL10 | LINC01198 | 0.82904622 | 6.12E-96 |
| BIRC5 | LINC01198 | 0.56494115 | 6.43E-33 |
| SLC38A5 | LINC01198 | 0.57813114 | 9.77E-35 |
| GJA1 | LINC01198 | 0.70393593 | 3.13E-57 |
| NES | LINC01198 | 0.68499994 | 4.10E-53 |
| BRCA1 | TRAF3IP2-AS1 | 0.55287168 | 2.53E-31 |
| CDKN3 | TRAF3IP2-AS1 | 0.50764524 | 6.84E-26 |
| PARP1 | TRAF3IP2-AS1 | 0.52616226 | 5.12E-28 |
| NCOR2 | TRAF3IP2-AS1 | 0.6065164 | 6.06E-39 |
| BIRC5 | TRAF3IP2-AS1 | 0.50051845 | 4.16E-25 |
| TYMS | TRAF3IP2-AS1 | 0.61572304 | 2.11E-40 |
| CDK4 | TRAF3IP2-AS1 | 0.65378821 | 5.68E-47 |
| EHMT2 | TRAF3IP2-AS1 | 0.51676167 | 6.38E-27 |
| PCNA | TRAF3IP2-AS1 | 0.56182774 | 1.68E-32 |
| DNMT1 | TRAF3IP2-AS1 | 0.65606179 | 2.15E-47 |
| CDK1 | TRAF3IP2-AS1 | 0.57184001 | 7.37E-34 |
| PLCG1 | TRAF3IP2-AS1 | 0.60930129 | 2.22E-39 |
| PRKCD | TRAF3IP2-AS1 | 0.57361118 | 4.19E-34 |
| TUBB | TRAF3IP2-AS1 | 0.51352971 | 1.49E-26 |
| ACLY | TRAF3IP2-AS1 | 0.55137767 | 3.94E-31 |
| EZH2 | TRAF3IP2-AS1 | 0.52992391 | 1.82E-28 |
| DVL2 | TRAF3IP2-AS1 | 0.57887482 | 7.67E-35 |
| CNR1 | AC007682.1 | 0.53906124 | 1.41E-29 |
| LPAR2 | NEBL-AS1 | 0.63613869 | 8.24E-44 |
| PFKM | NEBL-AS1 | 0.51730162 | 5.53E-27 |
| CNR1 | AC005537.2 | 0.53802076 | 1.89E-29 |
| TH | AL132709.8 | 0.559959 | 2.98E-32 |
| BCAT1 | AL132709.8 | 0.51366536 | 1.44E-26 |
| CHGA | AL132709.8 | 0.74910733 | 1.62E-68 |
| PLAU | LEF1-AS1 | 0.73541553 | 7.50E-65 |
| IL10 | LEF1-AS1 | 0.62678731 | 3.23E-42 |
| SLC38A5 | LEF1-AS1 | 0.51591506 | 7.97E-27 |
| GJA1 | LEF1-AS1 | 0.58572901 | 8.02E-36 |
| NES | LEF1-AS1 | 0.62158218 | 2.36E-41 |
| GAP43 | AC009410.1 | 0.52063828 | 2.27E-27 |
| PLAU | RFX3-AS1 | 0.84284331 | 3.88E-102 |
| IL10 | RFX3-AS1 | 0.74606818 | 1.11E-67 |
| BIRC5 | RFX3-AS1 | 0.53374374 | 6.32E-29 |
| GJA1 | RFX3-AS1 | 0.65352963 | 6.34E-47 |
| NES | RFX3-AS1 | 0.61424839 | 3.65E-40 |
| HBB | NCOA7-AS1 | 0.52198542 | 1.59E-27 |
| FASN | SLC6A1-AS1 | 0.52036951 | 2.44E-27 |
| PTGS2 | VIPR1-AS1 | 0.52407517 | 9.02E-28 |
| MMP1 | VIPR1-AS1 | 0.54434158 | 3.10E-30 |
| MMP9 | TGFB2-AS1 | 0.80137264 | 4.89E-85 |
| ABCC1 | TGFB2-AS1 | 0.51838216 | 4.15E-27 |
| TGFB2 | TGFB2-AS1 | 0.93714376 | 3.54E-172 |
| TFAP2A | TGFB2-AS1 | 0.54348771 | 3.96E-30 |
| ACAN | TGFB2-AS1 | 0.56426714 | 7.93E-33 |
| IBSP | TGFB2-AS1 | 0.8425532 | 5.31E-102 |
| GLUL | LINC01549 | 0.54066503 | 8.92E-30 |
| BMP4 | LINC01549 | 0.53526757 | 4.12E-29 |
| FABP4 | IDI2-AS1 | 0.59087476 | 1.42E-36 |
| CETP | IDI2-AS1 | 0.56278957 | 1.25E-32 |
| HCAR1 | IDI2-AS1 | 0.67449257 | 5.82E-51 |
| TYRP1 | IDI2-AS1 | 0.71251724 | 3.31E-59 |
| RNASE1 | IDI2-AS1 | 0.58557373 | 8.44E-36 |
| CDK4 | LINC00665 | 0.53229901 | 9.45E-29 |
| EHMT2 | LINC00665 | 0.54225113 | 5.66E-30 |
| LPAR2 | LINC00342 | 0.53359711 | 6.58E-29 |
| GLS | LINC00342 | 0.51707473 | 5.87E-27 |
| ABCC1 | LINC00342 | 0.5109951 | 2.88E-26 |
| TGFB2 | LINC00342 | 0.54457826 | 2.89E-30 |
| TFAP2A | LINC00342 | 0.67334048 | 9.89E-51 |
| GNRH1 | LINC00342 | 0.69279869 | 9.03E-55 |
| CYP1A2 | CYP1B1-AS1 | 0.52623067 | 5.02E-28 |
| CHGA | LINC01219 | 0.55471161 | 1.46E-31 |
| RPLP0 | SNHG7 | 0.59168471 | 1.08E-36 |
| RPLP2 | SNHG7 | 0.5511521 | 4.21E-31 |
| LTA | LINC00892 | 0.72494539 | 3.37E-62 |
| FABP4 | LINC00702 | 0.52784387 | 3.23E-28 |
| SPARC | LINC00702 | 0.7059576 | 1.09E-57 |
| CETP | LINC00702 | 0.50493619 | 1.37E-25 |
| FBN1 | LINC00702 | 0.58182042 | 2.92E-35 |
| HCAR1 | LINC00702 | 0.5451361 | 2.46E-30 |
| COL1A1 | LINC00702 | 0.51957331 | 3.02E-27 |
| PDGFRB | LINC00702 | 0.57932376 | 6.62E-35 |
| TYRP1 | LINC00702 | 0.60149374 | 3.61E-38 |
| RNASE1 | LINC00702 | 0.56760023 | 2.81E-33 |
| MYCN | AC002511.2 | 0.56528354 | 5.79E-33 |
| TFAP2A | LINC00472 | 0.52402538 | 9.14E-28 |
| GNRH1 | LINC00472 | 0.53047543 | 1.57E-28 |
| ITGA2 | LINC00472 | 0.59077406 | 1.47E-36 |
| BCAT1 | TLR8-AS1 | 0.61717847 | 1.23E-40 |
| BDNF | TLR8-AS1 | 0.90026136 | 2.04E-136 |
| IL11 | TLR8-AS1 | 0.99790221 | 0 |
| LGALS1 | TLR8-AS1 | 0.6140525 | 3.92E-40 |
| PKM | TLR8-AS1 | 0.55166284 | 3.62E-31 |
| STRA6 | TLR8-AS1 | 0.84490122 | 4.10E-103 |
| CTLA4 | CHRM3-AS2 | 0.55521379 | 1.25E-31 |
| LTA | CHRM3-AS2 | 0.70462536 | 2.19E-57 |
| FABP4 | HOTAIRM1 | 0.54264015 | 5.06E-30 |
| LGALS1 | HOTAIRM1 | 0.62633063 | 3.85E-42 |
| AGPAT4 | ZNF197-AS1 | 0.5251985 | 6.65E-28 |
| CDK4 | ZNF529-AS1 | 0.5171069 | 5.82E-27 |
| EHMT2 | ZNF529-AS1 | 0.53533918 | 4.04E-29 |
| RPLP0 | ZNF529-AS1 | 0.54163676 | 6.75E-30 |
| TUBB | ZNF529-AS1 | 0.5027681 | 2.36E-25 |
| RPLP2 | ZNF529-AS1 | 0.53060983 | 1.51E-28 |
| PLAU | LINC00460 | 0.7488803 | 1.87E-68 |
| IL10 | LINC00460 | 0.65605217 | 2.15E-47 |
| GJA1 | LINC00460 | 0.60197267 | 3.05E-38 |
| NES | LINC00460 | 0.58846075 | 3.21E-36 |
| HCAR1 | SLC26A4-AS1 | 0.78819047 | 1.97E-80 |
| TYRP1 | SLC26A4-AS1 | 0.63956675 | 2.08E-44 |
| RNASE1 | SLC26A4-AS1 | 0.55502308 | 1.33E-31 |
| MYCN | MYCNOS | 0.82194293 | 5.81E-93 |
| SLC22A12 | LINC01122 | 0.53846833 | 1.67E-29 |
| FFAR4 | LINC01136 | 0.53071992 | 1.46E-28 |
| IL4I1 | AC026904.1 | 0.61480007 | 2.97E-40 |
| NCOR2 | YEATS2-AS1 | 0.5951724 | 3.27E-37 |
| DNMT1 | YEATS2-AS1 | 0.61505682 | 2.71E-40 |
| MKI67 | YEATS2-AS1 | 0.50972148 | 4.01E-26 |
| CAMK2G | YEATS2-AS1 | 0.54232189 | 5.55E-30 |
| GNRH1 | YEATS2-AS1 | 0.58704759 | 5.16E-36 |
| CYP3A4 | TPRG1-AS1 | 0.6253069 | 5.70E-42 |
| GLUL | TPRG1-AS1 | 0.67129925 | 2.52E-50 |
| CDK4 | RNASEH1-AS1 | 0.51803702 | 4.55E-27 |
| MMP9 | LINC01191 | 0.52252308 | 1.37E-27 |
| TGFB2 | LINC01191 | 0.56257251 | 1.34E-32 |
| TFAP2A | LINC01191 | 0.55325223 | 2.26E-31 |
| IBSP | LINC01191 | 0.50557703 | 1.16E-25 |
| AGPAT4 | DEPDC1-AS1 | 0.52367619 | 1.00E-27 |
| RPLP0 | AP000936.1 | 0.52069314 | 2.24E-27 |
| RPLP2 | AP000936.1 | 0.50882278 | 5.05E-26 |
| MAPK3 | MAPKAPK5-AS1 | 0.51282034 | 1.79E-26 |
| BAX | MAPKAPK5-AS1 | 0.59467965 | 3.87E-37 |
| BIRC5 | MAPKAPK5-AS1 | 0.51924487 | 3.30E-27 |
| HRAS | MAPKAPK5-AS1 | 0.69084528 | 2.38E-54 |
| KAT2A | MAPKAPK5-AS1 | 0.50105618 | 3.64E-25 |
| CDK4 | MAPKAPK5-AS1 | 0.54362452 | 3.81E-30 |
| NPM1 | MAPKAPK5-AS1 | 0.50986293 | 3.86E-26 |
| EHMT2 | MAPKAPK5-AS1 | 0.55925909 | 3.69E-32 |
| GAPDH | MAPKAPK5-AS1 | 0.53676356 | 2.70E-29 |
| RPLP0 | MAPKAPK5-AS1 | 0.62087466 | 3.08E-41 |
| HSPB1 | MAPKAPK5-AS1 | 0.51260854 | 1.90E-26 |
| RPLP2 | MAPKAPK5-AS1 | 0.61542818 | 2.36E-40 |
| CSNK2B | MAPKAPK5-AS1 | 0.58739472 | 4.59E-36 |
| BGLAP | GAS5 | 0.50677933 | 8.53E-26 |
| KAT2A | GAS5 | 0.50231654 | 2.65E-25 |
| NPM1 | GAS5 | 0.57638166 | 1.72E-34 |
| RPLP0 | GAS5 | 0.6940041 | 4.95E-55 |
| FGFR4 | GAS5 | 0.51814724 | 4.42E-27 |
| RPLP2 | GAS5 | 0.57725474 | 1.30E-34 |
| RPLP0 | FOXP4-AS1 | 0.50826555 | 5.83E-26 |
| RPLP2 | FOXP4-AS1 | 0.61955695 | 5.06E-41 |
| MMP3 | LINC00676 | 0.57943196 | 6.39E-35 |
| CYP19A1 | LINC00163 | 0.68549392 | 3.23E-53 |
| IL1B | MIR155HG | 0.55169206 | 3.59E-31 |
| BCAT1 | MIR155HG | 0.56400404 | 8.60E-33 |
| SIGLEC7 | MIR155HG | 0.66806566 | 1.09E-49 |
| BDNF | MIR155HG | 0.69237613 | 1.11E-54 |
| CD4 | MIR155HG | 0.56840209 | 2.18E-33 |
| CTLA4 | MIR155HG | 0.71424288 | 1.30E-59 |
| IL11 | MIR155HG | 0.66289576 | 1.09E-48 |
| CCL3 | MIR155HG | 0.57851242 | 8.63E-35 |
| PKM | MIR155HG | 0.51642958 | 6.96E-27 |
| STRA6 | MIR155HG | 0.6410753 | 1.13E-44 |
| LTA | MIR155HG | 0.74785472 | 3.59E-68 |
| BIRC5 | SNHG20 | 0.53166297 | 1.13E-28 |
| KAT2A | SNHG20 | 0.63324031 | 2.60E-43 |
| CDK4 | SNHG20 | 0.51936763 | 3.19E-27 |
| ASNS | SNHG20 | 0.50115612 | 3.55E-25 |
| KAT2A | SEMA3F-AS1 | 0.50818854 | 5.95E-26 |
| GNRH1 | SEMA3F-AS1 | 0.50259216 | 2.47E-25 |
| PLAU | LINC00601 | 0.73872465 | 1.02E-65 |
| IL10 | LINC00601 | 0.63762356 | 4.55E-44 |
| GJA1 | LINC00601 | 0.56951131 | 1.54E-33 |
| NES | LINC00601 | 0.54783005 | 1.12E-30 |
| SLC6A3 | NUCB1-AS1 | 0.58690987 | 5.40E-36 |
| CTLA4 | LINC01281 | 0.53312744 | 7.50E-29 |
| LTA | LINC01281 | 0.86332617 | 1.55E-112 |
| CS | LINC00957 | 0.50958478 | 4.15E-26 |
| BRCA1 | LINC01278 | 0.50628232 | 9.69E-26 |
| PARP1 | LINC01278 | 0.50642119 | 9.35E-26 |
| NCOR2 | LINC01278 | 0.50314184 | 2.15E-25 |
| CAMK2G | LINC01278 | 0.53975831 | 1.16E-29 |
| IDO1 | AC092580.4 | 0.51163562 | 2.44E-26 |
| CTLA4 | AC092580.4 | 0.77486623 | 4.22E-76 |
| CCL3 | AC092580.4 | 0.56809697 | 2.40E-33 |
| SOX9 | LINC00494 | 0.51691967 | 6.12E-27 |
| FABP4 | LINC00484 | 0.61674611 | 1.45E-40 |
| CETP | LINC00484 | 0.56762561 | 2.79E-33 |
| CNR1 | AC023347.1 | 0.50660215 | 8.93E-26 |
| GLUL | SRGAP3-AS4 | 0.57922704 | 6.84E-35 |
| MMP3 | BHLHE40-AS1 | 0.67468335 | 5.33E-51 |
| GNRH1 | LINC00941 | 0.53275574 | 8.32E-29 |
| G6PD | TM4SF19-AS1 | 0.5976443 | 1.39E-37 |
| EHMT2 | ASH1L-AS1 | 0.52354343 | 1.04E-27 |
| GNRH1 | ASH1L-AS1 | 0.51832429 | 4.22E-27 |
| LGALS1 | NEXN-AS1 | 0.50047663 | 4.20E-25 |
| GNRH1 | NEXN-AS1 | 0.59590005 | 2.54E-37 |
| PKM | NEXN-AS1 | 0.59472289 | 3.81E-37 |
| GNRH1 | UBOX5-AS1 | 0.56021715 | 2.76E-32 |
| ACVRL1 | AC109642.1 | 0.51556779 | 8.74E-27 |
| DNAH8 | AC019117.2 | 0.57986125 | 5.56E-35 |
| CXCR2 | AC019117.2 | 0.50674986 | 8.60E-26 |
| ABCC1 | LINC01447 | 0.55181739 | 3.46E-31 |
| ALOX12 | COX10-AS1 | 0.56026563 | 2.72E-32 |
| DVL2 | COX10-AS1 | 0.51280415 | 1.80E-26 |
| ALOX12 | TMEM147-AS1 | 0.51856982 | 3.95E-27 |
| CDK4 | TMEM147-AS1 | 0.50261487 | 2.46E-25 |
| BRCA1 | KDM4A-AS1 | 0.55387995 | 1.87E-31 |
| HMMR | KDM4A-AS1 | 0.59914946 | 8.22E-38 |
| ALOX12 | LINC01376 | 0.50582467 | 1.09E-25 |
| LPL | C10orf71-AS1 | 0.51482702 | 1.06E-26 |
| CYP19A1 | C10orf71-AS1 | 0.57017144 | 1.25E-33 |
| LTA | AC006369.2 | 0.67053506 | 3.57E-50 |
| AKR1C1 | DIAPH2-AS1 | 0.55166132 | 3.62E-31 |
| FABP4 | LINC01361 | 0.73299813 | 3.15E-64 |
| CETP | LINC01361 | 0.74057528 | 3.32E-66 |
| MAPT | LINC01361 | 0.5492765 | 7.33E-31 |
| HCAR1 | LINC01361 | 0.84697208 | 4.14E-104 |
| TYRP1 | LINC01361 | 0.94552207 | 2.18E-183 |
| RNASE1 | LINC01361 | 0.79657905 | 2.53E-83 |
| SPARC | AP001189.4 | 0.58595419 | 7.44E-36 |
| FBN1 | AP001189.4 | 0.62587859 | 4.58E-42 |
| HSPG2 | AP001189.4 | 0.65669449 | 1.63E-47 |
| PDGFRB | AP001189.4 | 0.53178197 | 1.09E-28 |
| NGFR | AP001189.4 | 0.56491588 | 6.48E-33 |
| DCN | AP001189.4 | 0.55100524 | 4.40E-31 |
| FABP5 | VLDLR-AS1 | 0.54117926 | 7.70E-30 |
| PLAU | VLDLR-AS1 | 0.87580647 | 9.75E-120 |
| IL10 | VLDLR-AS1 | 0.80158807 | 4.09E-85 |
| BIRC5 | VLDLR-AS1 | 0.58225043 | 2.54E-35 |
| SLC38A5 | VLDLR-AS1 | 0.52746769 | 3.58E-28 |
| GJA1 | VLDLR-AS1 | 0.64882676 | 4.62E-46 |
| NES | VLDLR-AS1 | 0.63053725 | 7.53E-43 |
| BCAT1 | AC003092.1 | 0.63307448 | 2.78E-43 |
| BDNF | AC003092.1 | 0.90357766 | 5.22E-139 |
| IL11 | AC003092.1 | 0.99772453 | 0 |
| LGALS1 | AC003092.1 | 0.62125719 | 2.67E-41 |
| PKM | AC003092.1 | 0.56423957 | 8.00E-33 |
| STRA6 | AC003092.1 | 0.84580814 | 1.51E-103 |
| CTLA4 | AC002331.1 | 0.57054448 | 1.11E-33 |
| KAT2A | AC107081.5 | 0.56015649 | 2.81E-32 |
| GNRH1 | AC107081.5 | 0.54229323 | 5.59E-30 |
| SLC7A11 | LINC01231 | 0.5052132 | 1.27E-25 |
| KAT2A | LINC01424 | 0.52110615 | 2.01E-27 |
| GLUL | RNF217-AS1 | 0.52419775 | 8.73E-28 |
| BAX | PITPNA-AS1 | 0.53110684 | 1.32E-28 |
| RPLP0 | PITPNA-AS1 | 0.54056912 | 9.17E-30 |
| FGFR4 | PITPNA-AS1 | 0.54207535 | 5.96E-30 |
| DVL2 | PITPNA-AS1 | 0.62642699 | 3.71E-42 |
| SLC22A12 | OVAAL | 0.82408969 | 7.56E-94 |
| GLUL | LINC01186 | 0.53133083 | 1.24E-28 |
| SHC1 | MKLN1-AS | 0.54795898 | 1.08E-30 |
| NPM1 | MKLN1-AS | 0.54836616 | 9.58E-31 |
| HSPA4 | MKLN1-AS | 0.50881298 | 5.07E-26 |
| ACLY | MKLN1-AS | 0.51889458 | 3.62E-27 |
| AKR1C1 | LINC01474 | 0.6272845 | 2.66E-42 |
| MAPK3 | NIFK-AS1 | 0.52511986 | 6.80E-28 |
| EHMT2 | NIFK-AS1 | 0.5438039 | 3.62E-30 |
| TUBB | NIFK-AS1 | 0.51001881 | 3.71E-26 |
| FGFR3 | MIR600HG | 0.61302202 | 5.72E-40 |
| CTLA4 | AP003774.1 | 0.69063904 | 2.63E-54 |
| LTA | AP003774.1 | 0.68013172 | 4.18E-52 |
| NCOR2 | ZDHHC20-IT1 | 0.50784027 | 6.50E-26 |
| ALOX12 | ZEB1-AS1 | 0.54050729 | 9.33E-30 |
| SLC22A5 | ZEB1-AS1 | 0.51931312 | 3.24E-27 |
| CS | ZEB1-AS1 | 0.55939539 | 3.54E-32 |
| KAT2A | PRMT5-AS1 | 0.53049651 | 1.56E-28 |
| FABP4 | HAND2-AS1 | 0.7732615 | 1.34E-75 |
| CETP | HAND2-AS1 | 0.78177591 | 2.61E-78 |
| HCAR1 | HAND2-AS1 | 0.79057214 | 3.07E-81 |
| TYRP1 | HAND2-AS1 | 0.84011977 | 7.22E-101 |
| RNASE1 | HAND2-AS1 | 0.75578559 | 2.16E-70 |
| PTK2 | AC073254.1 | 0.51097653 | 2.90E-26 |
| SIGLEC7 | TMEM26-AS1 | 0.644874 | 2.38E-45 |
| CCL3 | TMEM26-AS1 | 0.50275064 | 2.37E-25 |
| FFAR4 | LINC00987 | 0.6947147 | 3.47E-55 |
| CYP3A4 | TUSC8 | 0.53536923 | 4.00E-29 |
| GLUL | TUSC8 | 0.63234137 | 3.71E-43 |
| BMP4 | TUSC8 | 0.50341523 | 2.01E-25 |
| ATP6V1B1 | HOXD-AS2 | 0.55035017 | 5.34E-31 |
| SLC22A5 | PITRM1-AS1 | 0.53588511 | 3.46E-29 |
| CS | PITRM1-AS1 | 0.51880754 | 3.71E-27 |
| PFKM | PITRM1-AS1 | 0.57800288 | 1.02E-34 |
| BIRC5 | FOXD2-AS1 | 0.50107364 | 3.62E-25 |
| CNR1 | LINC00383 | 0.54083387 | 8.50E-30 |
| FABP5 | TTLL11-IT1 | 0.58667916 | 5.84E-36 |
| PLAU | TTLL11-IT1 | 0.8234188 | 1.43E-93 |
| IL10 | TTLL11-IT1 | 0.71744501 | 2.25E-60 |
| BIRC5 | TTLL11-IT1 | 0.61507034 | 2.69E-40 |
| SLC38A5 | TTLL11-IT1 | 0.58042455 | 4.62E-35 |
| GJA1 | TTLL11-IT1 | 0.65120239 | 1.70E-46 |
| NES | TTLL11-IT1 | 0.72154237 | 2.31E-61 |
| FABP4 | AC010980.2 | 0.73464514 | 1.19E-64 |
| CETP | AC010980.2 | 0.69781637 | 7.27E-56 |
| MAPT | AC010980.2 | 0.51395584 | 1.33E-26 |
| HCAR1 | AC010980.2 | 0.69661176 | 1.34E-55 |
| TYRP1 | AC010980.2 | 0.73495096 | 9.89E-65 |
| RNASE1 | AC010980.2 | 0.69096619 | 2.24E-54 |
| FFAR4 | LINC00322 | 0.82506444 | 2.97E-94 |
| IL1B | AC073130.1 | 0.56519642 | 5.94E-33 |
| BCAT1 | AC073130.1 | 0.63365405 | 2.21E-43 |
| BDNF | AC073130.1 | 0.77787225 | 4.72E-77 |
| IL11 | AC073130.1 | 0.62233582 | 1.77E-41 |
| LGALS1 | AC073130.1 | 0.53649444 | 2.92E-29 |
| PKM | AC073130.1 | 0.53530834 | 4.07E-29 |
| STRA6 | AC073130.1 | 0.55823414 | 5.04E-32 |
| KAT2A | DGUOK-AS1 | 0.53390551 | 6.04E-29 |
| GNRH1 | DGUOK-AS1 | 0.52378981 | 9.75E-28 |
| PPARG | AC092839.1 | 0.51250937 | 1.95E-26 |
| LPL | AC092839.1 | 0.51185162 | 2.31E-26 |
| NES | NFIA-AS2 | 0.54484377 | 2.68E-30 |
| FABP5 | PRKCQ-AS1 | 0.54083013 | 8.51E-30 |
| PLAU | PRKCQ-AS1 | 0.86748838 | 7.43E-115 |
| IL10 | PRKCQ-AS1 | 0.82068471 | 1.90E-92 |
| BIRC5 | PRKCQ-AS1 | 0.52830789 | 2.85E-28 |
| SLC38A5 | PRKCQ-AS1 | 0.553869 | 1.88E-31 |
| GJA1 | PRKCQ-AS1 | 0.70486641 | 1.93E-57 |
| NES | PRKCQ-AS1 | 0.68739971 | 1.28E-53 |
| CYP3A4 | LINC00844 | 0.57652563 | 1.64E-34 |
| FABP4 | ZEB2-AS1 | 0.61918592 | 5.81E-41 |
| CETP | ZEB2-AS1 | 0.61624115 | 1.74E-40 |
| LGALS1 | ZEB2-AS1 | 0.51490899 | 1.04E-26 |
| IDO1 | LINC00426 | 0.52887057 | 2.44E-28 |
| SIGLEC7 | LINC00426 | 0.56951134 | 1.54E-33 |
| CD4 | LINC00426 | 0.54034131 | 9.79E-30 |
| CTLA4 | LINC00426 | 0.66751973 | 1.39E-49 |
| LTA | LINC00426 | 0.85912608 | 2.86E-110 |
| ACACA | MID1IP1-AS1 | 0.51188472 | 2.29E-26 |
| PRKCA | MID1IP1-AS1 | 0.52094867 | 2.09E-27 |
| BIRC5 | MYLK-AS1 | 0.51435606 | 1.20E-26 |
| RPLP2 | MYLK-AS1 | 0.51818174 | 4.38E-27 |
| PTGS2 | HOXB-AS2 | 0.69647025 | 1.44E-55 |
| MMP1 | HOXB-AS2 | 0.77342775 | 1.19E-75 |
| TFAP2A | HOXB-AS2 | 0.5288921 | 2.42E-28 |
| ITGA2 | HOXB-AS2 | 0.5123657 | 2.02E-26 |
| CNR1 | LINC00879 | 0.77731891 | 7.09E-77 |
| CAMK2G | PSMD6-AS2 | 0.51776222 | 4.89E-27 |
| ABCC1 | AC009120.3 | 0.5216529 | 1.73E-27 |
| GNRH1 | AC009120.3 | 0.54011313 | 1.04E-29 |
| FABP5 | ERICH6-AS1 | 0.5212832 | 1.91E-27 |
| PLAU | ERICH6-AS1 | 0.52127147 | 1.92E-27 |
| IL10 | ERICH6-AS1 | 0.51815314 | 4.41E-27 |
| BIRC5 | ERICH6-AS1 | 0.58686084 | 5.49E-36 |
| IL1B | AC017002.1 | 0.51235813 | 2.02E-26 |
| ABCC1 | AC017002.1 | 0.56726819 | 3.12E-33 |
| CTLA4 | AC017002.1 | 0.61029783 | 1.55E-39 |
| CDKN2A | CDKN2B-AS1 | 0.73593977 | 5.48E-65 |
| PCNA | CDKN2B-AS1 | 0.52712825 | 3.93E-28 |
| CYP1A2 | LINC00886 | 0.69906896 | 3.84E-56 |
| ALDH3A1 | LINC00886 | 0.67882052 | 7.75E-52 |
| CS | NDUFB2-AS1 | 0.54626575 | 1.77E-30 |
| CDK4 | NDUFB2-AS1 | 0.54819129 | 1.01E-30 |
| NPM1 | NDUFB2-AS1 | 0.53662226 | 2.81E-29 |
| LPAR2 | ARHGAP31-AS1 | 0.52452066 | 8.00E-28 |
| ABCC1 | ARHGAP31-AS1 | 0.5111508 | 2.77E-26 |
| ABCC4 | ARHGAP31-AS1 | 0.53342202 | 6.91E-29 |
| TGFB2 | ARHGAP31-AS1 | 0.55345687 | 2.12E-31 |
| TFAP2A | ARHGAP31-AS1 | 0.52252991 | 1.37E-27 |
| SPARC | ADAMTS9-AS1 | 0.5239811 | 9.25E-28 |
| CXCL12 | ADAMTS9-AS1 | 0.55400125 | 1.80E-31 |
| FBN1 | ADAMTS9-AS1 | 0.58490579 | 1.05E-35 |
| HSPG2 | ADAMTS9-AS1 | 0.54300712 | 4.55E-30 |
| COL1A1 | ADAMTS9-AS1 | 0.53385058 | 6.13E-29 |
| NGFR | ADAMTS9-AS1 | 0.57202193 | 6.96E-34 |
| DCN | ADAMTS9-AS1 | 0.6361875 | 8.08E-44 |
| CTLA4 | ZBTB20-AS1 | 0.56942825 | 1.58E-33 |
| LTA | TRBV11-2 | 0.73169883 | 6.77E-64 |
| PFKM | ADAMTS9-AS2 | 0.54145752 | 7.11E-30 |
| IL4I1 | LINC01324 | 0.52060734 | 2.29E-27 |
| PLAU | LINC01324 | 0.51134187 | 2.64E-26 |
| LMNA | PRR34-AS1 | 0.5004241 | 4.26E-25 |
| HRAS | PRR34-AS1 | 0.52075661 | 2.20E-27 |
| RPLP2 | PRR34-AS1 | 0.53046894 | 1.57E-28 |
| FABP5 | SNHG3 | 0.52277157 | 1.28E-27 |
| BCAT1 | SNHG3 | 0.5335869 | 6.60E-29 |
| RPLP0 | SNHG3 | 0.51705815 | 5.90E-27 |
| IDO1 | LINC00996 | 0.64738994 | 8.41E-46 |
| SIGLEC7 | LINC00996 | 0.5073268 | 7.42E-26 |
| CTLA4 | LINC00996 | 0.54353385 | 3.91E-30 |
| LTA | LINC00996 | 0.79748133 | 1.21E-83 |
| PLAU | SOX2-OT | 0.95468435 | 6.90E-198 |
| IL10 | SOX2-OT | 0.82221818 | 4.48E-93 |
| BIRC5 | SOX2-OT | 0.53765323 | 2.10E-29 |
| SLC38A5 | SOX2-OT | 0.55796 | 5.48E-32 |
| GJA1 | SOX2-OT | 0.702805 | 5.63E-57 |
| NES | SOX2-OT | 0.67817992 | 1.05E-51 |
| ALOX12 | MCCC1-AS1 | 0.507313 | 7.45E-26 |
| FGFR3 | MCCC1-AS1 | 0.53089199 | 1.40E-28 |
| PFKM | MCCC1-AS1 | 0.56489779 | 6.52E-33 |
| GNRH1 | MCCC1-AS1 | 0.50720172 | 7.66E-26 |
| FASN | LINC00880 | 0.51059683 | 3.20E-26 |
| FABP4 | WDR86-AS1 | 0.5036703 | 1.88E-25 |
| GLS | LINC01011 | 0.50896602 | 4.87E-26 |
| MAPK3 | LINC01011 | 0.51119035 | 2.74E-26 |
| EHMT2 | LINC01011 | 0.54543062 | 2.26E-30 |
| ANXA2 | LINC01011 | 0.53852579 | 1.64E-29 |
| PKM | LINC01011 | 0.51151047 | 2.52E-26 |
| SLC6A3 | FLNB-AS1 | 0.5080847 | 6.11E-26 |
| CYP3A4 | SIAH2-AS1 | 0.56471019 | 6.91E-33 |
| IL1B | AC108676.1 | 0.51371948 | 1.42E-26 |
| ABCC1 | AC108676.1 | 0.57096142 | 9.74E-34 |
| NCOR2 | ALKBH3-AS1 | 0.53057375 | 1.52E-28 |
| ABCC1 | LIFR-AS1 | 0.51929896 | 3.25E-27 |
| CS | LINC00847 | 0.54843534 | 9.39E-31 |
| FABP4 | IGFBP7-AS1 | 0.59209551 | 9.38E-37 |
| SPARC | IGFBP7-AS1 | 0.51609316 | 7.61E-27 |
| CETP | IGFBP7-AS1 | 0.57137811 | 8.53E-34 |
| ALOX12 | ARAP1-AS2 | 0.50997955 | 3.75E-26 |
| NCOR2 | ARAP1-AS2 | 0.527594 | 3.46E-28 |
| KAT2A | RNF139-AS1 | 0.66585803 | 2.93E-49 |
| PRKCD | RNF139-AS1 | 0.52289033 | 1.24E-27 |
| GNRH1 | RNF139-AS1 | 0.57592506 | 1.99E-34 |
| IDO1 | LINC00861 | 0.55901568 | 3.97E-32 |
| LTA | LINC00861 | 0.86243219 | 4.78E-112 |
| NCOR2 | SAP30L-AS1 | 0.50464816 | 1.47E-25 |
| BCAT1 | LINC00461 | 0.55315985 | 2.32E-31 |
| PLAU | LINC00461 | 0.62471233 | 7.16E-42 |
| IL10 | LINC00461 | 0.56867895 | 2.00E-33 |
| BIRC5 | LINC00461 | 0.51304856 | 1.69E-26 |
| SLC38A5 | LINC00461 | 0.57378202 | 3.97E-34 |
| GJA1 | LINC00461 | 0.50839091 | 5.65E-26 |
| CHGA | LINC00461 | 0.5511445 | 4.22E-31 |
| NES | LINC00461 | 0.51970384 | 2.92E-27 |
| SPARC | DACT3-AS1 | 0.53840805 | 1.70E-29 |
| PKM | DACT3-AS1 | 0.50611485 | 1.01E-25 |
| BRCA1 | DDX11-AS1 | 0.60899088 | 2.49E-39 |
| CDKN3 | DDX11-AS1 | 0.57632956 | 1.75E-34 |
| NCOR2 | DDX11-AS1 | 0.51371783 | 1.42E-26 |
| BIRC5 | DDX11-AS1 | 0.66779329 | 1.23E-49 |
| TYMS | DDX11-AS1 | 0.71820266 | 1.48E-60 |
| CDKN2A | DDX11-AS1 | 0.61926882 | 5.64E-41 |
| CDK4 | DDX11-AS1 | 0.6167476 | 1.45E-40 |
| EHMT2 | DDX11-AS1 | 0.53486025 | 4.62E-29 |
| PCNA | DDX11-AS1 | 0.67903022 | 7.02E-52 |
| DNMT1 | DDX11-AS1 | 0.65301567 | 7.89E-47 |
| CDK1 | DDX11-AS1 | 0.61718162 | 1.23E-40 |
| MKI67 | DDX11-AS1 | 0.54539491 | 2.28E-30 |
| HMMR | DDX11-AS1 | 0.55827139 | 4.98E-32 |
| PRKCD | DDX11-AS1 | 0.5028239 | 2.33E-25 |
| EZH2 | DDX11-AS1 | 0.70291742 | 5.31E-57 |
| KAT2A | RAD51-AS1 | 0.52754345 | 3.51E-28 |
| GNRH1 | RAD51-AS1 | 0.5160744 | 7.64E-27 |
| HRAS | SNHG6 | 0.61129252 | 1.08E-39 |
| RPLP0 | SNHG6 | 0.56902123 | 1.80E-33 |
| HSPB1 | SNHG6 | 0.60965331 | 1.96E-39 |
| RPLP2 | SNHG6 | 0.78757842 | 3.16E-80 |
| IL1B | LINC00900 | 0.50532856 | 1.24E-25 |
| FABP5 | LINC00900 | 0.51572795 | 8.38E-27 |
| IL10 | LINC00900 | 0.50268891 | 2.41E-25 |
| SIGLEC7 | LINC00900 | 0.52058784 | 2.31E-27 |
| PKM | LINC00900 | 0.52428561 | 8.52E-28 |
| SLC22A5 | SBF2-AS1 | 0.59417194 | 4.61E-37 |
| BRCA1 | SBF2-AS1 | 0.52664542 | 4.49E-28 |
| CS | SBF2-AS1 | 0.60179402 | 3.25E-38 |
| TYMS | SBF2-AS1 | 0.55670019 | 8.02E-32 |
| CDK4 | SBF2-AS1 | 0.53381662 | 6.19E-29 |
| EHMT2 | SBF2-AS1 | 0.50932993 | 4.43E-26 |
| HMMR | SBF2-AS1 | 0.50258694 | 2.47E-25 |
| EZH2 | SBF2-AS1 | 0.53836866 | 1.72E-29 |
| PPARG | LINC00968 | 0.50021592 | 4.49E-25 |
| FABP4 | LINC00968 | 0.5229239 | 1.23E-27 |
| LPL | LINC00968 | 0.54196355 | 6.15E-30 |
| PTHLH | RASSF8-AS1 | 0.85082115 | 5.31E-106 |
| CYP3A4 | RGMB-AS1 | 0.52837879 | 2.79E-28 |
| LPAR2 | LINC00920 | 0.51043193 | 3.34E-26 |
| ITGA2 | LINC00920 | 0.50726719 | 7.53E-26 |
| PLAU | BAALC-AS1 | 0.57781758 | 1.08E-34 |
| IL10 | BAALC-AS1 | 0.51481319 | 1.07E-26 |
| GJA1 | BAALC-AS1 | 0.58083802 | 4.04E-35 |
| CAMK2G | BAALC-AS1 | 0.50002267 | 4.71E-25 |
| NES | BAALC-AS1 | 0.50142899 | 3.31E-25 |
| SLC2A1 | MIR210HG | 0.56230314 | 1.45E-32 |
| GAPDH | MIR210HG | 0.54352101 | 3.93E-30 |
| BRCA1 | UBL7-AS1 | 0.53737636 | 2.27E-29 |
| SLC22A5 | OIP5-AS1 | 0.50489789 | 1.38E-25 |
| PRKCA | OIP5-AS1 | 0.53811478 | 1.84E-29 |
| BIRC5 | LRP4-AS1 | 0.54914596 | 7.62E-31 |
| CS | STX18-AS1 | 0.55354454 | 2.07E-31 |
| PFKM | STX18-AS1 | 0.51449553 | 1.16E-26 |
| IDO1 | PCED1B-AS1 | 0.55749971 | 6.30E-32 |
| SIGLEC7 | PCED1B-AS1 | 0.6623366 | 1.40E-48 |
| CD4 | PCED1B-AS1 | 0.5843302 | 1.28E-35 |
| CTLA4 | PCED1B-AS1 | 0.69643262 | 1.46E-55 |
| CCL3 | PCED1B-AS1 | 0.55015509 | 5.66E-31 |
| LTA | PCED1B-AS1 | 0.89128512 | 7.81E-130 |
| SPARC | NR2F2-AS1 | 0.50831559 | 5.76E-26 |
| ALOX12 | SEC24B-AS1 | 0.51849967 | 4.02E-27 |
| GNRH1 | SEC24B-AS1 | 0.5548751 | 1.39E-31 |
| LTA | LINC00926 | 0.80419356 | 4.56E-86 |
| GNRH1 | FAM13A-AS1 | 0.57295922 | 5.16E-34 |
| CS | UBA6-AS1 | 0.58175973 | 2.98E-35 |
| ABCC1 | UBA6-AS1 | 0.57537216 | 2.38E-34 |
| PFKM | UBA6-AS1 | 0.57204278 | 6.91E-34 |
| SLC22A5 | NNT-AS1 | 0.52045255 | 2.39E-27 |
| CS | NNT-AS1 | 0.55199695 | 3.28E-31 |
| CNR1 | LINC01194 | 0.65851439 | 7.44E-48 |
| HMMR | LINC01194 | 0.52543002 | 6.25E-28 |
| GNRH1 | OCIAD1-AS1 | 0.52783959 | 3.24E-28 |
| HRAS | TRIM52-AS1 | 0.52432726 | 8.43E-28 |
| RPLP0 | TRIM52-AS1 | 0.50209011 | 2.80E-25 |
| RPLP2 | TRIM52-AS1 | 0.62725146 | 2.70E-42 |
| CSNK2B | TRIM52-AS1 | 0.65610066 | 2.11E-47 |
| AKR1C1 | LUCAT1 | 0.78953141 | 6.93E-81 |
| TXN | LUCAT1 | 0.63651045 | 7.10E-44 |
| TKT | LUCAT1 | 0.71953656 | 7.08E-61 |
| AKR1C3 | LUCAT1 | 0.64559791 | 1.77E-45 |
| SPARC | LINC01197 | 0.60917808 | 2.32E-39 |
| CETP | LINC01197 | 0.53147879 | 1.19E-28 |
| CXCL12 | LINC01197 | 0.58803132 | 3.71E-36 |
| FBN1 | LINC01197 | 0.56818428 | 2.34E-33 |
| HSPG2 | LINC01197 | 0.50685288 | 8.38E-26 |
| PDGFRB | LINC01197 | 0.56187454 | 1.66E-32 |
| TBXA2R | LINC01197 | 0.62399629 | 9.42E-42 |
| NGFR | LINC01197 | 0.58987829 | 1.99E-36 |
| DCN | LINC01197 | 0.66694931 | 1.80E-49 |
| ACVRL1 | LINC01197 | 0.55452988 | 1.54E-31 |
| DNMT1 | BACH1-IT1 | 0.51840683 | 4.12E-27 |
| SLC22A5 | LINC00992 | 0.52437196 | 8.33E-28 |
| CS | LINC00992 | 0.51005595 | 3.68E-26 |
| GAP43 | NPHP3-AS1 | 0.60326277 | 1.93E-38 |
| PRKCA | USP46-AS1 | 0.53625825 | 3.12E-29 |
| ACSL1 | LINC01093 | 0.52432468 | 8.43E-28 |
| ABCC1 | LINC01060 | 0.50108012 | 3.61E-25 |
| NQO1 | LINC00942 | 0.61421167 | 3.70E-40 |
| PLAU | HOXC13-AS | 0.81024746 | 2.45E-88 |
| IL10 | HOXC13-AS | 0.69019311 | 3.28E-54 |
| GJA1 | HOXC13-AS | 0.59806423 | 1.20E-37 |
| NES | HOXC13-AS | 0.63487107 | 1.36E-43 |
| MAPK3 | NOP14-AS1 | 0.5272298 | 3.82E-28 |
| DGKQ | NOP14-AS1 | 0.57747986 | 1.21E-34 |
| EHMT2 | NOP14-AS1 | 0.60575641 | 7.96E-39 |
| ABCC1 | OSMR-AS1 | 0.615491 | 2.30E-40 |
| ANXA2 | OSMR-AS1 | 0.51124072 | 2.71E-26 |
| TH | LINC00964 | 0.50973055 | 4.00E-26 |
| TKT | PVT1 | 0.50647165 | 9.23E-26 |
| ANXA2 | PVT1 | 0.56204938 | 1.57E-32 |
| CYP2B6 | LINC01018 | 0.51959179 | 3.01E-27 |
| F9 | LINC01018 | 0.5470407 | 1.41E-30 |
| NCOR2 | DNAH10OS | 0.50077533 | 3.90E-25 |
| HAS3 | DNAH10OS | 0.50654871 | 9.05E-26 |
| LPL | FZD10-AS1 | 0.53256568 | 8.77E-29 |
| FGFR3 | AC090587.2 | 0.54588658 | 1.98E-30 |
| IL1RAPL2 | LINC01511 | 0.85975366 | 1.32E-110 |
| CCNA2 | LINC01182 | 0.55416464 | 1.72E-31 |
| FABP4 | ZBED3-AS1 | 0.53891649 | 1.47E-29 |
| CDK4 | SNHG21 | 0.51614721 | 7.50E-27 |
| TUBB | SNHG21 | 0.52747447 | 3.58E-28 |
| G6PD | ZFPM2-AS1 | 0.59882703 | 9.20E-38 |
| TKT | ZFPM2-AS1 | 0.58073065 | 4.18E-35 |
| SQSTM1 | ZFPM2-AS1 | 0.57786115 | 1.07E-34 |
| AKR1C3 | ZFPM2-AS1 | 0.50867208 | 5.25E-26 |
| ALOX12 | THAP9-AS1 | 0.52397451 | 9.27E-28 |
| ABCC1 | THAP9-AS1 | 0.55920479 | 3.75E-32 |
| GNRH1 | THAP9-AS1 | 0.56486363 | 6.59E-33 |
| CYP3A4 | HULC | 0.54897344 | 8.02E-31 |
| GLUL | HULC | 0.62113208 | 2.79E-41 |
| ACSL1 | F11-AS1 | 0.58376141 | 1.54E-35 |
| IL1B | LINC01094 | 0.6796152 | 5.33E-52 |
| FABP5 | LINC01094 | 0.5914636 | 1.16E-36 |
| BCAT1 | LINC01094 | 0.50987448 | 3.85E-26 |
| PLAU | LINC01094 | 0.50367008 | 1.88E-25 |
| IL10 | LINC01094 | 0.58620126 | 6.85E-36 |
| G6PD | LINC01094 | 0.5637956 | 9.18E-33 |
| ABCC1 | LINC01094 | 0.62095367 | 2.99E-41 |
| SIGLEC7 | LINC01094 | 0.60455442 | 1.22E-38 |
| NCOR2 | LINC01094 | 0.50582027 | 1.09E-25 |
| ADA | LINC01094 | 0.53640862 | 2.99E-29 |
| GJA1 | LINC01094 | 0.52670368 | 4.42E-28 |
| ANXA2 | LINC01094 | 0.64125815 | 1.05E-44 |
| LGALS1 | LINC01094 | 0.56189625 | 1.65E-32 |
| PKM | LINC01094 | 0.69636181 | 1.52E-55 |
| ABCC1 | PCAT1 | 0.52532035 | 6.44E-28 |
| BCAT1 | ZFHX4-AS1 | 0.53233013 | 9.37E-29 |
| BDNF | ZFHX4-AS1 | 0.70156224 | 1.07E-56 |
| IL11 | ZFHX4-AS1 | 0.62682659 | 3.18E-42 |
| STRA6 | ZFHX4-AS1 | 0.52287725 | 1.25E-27 |
| GLUL | LINC01484 | 0.52959317 | 2.00E-28 |
| PTK2 | OTUD6B-AS1 | 0.63680639 | 6.31E-44 |
| CD14 | LINC01485 | 0.51128089 | 2.68E-26 |
| SLC22A5 | WAC-AS1 | 0.52727637 | 3.78E-28 |
| MAPK3 | WAC-AS1 | 0.53620327 | 3.17E-29 |
| CS | WAC-AS1 | 0.59584514 | 2.59E-37 |
| ABCC1 | WAC-AS1 | 0.51301782 | 1.70E-26 |
| EHMT2 | WAC-AS1 | 0.55234991 | 2.95E-31 |
| CAMK2G | WAC-AS1 | 0.54215062 | 5.83E-30 |
| BRCA1 | AP001372.2 | 0.50492175 | 1.37E-25 |
| CYP3A4 | MIR100HG | 0.57223319 | 6.50E-34 |
| ALDH2 | MIR100HG | 0.50062602 | 4.05E-25 |
| RPLP2 | SNHG9 | 0.50846058 | 5.55E-26 |
| ABCC1 | AF131215.2 | 0.55142258 | 3.89E-31 |
| TFAP2A | AF131215.2 | 0.61306927 | 5.63E-40 |
| ITGA2 | AF131215.2 | 0.50368095 | 1.88E-25 |
| CDK4 | C8orf49 | 0.51996294 | 2.73E-27 |
| DNMT1 | C8orf49 | 0.51238114 | 2.01E-26 |
| MKI67 | C8orf49 | 0.51535014 | 9.25E-27 |
| EZH2 | C8orf49 | 0.59874303 | 9.47E-38 |
| FABP5 | TBX5-AS1 | 0.51837124 | 4.16E-27 |
| PLAU | TBX5-AS1 | 0.97182611 | 1.40E-235 |
| IL10 | TBX5-AS1 | 0.83654238 | 3.10E-99 |
| BIRC5 | TBX5-AS1 | 0.56751957 | 2.88E-33 |
| SLC38A5 | TBX5-AS1 | 0.59791775 | 1.26E-37 |
| GJA1 | TBX5-AS1 | 0.72703413 | 1.02E-62 |
| NES | TBX5-AS1 | 0.71661533 | 3.56E-60 |
| PTK2 | ZNF252P-AS1 | 0.5809315 | 3.92E-35 |
| KAT2A | ZNF252P-AS1 | 0.5419088 | 6.25E-30 |
| FGFR3 | SNHG1 | 0.50840115 | 5.63E-26 |
| KAT2A | SNHG1 | 0.56597759 | 4.66E-33 |
| CDK4 | SNHG1 | 0.56844412 | 2.15E-33 |
| NPM1 | SNHG1 | 0.54265183 | 5.05E-30 |
| EHMT2 | SNHG1 | 0.54379116 | 3.63E-30 |
| DNMT1 | SNHG1 | 0.54811295 | 1.03E-30 |
| CDK1 | SNHG1 | 0.5051827 | 1.28E-25 |
| RPLP0 | SNHG1 | 0.57599786 | 1.95E-34 |
| PRKCD | SNHG1 | 0.52410973 | 8.94E-28 |
| GNRH1 | SNHG1 | 0.54253488 | 5.22E-30 |
| EZH2 | SNHG1 | 0.54170502 | 6.62E-30 |
| LTA | IFNG-AS1 | 0.75754477 | 6.76E-71 |
| LGALS1 | AGAP2-AS1 | 0.50661355 | 8.90E-26 |
| PKM | AGAP2-AS1 | 0.5170736 | 5.87E-27 |
| BAX | PXN-AS1 | 0.54597375 | 1.93E-30 |
| BIRC5 | PXN-AS1 | 0.50455736 | 1.50E-25 |
| HRAS | PXN-AS1 | 0.6022281 | 2.79E-38 |
| RPLP0 | PXN-AS1 | 0.64127245 | 1.04E-44 |
| HSPB1 | PXN-AS1 | 0.52502493 | 6.97E-28 |
| FGFR4 | PXN-AS1 | 0.54329075 | 4.20E-30 |
| RPLP2 | PXN-AS1 | 0.63634569 | 7.58E-44 |
| CSNK2B | PXN-AS1 | 0.60512876 | 9.96E-39 |
| BCAT1 | LINC00944 | 0.52599859 | 5.35E-28 |
| PFKM | CCND2-AS1 | 0.5579346 | 5.52E-32 |
| PKM | CCND2-AS1 | 0.50092503 | 3.76E-25 |
| TH | AC156455.1 | 0.6701486 | 4.25E-50 |
| BCAT1 | AC156455.1 | 0.63862958 | 3.03E-44 |
| KCNJ11 | AC156455.1 | 0.51766405 | 5.02E-27 |
| CHGA | AC156455.1 | 0.88894212 | 3.28E-128 |
| EHMT2 | ZBTB11-AS1 | 0.59057361 | 1.57E-36 |
| BRCA1 | TMPO-AS1 | 0.59043614 | 1.65E-36 |
| CDKN3 | TMPO-AS1 | 0.58411545 | 1.37E-35 |
| TYMS | TMPO-AS1 | 0.62986923 | 9.77E-43 |
| PCNA | TMPO-AS1 | 0.61448376 | 3.34E-40 |
| DNMT1 | TMPO-AS1 | 0.58449625 | 1.21E-35 |
| CDK1 | TMPO-AS1 | 0.62338881 | 1.19E-41 |
| MKI67 | TMPO-AS1 | 0.50740596 | 7.27E-26 |
| HMMR | TMPO-AS1 | 0.54177128 | 6.50E-30 |
| EZH2 | TMPO-AS1 | 0.64044608 | 1.46E-44 |
| PLAU | CASC18 | 0.66673488 | 1.98E-49 |
| IL10 | CASC18 | 0.58554987 | 8.51E-36 |
| AGPAT4 | CASC18 | 0.51626897 | 7.26E-27 |
| GJA1 | CASC18 | 0.50743146 | 7.22E-26 |
| NES | CASC18 | 0.53548421 | 3.88E-29 |
| TH | MEG8 | 0.57331008 | 4.61E-34 |
| CHGA | MEG8 | 0.76950654 | 1.92E-74 |
| FGFR3 | LINC00641 | 0.51507416 | 9.95E-27 |
| NCOR2 | LINC00641 | 0.56256416 | 1.34E-32 |
| DNMT1 | LINC00641 | 0.52375984 | 9.83E-28 |
| GNRH1 | LINC00641 | 0.52019624 | 2.56E-27 |
| CNR1 | LINC00645 | 0.50436056 | 1.58E-25 |
| TH | LINC00930 | 0.64264318 | 5.95E-45 |
| BCAT1 | LINC00930 | 0.52390252 | 9.45E-28 |
| KCNJ11 | LINC00930 | 0.52348177 | 1.06E-27 |
| CHGA | LINC00930 | 0.85987061 | 1.15E-110 |
| BCAT1 | LINC01579 | 0.52361232 | 1.02E-27 |
| BDNF | LINC01579 | 0.5840197 | 1.41E-35 |
| IL11 | LINC01579 | 0.55586909 | 1.03E-31 |
| STRA6 | LINC01579 | 0.5033492 | 2.04E-25 |
| IL1B | HIF1A-AS1 | 0.55296648 | 2.46E-31 |
| FABP5 | HIF1A-AS1 | 0.50563929 | 1.14E-25 |
| BCAT1 | HIF1A-AS1 | 0.64965145 | 3.27E-46 |
| SIGLEC7 | HIF1A-AS1 | 0.50029078 | 4.40E-25 |
| BDNF | HIF1A-AS1 | 0.83146451 | 5.51E-97 |
| ADA | HIF1A-AS1 | 0.50281889 | 2.33E-25 |
| COL1A1 | HIF1A-AS1 | 0.51735914 | 5.44E-27 |
| IL11 | HIF1A-AS1 | 0.81100717 | 1.26E-88 |
| GJA1 | HIF1A-AS1 | 0.50181881 | 3.00E-25 |
| ANXA2 | HIF1A-AS1 | 0.56318371 | 1.11E-32 |
| LGALS1 | HIF1A-AS1 | 0.71504623 | 8.40E-60 |
| PKM | HIF1A-AS1 | 0.73741765 | 2.26E-65 |
| STRA6 | HIF1A-AS1 | 0.79400257 | 2.02E-82 |
| TDO2 | LINC01146 | 0.50267895 | 2.42E-25 |
| DNAH8 | EGLN3-AS1 | 0.55794766 | 5.50E-32 |
| FABP5 | LINC00519 | 0.52460364 | 7.82E-28 |
| PLAU | LINC00519 | 0.86692557 | 1.55E-114 |
| IL10 | LINC00519 | 0.80405655 | 5.12E-86 |
| BIRC5 | LINC00519 | 0.52278379 | 1.28E-27 |
| SLC38A5 | LINC00519 | 0.51543306 | 9.05E-27 |
| GJA1 | LINC00519 | 0.68518526 | 3.75E-53 |
| NES | LINC00519 | 0.61387718 | 4.18E-40 |
| SLC22A12 | LINC00639 | 0.53233061 | 9.37E-29 |
| CNR1 | LINC00648 | 0.69089271 | 2.32E-54 |
| SPARC | LINC00924 | 0.54131303 | 7.41E-30 |
| CXCL12 | LINC00924 | 0.50769914 | 6.74E-26 |
| FBN1 | LINC00924 | 0.58494574 | 1.04E-35 |
| HSPG2 | LINC00924 | 0.568674 | 2.00E-33 |
| PDGFRB | LINC00924 | 0.52476728 | 7.48E-28 |
| TBXA2R | LINC00924 | 0.50360864 | 1.91E-25 |
| NGFR | LINC00924 | 0.64367389 | 3.90E-45 |
| DCN | LINC00924 | 0.69392288 | 5.16E-55 |
| ACVRL1 | LINC00924 | 0.54990387 | 6.09E-31 |
| ALOX15B | CERS3-AS1 | 0.51484421 | 1.06E-26 |
| ABCC1 | ADNP-AS1 | 0.51975316 | 2.88E-27 |
| PRKCD | ADNP-AS1 | 0.51503101 | 1.01E-26 |
| FABP4 | CPEB1-AS1 | 0.52058842 | 2.31E-27 |
| SIGLEC7 | IL21R-AS1 | 0.51095228 | 2.92E-26 |
| CTLA4 | IL21R-AS1 | 0.57057361 | 1.10E-33 |
| LTA | IL21R-AS1 | 0.50982477 | 3.90E-26 |
| ALDH2 | LINC00261 | 0.53249745 | 8.94E-29 |
| PLG | LINC00261 | 0.5466493 | 1.58E-30 |
| KAT2A | AC027601.1 | 0.59088249 | 1.42E-36 |
| GNRH1 | AC027601.1 | 0.50845443 | 5.56E-26 |
| RPLP2 | SNHG19 | 0.56886841 | 1.89E-33 |
| BIRC5 | ATP2A1-AS1 | 0.54713607 | 1.37E-30 |
| PLAU | LCMT1-AS1 | 0.55703542 | 7.25E-32 |
| IL10 | LCMT1-AS1 | 0.55438575 | 1.61E-31 |
| BIRC5 | LCMT1-AS1 | 0.50473818 | 1.44E-25 |
| KAT2A | STAM-AS1 | 0.50862882 | 5.31E-26 |
| ABCC1 | LINC01541 | 0.54583459 | 2.01E-30 |
| PRKCA | FBXL19-AS1 | 0.50106765 | 3.62E-25 |
| CS | FBXL19-AS1 | 0.57261503 | 5.76E-34 |
| NCOR2 | FBXL19-AS1 | 0.56470323 | 6.93E-33 |
| IL1RAPL2 | FBXL19-AS1 | 0.52700504 | 4.07E-28 |
| TFAP2A | AC009120.5 | 0.51936784 | 3.19E-27 |
| FABP5 | ADPGK-AS1 | 0.55582236 | 1.04E-31 |
| PLAU | ADPGK-AS1 | 0.82536443 | 2.22E-94 |
| IL10 | ADPGK-AS1 | 0.82262329 | 3.06E-93 |
| BIRC5 | ADPGK-AS1 | 0.58672905 | 5.74E-36 |
| SLC38A5 | ADPGK-AS1 | 0.5156799 | 8.48E-27 |
| GJA1 | ADPGK-AS1 | 0.66333872 | 8.98E-49 |
| NES | ADPGK-AS1 | 0.61200923 | 8.30E-40 |
| BDNF | LINC00565 | 0.53795082 | 1.93E-29 |
| IL11 | LINC00565 | 0.58707173 | 5.12E-36 |
| LGALS1 | LINC00565 | 0.57233203 | 6.30E-34 |
| PKM | LINC00565 | 0.50444901 | 1.54E-25 |
| STRA6 | LINC00565 | 0.56548631 | 5.43E-33 |
| KAT2A | LINC01311 | 0.52598435 | 5.37E-28 |
| MIF | LINC01311 | 0.54619067 | 1.81E-30 |
| RPLP2 | LINC01311 | 0.51877337 | 3.74E-27 |
| BRCA1 | LINC01572 | 0.52796387 | 3.13E-28 |
| MKI67 | LINC01572 | 0.59801414 | 1.22E-37 |
| HMMR | LINC01572 | 0.5737075 | 4.06E-34 |
| SLC16A3 | AC140912.1 | 0.51210664 | 2.16E-26 |
| KAT2A | LINC01355 | 0.53348766 | 6.78E-29 |
| PLAU | AC006538.1 | 0.63709091 | 5.63E-44 |
| IL10 | AC006538.1 | 0.53767269 | 2.09E-29 |
| BIRC5 | AC006538.1 | 0.54408665 | 3.33E-30 |
| NES | AC006538.1 | 0.57863836 | 8.28E-35 |
| CHGA | VPS9D1-AS1 | 0.55943491 | 3.50E-32 |
| FFAR4 | AC144833.1 | 0.81376208 | 1.08E-89 |
| GNRH1 | PAN3-AS1 | 0.56158148 | 1.81E-32 |
| IDO1 | DLGAP1-AS5 | 0.74579212 | 1.31E-67 |
| CYP17A1 | DLGAP1-AS5 | 0.62299349 | 1.38E-41 |
| IL1RAPL2 | TTC39A-AS1 | 0.54899487 | 7.97E-31 |
| HAS3 | TTC39A-AS1 | 0.56337219 | 1.05E-32 |
| IL1B | LINC00165 | 0.55013454 | 5.69E-31 |
| BDNF | LINC00165 | 0.57950425 | 6.25E-35 |
| FABP5 | LOXL1-AS1 | 0.50065938 | 4.02E-25 |
| PLAU | LOXL1-AS1 | 0.84920854 | 3.34E-105 |
| IL10 | LOXL1-AS1 | 0.79064924 | 2.89E-81 |
| BIRC5 | LOXL1-AS1 | 0.5232691 | 1.12E-27 |
| COL1A1 | LOXL1-AS1 | 0.53389179 | 6.06E-29 |
| IL11 | LOXL1-AS1 | 0.57657838 | 1.61E-34 |
| GJA1 | LOXL1-AS1 | 0.75978963 | 1.52E-71 |
| LGALS1 | LOXL1-AS1 | 0.62765429 | 2.31E-42 |
| PKM | LOXL1-AS1 | 0.70669744 | 7.38E-58 |
| NES | LOXL1-AS1 | 0.61352605 | 4.76E-40 |
| CS | LINC00662 | 0.50775365 | 6.65E-26 |
| LPAR2 | AC144831.1 | 0.5497461 | 6.38E-31 |
| PFKM | AC144831.1 | 0.60363874 | 1.69E-38 |
| ASIC1 | DLGAP1-AS2 | 0.5449228 | 2.62E-30 |
| SLC22A5 | TAPT1-AS1 | 0.51470484 | 1.10E-26 |
| DGKQ | TAPT1-AS1 | 0.50165584 | 3.13E-25 |
| TFAP2A | TAPT1-AS1 | 0.53098601 | 1.36E-28 |
| ITGA2 | TAPT1-AS1 | 0.53285316 | 8.10E-29 |
| HSPG2 | HID1-AS1 | 0.5292679 | 2.19E-28 |
| CYP19A1 | DLGAP1-AS3 | 0.57943137 | 6.40E-35 |
| BRCA1 | LINC00909 | 0.54034858 | 9.77E-30 |
| NCOR2 | LINC00909 | 0.54704957 | 1.41E-30 |
| TYMS | LINC00909 | 0.56718575 | 3.20E-33 |
| CDK4 | LINC00909 | 0.53758214 | 2.15E-29 |
| EHMT2 | LINC00909 | 0.5800514 | 5.22E-35 |
| PCNA | LINC00909 | 0.52698669 | 4.09E-28 |
| DNMT1 | LINC00909 | 0.60555509 | 8.55E-39 |
| CAMK2G | LINC00909 | 0.50053313 | 4.14E-25 |
| TUBB | LINC00909 | 0.5382054 | 1.80E-29 |
| EZH2 | LINC00909 | 0.51388943 | 1.36E-26 |
| IL1B | L3MBTL4-AS1 | 0.52694857 | 4.13E-28 |
| FABP5 | L3MBTL4-AS1 | 0.53414763 | 5.64E-29 |
| SIGLEC7 | L3MBTL4-AS1 | 0.68392667 | 6.87E-53 |
| CD4 | L3MBTL4-AS1 | 0.51453989 | 1.14E-26 |
| ANXA2 | L3MBTL4-AS1 | 0.50409896 | 1.69E-25 |
| PKM | L3MBTL4-AS1 | 0.52687827 | 4.21E-28 |
| GAD1 | GACAT2 | 0.5985885 | 1.00E-37 |
| FFAR4 | GACAT2 | 0.7346152 | 1.21E-64 |
| DNMT1 | NARF-IT1 | 0.51270852 | 1.85E-26 |
| AKR1C1 | LINC01532 | 0.67237479 | 1.54E-50 |
| TKT | LINC01532 | 0.55831378 | 4.92E-32 |
| BRCA1 | ASB16-AS1 | 0.53236432 | 9.28E-29 |
| FGFR3 | ZNF790-AS1 | 0.50265443 | 2.43E-25 |
| GLS | MAP3K14-AS1 | 0.50284168 | 2.32E-25 |
| ABCC1 | MAP3K14-AS1 | 0.54505722 | 2.52E-30 |
| HSPG2 | LINC01415 | 0.57933257 | 6.61E-35 |
| FABP4 | MIR497HG | 0.55724505 | 6.80E-32 |
| ABCC1 | AC061992.2 | 0.53501667 | 4.42E-29 |
| GAD1 | RUNDC3A-AS1 | 0.5017278 | 3.07E-25 |
| MAPK3 | PTOV1-AS1 | 0.57128557 | 8.79E-34 |
| BRCA1 | PTOV1-AS1 | 0.52913089 | 2.27E-28 |
| NCOR2 | PTOV1-AS1 | 0.52747933 | 3.57E-28 |
| KAT2A | PTOV1-AS1 | 0.60516955 | 9.81E-39 |
| CDK4 | PTOV1-AS1 | 0.51695531 | 6.06E-27 |
| EHMT2 | PTOV1-AS1 | 0.56159236 | 1.81E-32 |
| PCNA | PTOV1-AS1 | 0.50672492 | 8.65E-26 |
| DNMT1 | PTOV1-AS1 | 0.50004971 | 4.68E-25 |
| PRKCD | PTOV1-AS1 | 0.50593008 | 1.06E-25 |
| CAMK2G | PTOV1-AS1 | 0.51663027 | 6.60E-27 |
| TUBB | PTOV1-AS1 | 0.54336646 | 4.11E-30 |
| GNRH1 | PTOV1-AS1 | 0.5764642 | 1.68E-34 |
| PKM | PTOV1-AS1 | 0.50416612 | 1.66E-25 |
| EZH2 | PTOV1-AS1 | 0.52187042 | 1.63E-27 |
| SOX9 | PTOV1-AS1 | 0.5338607 | 6.11E-29 |
| GLS | AC005253.2 | 0.50078325 | 3.89E-25 |
| NCOR2 | AC005253.2 | 0.53417259 | 5.60E-29 |
| DNMT1 | AC005253.2 | 0.51886303 | 3.65E-27 |
| CNR1 | ZNF649-AS1 | 0.50144135 | 3.30E-25 |
| PLAU | FENDRR | 0.86810203 | 3.33E-115 |
| IL10 | FENDRR | 0.80890488 | 7.94E-88 |
| CXCL12 | FENDRR | 0.63503598 | 1.28E-43 |
| GJA1 | FENDRR | 0.70028229 | 2.07E-56 |
| DCN | FENDRR | 0.57688022 | 1.46E-34 |
| NES | FENDRR | 0.68358644 | 8.08E-53 |
| CSNK2B | ZSCAN16-AS1 | 0.66976408 | 5.06E-50 |
| NCOR2 | PTOV1-AS2 | 0.50729011 | 7.49E-26 |
| KAT2A | PTOV1-AS2 | 0.63079454 | 6.81E-43 |
| DNMT1 | PTOV1-AS2 | 0.59808078 | 1.19E-37 |
| GNRH1 | PTOV1-AS2 | 0.67212821 | 1.72E-50 |
| EZH2 | PTOV1-AS2 | 0.50563087 | 1.14E-25 |
| BRCA1 | LINC01224 | 0.58040489 | 4.65E-35 |
| CDKN3 | LINC01224 | 0.55472281 | 1.45E-31 |
| TYMS | LINC01224 | 0.55482528 | 1.41E-31 |
| CDK4 | LINC01224 | 0.51832938 | 4.21E-27 |
| PCNA | LINC01224 | 0.50774206 | 6.67E-26 |
| DNMT1 | LINC01224 | 0.51588742 | 8.03E-27 |
| CDK1 | LINC01224 | 0.5799406 | 5.42E-35 |
| HMMR | LINC01224 | 0.52243206 | 1.41E-27 |
| EZH2 | LINC01224 | 0.59111263 | 1.31E-36 |
| SOX9 | KCNQ1OT1 | 0.50678006 | 8.53E-26 |
| RPLP0 | SNHG8 | 0.62241016 | 1.72E-41 |
| RPLP2 | SNHG8 | 0.58533685 | 9.14E-36 |
| PKM | SPACA6P-AS | 0.58584631 | 7.71E-36 |
| PKM | MIR222HG | 0.51267099 | 1.87E-26 |
| DVL2 | MIR222HG | 0.50022432 | 4.48E-25 |
| ABCC1 | HMGN3-AS1 | 0.54165633 | 6.72E-30 |
| TFAP2A | HMGN3-AS1 | 0.51983493 | 2.82E-27 |
| GNRH1 | HMGN3-AS1 | 0.53736114 | 2.28E-29 |
| VWF | LINC01235 | 0.52225095 | 1.48E-27 |
| G6PD | TMCC1-AS1 | 0.58085283 | 4.02E-35 |
| AKR1C1 | TMCC1-AS1 | 0.5675707 | 2.83E-33 |
| PTK2 | TMCC1-AS1 | 0.52112156 | 2.00E-27 |
| NPM1 | TMCC1-AS1 | 0.57276482 | 5.49E-34 |
| TKT | TMCC1-AS1 | 0.61561601 | 2.20E-40 |
| AKR1C3 | TMCC1-AS1 | 0.56887673 | 1.88E-33 |
| LTA | LINC01215 | 0.83713954 | 1.66E-99 |
| ALOX12 | NFYC-AS1 | 0.54931214 | 7.26E-31 |
| SLC2A1 | CASC15 | 0.58680525 | 5.60E-36 |
| PLAU | CASC15 | 0.57023051 | 1.23E-33 |
| IL10 | CASC15 | 0.55824762 | 5.02E-32 |
| GJA1 | CASC15 | 0.60154255 | 3.55E-38 |
| NES | CASC15 | 0.61266854 | 6.52E-40 |
| CAMK2G | ZSWIM8-AS1 | 0.50904996 | 4.77E-26 |
| TH | MESTIT1 | 0.60408753 | 1.44E-38 |
| BCAT1 | MESTIT1 | 0.503584 | 1.92E-25 |
| KCNJ11 | MESTIT1 | 0.56380568 | 9.15E-33 |
| CHGA | MESTIT1 | 0.81264343 | 2.94E-89 |
| BAX | JMJD1C-AS1 | 0.52707349 | 3.99E-28 |
| TYMS | JMJD1C-AS1 | 0.50022341 | 4.48E-25 |
| ATP6V1B1 | JMJD1C-AS1 | 0.50574156 | 1.11E-25 |
| DVL2 | JMJD1C-AS1 | 0.52046537 | 2.38E-27 |
| CNR1 | MYHAS | 0.5048874 | 1.38E-25 |
| HAS3 | DGCR9 | 0.51863338 | 3.88E-27 |
| GNRH1 | FRMD6-AS1 | 0.54458523 | 2.89E-30 |
| BCAT1 | FP236383.3 | 0.6140718 | 3.89E-40 |
| BDNF | FP236383.3 | 0.89833505 | 5.94E-135 |
| IL11 | FP236383.3 | 0.99711489 | 0 |
| LGALS1 | FP236383.3 | 0.61137789 | 1.04E-39 |
| PKM | FP236383.3 | 0.54791804 | 1.09E-30 |
| STRA6 | FP236383.3 | 0.84299862 | 3.28E-102 |
| MYCN | LINC01297 | 0.5544676 | 1.57E-31 |
| BCAT1 | AJ011931.1 | 0.6190405 | 6.14E-41 |
| BDNF | AJ011931.1 | 0.89878491 | 2.72E-135 |
| IL11 | AJ011931.1 | 0.99781813 | 0 |
| LGALS1 | AJ011931.1 | 0.61652651 | 1.57E-40 |
| PKM | AJ011931.1 | 0.55437598 | 1.61E-31 |
| STRA6 | AJ011931.1 | 0.84567747 | 1.74E-103 |
| CNR1 | SPANXA2-OT1 | 0.66148954 | 2.03E-48 |
| FABP4 | PGM5P3-AS1 | 0.72143473 | 2.45E-61 |
| LPL | PGM5P3-AS1 | 0.54394379 | 3.48E-30 |
| CETP | PGM5P3-AS1 | 0.61139849 | 1.04E-39 |
| PTGS2 | TSC22D1-AS1 | 0.67194316 | 1.88E-50 |
| MMP1 | TSC22D1-AS1 | 0.71272556 | 2.96E-59 |
| ITGA2 | TSC22D1-AS1 | 0.54562359 | 2.14E-30 |
| BGLAP | BACE1-AS | 0.50039546 | 4.29E-25 |
| KAT2A | BACE1-AS | 0.60496664 | 1.05E-38 |
| EHMT2 | BACE1-AS | 0.57699889 | 1.41E-34 |
| PRKCD | BACE1-AS | 0.52056613 | 2.32E-27 |
| PLAU | BANCR | 0.52302857 | 1.20E-27 |
| GJA1 | BANCR | 0.57887353 | 7.67E-35 |
| PTHLH | BANCR | 0.53539157 | 3.98E-29 |
| HSPA4 | AC012065.1 | 0.52807712 | 3.03E-28 |
| CTLA4 | AC130469.1 | 0.52128376 | 1.91E-27 |
| NCOR2 | UCKL1-AS1 | 0.55722116 | 6.85E-32 |
| ABCC1 | AL031587.1 | 0.52114591 | 1.99E-27 |
| NCOR2 | AL031587.1 | 0.51848729 | 4.04E-27 |
| TGFB2 | AL031587.1 | 0.56841961 | 2.17E-33 |
| TFAP2A | AL031587.1 | 0.58261479 | 2.25E-35 |
| CAMK2G | AL031587.1 | 0.5038397 | 1.80E-25 |
| PKM | AL031587.1 | 0.53097136 | 1.37E-28 |
| F9 | LINC01348 | 0.50774587 | 6.66E-26 |
| NCOR2 | AP000432.1 | 0.52555963 | 6.03E-28 |
| MMP9 | PCAT14 | 0.57372005 | 4.05E-34 |
| TGFB2 | PCAT14 | 0.59205251 | 9.51E-37 |
| IBSP | PCAT14 | 0.55218186 | 3.10E-31 |
| CNR1 | LINC01242 | 0.70252032 | 6.53E-57 |
| IL1B | AC133644.2 | 0.58724743 | 4.83E-36 |
| BCAT1 | AC133644.2 | 0.58043419 | 4.61E-35 |
| SIGLEC7 | AC133644.2 | 0.55677534 | 7.84E-32 |
| BDNF | AC133644.2 | 0.63288005 | 3.00E-43 |
| CTLA4 | AC133644.2 | 0.67284413 | 1.24E-50 |
| ADA | AC133644.2 | 0.5351633 | 4.24E-29 |
| CCL3 | AC133644.2 | 0.5076626 | 6.81E-26 |
| ANXA2 | AC133644.2 | 0.54430951 | 3.13E-30 |
| LGALS1 | AC133644.2 | 0.513334 | 1.57E-26 |
| PKM | AC133644.2 | 0.53286969 | 8.06E-29 |
| LTA | AC133644.2 | 0.54172952 | 6.58E-30 |
| PTGS2 | AC090181.1 | 0.66598486 | 2.77E-49 |
| MMP1 | AC090181.1 | 0.74471829 | 2.57E-67 |
| PLAU | JAKMIP2-AS1 | 0.955544 | 2.12E-199 |
| IL10 | JAKMIP2-AS1 | 0.81876741 | 1.13E-91 |
| BIRC5 | JAKMIP2-AS1 | 0.52563536 | 5.91E-28 |
| SLC38A5 | JAKMIP2-AS1 | 0.55226014 | 3.03E-31 |
| GJA1 | JAKMIP2-AS1 | 0.70452396 | 2.30E-57 |
| NES | JAKMIP2-AS1 | 0.67701764 | 1.80E-51 |
| BRCA1 | LINC00294 | 0.5254891 | 6.15E-28 |
| NCOR2 | LINC00294 | 0.59781956 | 1.31E-37 |
| PCNA | LINC00294 | 0.54575924 | 2.05E-30 |
| DNMT1 | LINC00294 | 0.59907965 | 8.42E-38 |
| CDK1 | LINC00294 | 0.5089468 | 4.89E-26 |
| GJA1 | LINC00294 | 0.50848974 | 5.51E-26 |
| PRKCD | LINC00294 | 0.51481332 | 1.07E-26 |
| CAMK2G | LINC00294 | 0.56688029 | 3.52E-33 |
| EZH2 | LINC00294 | 0.51850646 | 4.02E-27 |
| DVL2 | LINC00294 | 0.52570714 | 5.79E-28 |
| TYMS | LINC00628 | 0.51004536 | 3.69E-26 |
| DGKQ | CTBP1-AS | 0.56754967 | 2.85E-33 |
| PTGS2 | N4BP2L2-IT2 | 0.53762086 | 2.12E-29 |
| MMP1 | N4BP2L2-IT2 | 0.56026411 | 2.72E-32 |
| GNRH1 | N4BP2L2-IT2 | 0.54574729 | 2.06E-30 |
| IDO1 | TRG-AS1 | 0.50985714 | 3.87E-26 |
| SIGLEC7 | TRG-AS1 | 0.65161542 | 1.43E-46 |
| CD4 | TRG-AS1 | 0.56412418 | 8.29E-33 |
| CTLA4 | TRG-AS1 | 0.70563962 | 1.29E-57 |
| CCL3 | TRG-AS1 | 0.59609678 | 2.37E-37 |
| LTA | TRG-AS1 | 0.82474191 | 4.05E-94 |
| DNAH8 | SCHLAP1 | 0.51161956 | 2.45E-26 |
| HSPG2 | NPTN-IT1 | 0.51951665 | 3.07E-27 |
| BRCA1 | GHET1 | 0.58687695 | 5.46E-36 |
| HMMR | GHET1 | 0.56018445 | 2.78E-32 |
| GLUL | LINC01338 | 0.50176022 | 3.05E-25 |
| CS | ABALON | 0.59080463 | 1.45E-36 |
| NCOR2 | ABALON | 0.50467455 | 1.46E-25 |
| ACLY | ABALON | 0.52803654 | 3.07E-28 |
| ITGA2 | ABALON | 0.58447868 | 1.22E-35 |
| ABCC1 | SNHG4 | 0.53973668 | 1.16E-29 |
| NPM1 | SNHG4 | 0.62362339 | 1.09E-41 |
| DNMT1 | PCBP2-OT1 | 0.50258061 | 2.48E-25 |
