# Supplementary material for: A new acidic microenvironment related lncRNA signature predicts the prognosis of liver cancer patients
Source: Front Oncol. 2022 Oct 31;12:1016721. doi: 10.3389/fonc.2022.1016721 (PMC9660327; doi:10.3389/fonc.2022.1016721)
Supplement: Supplementary file 4 [file Table_4.docx]

**Supplementary Table S4** Univariate Cox regression of AME-associated lncRNAs for liver cancer patients.

| gene | KM | B | SE | HR | HR.95L | HR.95H | pvalue |
| --- | --- | --- | --- | --- | --- | --- | --- |
| LINC01587 | 0.00780462 | 0.10908046 | 0.03768701 | 1.11525209 | 1.03584268 | 1.20074914 | 0.0037991 |
| DLEU2L | 0.0045565 | 0.15257362 | 0.0645732 | 1.16482821 | 1.02635375 | 1.32198549 | 0.01813741 |
| LINC01116 | 0.01466342 | 0.17030131 | 0.04610701 | 1.18566205 | 1.08321475 | 1.29779852 | 0.00022109 |
| SNHG16 | 0.01579082 | 0.2378015 | 0.09591451 | 1.26845738 | 1.05107401 | 1.53080002 | 0.0131638 |
| LINC01106 | 0.0184027 | 0.14055678 | 0.0563965 | 1.15091443 | 1.03047688 | 1.28542819 | 0.01269201 |
| ZFAS1 | 0.01496629 | 0.24587096 | 0.08327753 | 1.27873455 | 1.08616158 | 1.5054501 | 0.00315285 |
| KTN1-AS1 | 0.00609748 | 0.28841107 | 0.09597769 | 1.33430568 | 1.10550058 | 1.6104665 | 0.00265598 |
| LINC00943 | 0.01935979 | 0.14714576 | 0.06077734 | 1.15852282 | 1.02842075 | 1.30508367 | 0.01547519 |
| SNHG17 | 0.00036764 | 0.26710795 | 0.09249337 | 1.30618145 | 1.08961485 | 1.56579178 | 0.00387872 |
| C2orf27A | 0.00021687 | 0.25375781 | 0.05671053 | 1.28885961 | 1.15327671 | 1.44038208 | 7.65E-06 |
| SNHG12 | 4.61E-05 | 0.3359388 | 0.09405621 | 1.39925339 | 1.16368536 | 1.68250809 | 0.0003547 |
| C3orf35 | 0.00048922 | 0.29911411 | 0.08771887 | 1.34866351 | 1.13563081 | 1.60165896 | 0.00064983 |
| LINC00862 | 0.00192355 | 0.19966524 | 0.05652119 | 1.22099395 | 1.09295578 | 1.36403161 | 0.00041154 |
| STARD7-AS1 | 0.01429733 | 0.35481397 | 0.13508848 | 1.42591536 | 1.09422397 | 1.85815214 | 0.00862603 |
| LINC01460 | 0.02233314 | 0.24777826 | 0.07258793 | 1.28117581 | 1.1112756 | 1.47705166 | 0.00064136 |
| THUMPD3-AS1 | 0.00163749 | 0.42903986 | 0.10925173 | 1.53578225 | 1.2397508 | 1.90250098 | 8.60E-05 |
| AC016747.3 | 0.04962295 | 0.43726984 | 0.1173273 | 1.54847386 | 1.23036708 | 1.94882597 | 0.00019383 |
| AC074286.1 | 0.01592978 | 0.36858545 | 0.10112631 | 1.44568817 | 1.18575717 | 1.76259891 | 0.0002676 |
| AC024560.3 | 0.03780759 | 0.26552075 | 0.09743299 | 1.30410991 | 1.07740523 | 1.57851716 | 0.00642698 |
| AC005562.1 | 0.00722162 | 0.20063268 | 0.0810674 | 1.22217576 | 1.042627 | 1.43264424 | 0.01332782 |
| AC010468.1 | 0.00096296 | 0.22130505 | 0.07863533 | 1.24770398 | 1.06949078 | 1.4556135 | 0.00488804 |
| MCM3AP-AS1 | 0.00030036 | 0.25851383 | 0.09160115 | 1.29500406 | 1.08218147 | 1.5496805 | 0.00477002 |
| LINC01139 | 0.01251308 | 0.07913845 | 0.0286981 | 1.08235417 | 1.02315523 | 1.14497831 | 0.00582238 |
| NUTM2A-AS1 | 0.01887055 | 0.27010126 | 0.11836921 | 1.31009711 | 1.03883706 | 1.65218828 | 0.02249795 |
| LINC00205 | 0.01131284 | 0.24664962 | 0.07653196 | 1.27973064 | 1.10147449 | 1.48683471 | 0.0012693 |
| ROR1-AS1 | 0.00671998 | 0.14297723 | 0.06204098 | 1.15370353 | 1.02160932 | 1.30287754 | 0.02119116 |
| HTR2A-AS1 | 0.00686581 | -0.366845 | 0.11408537 | 0.69291707 | 0.55407891 | 0.86654456 | 0.00130204 |
| AC090945.1 | 0.00104104 | 0.30783775 | 0.07239387 | 1.36048023 | 1.18051215 | 1.56788429 | 2.12E-05 |
| MIR137HG | 0.01012632 | 0.26684706 | 0.06055383 | 1.30584072 | 1.15970276 | 1.470394 | 1.05E-05 |
| LHFPL3-AS2 | 0.01452735 | 0.10351606 | 0.03042514 | 1.1090636 | 1.04486101 | 1.17721119 | 0.00066816 |
| NRSN2-AS1 | 0.02197168 | 0.27104274 | 0.07698421 | 1.31133112 | 1.12767328 | 1.52490029 | 0.00043032 |
| NUTM2B-AS1 | 0.0341387 | 0.33101718 | 0.12047206 | 1.39238371 | 1.0995448 | 1.76321365 | 0.00600208 |
| RUSC1-AS1 | 0.0083737 | 0.19000416 | 0.0817353 | 1.20925463 | 1.03025455 | 1.41935482 | 0.02009187 |
| SLC16A1-AS1 | 0.00580873 | 0.25546629 | 0.08521274 | 1.29106349 | 1.09248225 | 1.52574099 | 0.00271773 |
| AC126365.1 | 0.03262329 | 0.15907024 | 0.06668538 | 1.17242029 | 1.02877554 | 1.33612171 | 0.0170613 |
| LENG8-AS1 | 0.01306519 | 0.21034302 | 0.09364013 | 1.23410131 | 1.0271744 | 1.48271418 | 0.02468535 |
| DANCR | 0.00075492 | 0.24089987 | 0.06743794 | 1.27239362 | 1.11485453 | 1.45219442 | 0.00035403 |
| LMCD1-AS1 | 0.04742915 | 0.24605974 | 0.08108625 | 1.27897598 | 1.09104244 | 1.49928131 | 0.002409 |
| SLC2A1-AS1 | 0.00346688 | 0.21231626 | 0.05698553 | 1.23653889 | 1.10586372 | 1.38265538 | 0.0001947 |
| AC108463.2 | 0.00317283 | 0.23912362 | 0.08015987 | 1.27013554 | 1.08547009 | 1.48621718 | 0.0028536 |
| AC012368.1 | 0.02263481 | 0.19468407 | 0.08857807 | 1.2149271 | 1.02129789 | 1.44526673 | 0.02795756 |
| RNF144A-AS1 | 0.00314515 | 0.15547709 | 0.04032715 | 1.16821517 | 1.07943452 | 1.26429778 | 0.00011554 |
| AC092155.1 | 0.01635708 | 0.17382893 | 0.07371631 | 1.189852 | 1.02978251 | 1.3748027 | 0.01836975 |
| LINC01150 | 0.04316531 | 0.20838067 | 0.08041971 | 1.23168194 | 1.05207134 | 1.44195583 | 0.00956519 |
| AC007128.1 | 0.00185901 | 0.20534919 | 0.04493381 | 1.22795378 | 1.12443483 | 1.34100299 | 4.88E-06 |
| PRRT3-AS1 | 0.00414887 | 0.19270718 | 0.05827407 | 1.21252769 | 1.08165481 | 1.3592353 | 0.00094331 |
| NFE4 | 0.01441671 | 0.10748373 | 0.04686839 | 1.11347275 | 1.01574606 | 1.22060189 | 0.02183019 |
| AC092171.4 | 0.00603638 | 0.24365857 | 0.07512654 | 1.27590863 | 1.10121406 | 1.47831642 | 0.0011815 |
| LINC01293 | 0.03032629 | 0.17683191 | 0.07259305 | 1.19343048 | 1.03515601 | 1.37590497 | 0.01485337 |
| ZNF674-AS1 | 0.01074518 | 0.28961167 | 0.11216282 | 1.33590862 | 1.07226859 | 1.66437015 | 0.00982111 |
| HCG18 | 0.0103096 | 0.22360265 | 0.09113869 | 1.25057401 | 1.04600078 | 1.49515697 | 0.01414999 |
| LINC01436 | 0.01856718 | 0.1063716 | 0.03055398 | 1.11223511 | 1.04758435 | 1.18087573 | 0.00049874 |
| LINC01508 | 0.00380811 | 0.18876314 | 0.03744491 | 1.20775484 | 1.12229137 | 1.29972643 | 4.63E-07 |
| FARP1-AS1 | 0.01686514 | 0.18490171 | 0.07715746 | 1.20310018 | 1.03424932 | 1.39951753 | 0.01655609 |
| FAM225A | 0.0363948 | 0.17962011 | 0.05512138 | 1.19676264 | 1.07420858 | 1.33329861 | 0.00111952 |
| DIRC3 | 0.02560858 | 0.14878363 | 0.05676099 | 1.16042188 | 1.03824744 | 1.29697304 | 0.0087613 |
| PIK3CD-AS2 | 0.02870747 | 0.17119425 | 0.04410317 | 1.18672125 | 1.08844888 | 1.2938663 | 0.00010374 |
| NEBL-AS1 | 0.03048363 | 0.16732215 | 0.05892984 | 1.18213503 | 1.05318803 | 1.32686964 | 0.00452063 |
| TGFB2-AS1 | 0.04882559 | 0.15671893 | 0.05150298 | 1.16966681 | 1.05735969 | 1.29390258 | 0.00234302 |
| LINC00665 | 0.04669309 | 0.10084882 | 0.0414378 | 1.10610941 | 1.0198262 | 1.19969268 | 0.01494374 |
| SNHG7 | 0.00416529 | 0.23111381 | 0.08403338 | 1.26000264 | 1.06866629 | 1.48559627 | 0.00595476 |
| AC002511.2 | 0.00771861 | 0.21553573 | 0.05443413 | 1.2405263 | 1.11499151 | 1.38019481 | 7.51E-05 |
| ZNF529-AS1 | 0.0011937 | 0.33212298 | 0.09541463 | 1.39392427 | 1.15617107 | 1.68056865 | 0.00049985 |
| LINC00460 | 0.02193185 | 0.14751486 | 0.05375453 | 1.15895051 | 1.04305918 | 1.2877182 | 0.00606528 |
| LINC01136 | 0.00193132 | 0.20516646 | 0.054089 | 1.22772942 | 1.10423631 | 1.36503348 | 0.00014876 |
| DEPDC1-AS1 | 0.0040356 | 0.31618258 | 0.07420241 | 1.37188071 | 1.18619242 | 1.58663693 | 2.03E-05 |
| MAPKAPK5-AS1 | 1.59E-06 | 0.44071471 | 0.10758686 | 1.55381735 | 1.25840914 | 1.91857186 | 4.20E-05 |
| FOXP4-AS1 | 0.01951626 | 0.15490854 | 0.05026917 | 1.16755117 | 1.05800258 | 1.28844273 | 0.00205904 |
| SNHG20 | 0.0037809 | 0.28123283 | 0.09558736 | 1.32476201 | 1.09843346 | 1.5977248 | 0.00325937 |
| LINC00601 | 0.03057908 | 0.13369805 | 0.05180496 | 1.14304763 | 1.03268499 | 1.26520468 | 0.00985727 |
| LINC00941 | 0.03443936 | 0.1015832 | 0.04451802 | 1.10692201 | 1.01443264 | 1.20784396 | 0.02249853 |
| KDM4A-AS1 | 0.00420335 | 0.36370778 | 0.08977724 | 1.43865375 | 1.20652897 | 1.71543713 | 5.10E-05 |
| AC006369.2 | 0.00039655 | -0.2211394 | 0.06789933 | 0.80160496 | 0.70172085 | 0.91570673 | 0.00112648 |
| LINC01361 | 0.02185985 | 0.21845777 | 0.07042193 | 1.24415648 | 1.0837566 | 1.42829612 | 0.00192135 |
| LINC01424 | 0.04996028 | 0.19517715 | 0.08059436 | 1.2155263 | 1.03791625 | 1.42352929 | 0.01544716 |
| MKLN1-AS | 0.00012527 | 0.35405849 | 0.07901198 | 1.42483853 | 1.22042342 | 1.66349219 | 7.43E-06 |
| NIFK-AS1 | 0.00346495 | 0.30902362 | 0.10358342 | 1.36209454 | 1.11182614 | 1.66869752 | 0.0028513 |
| ZEB1-AS1 | 0.00625262 | 0.36948068 | 0.09230705 | 1.44698297 | 1.20751216 | 1.73394504 | 6.26E-05 |
| FOXD2-AS1 | 0.00053291 | 0.28007325 | 0.06434104 | 1.32322674 | 1.16645255 | 1.50107177 | 1.34E-05 |
| DGUOK-AS1 | 0.00182864 | 0.23276089 | 0.07889948 | 1.26207967 | 1.08125321 | 1.47314717 | 0.00317677 |
| LINC00426 | 0.03873451 | -0.1990659 | 0.07200673 | 0.81949587 | 0.71163045 | 0.94371099 | 0.00570021 |
| MYLK-AS1 | 0.00036444 | 0.29113887 | 0.07780729 | 1.33795037 | 1.14870977 | 1.55836681 | 0.00018271 |
| CDKN2B-AS1 | 0.0028394 | 0.17240958 | 0.0567379 | 1.18816438 | 1.06311719 | 1.32792001 | 0.002376 |
| NDUFB2-AS1 | 0.00144468 | 0.43198539 | 0.1180128 | 1.54031261 | 1.22223914 | 1.94116097 | 0.00025173 |
| ARHGAP31-AS1 | 1.34E-05 | 0.26078247 | 0.06093452 | 1.29794529 | 1.15183119 | 1.46259452 | 1.87E-05 |
| PRR34-AS1 | 0.04665299 | 0.1355277 | 0.06488832 | 1.14514092 | 1.00838388 | 1.30044496 | 0.03674104 |
| SNHG3 | 0.00108578 | 0.35343072 | 0.07446026 | 1.42394434 | 1.23058702 | 1.64768314 | 2.07E-06 |
| LINC01011 | 0.0018964 | 0.3244434 | 0.08608537 | 1.3832605 | 1.168498 | 1.63749499 | 0.000164 |
| LINC00847 | 0.00877937 | 0.25089353 | 0.10857593 | 1.28517325 | 1.03882329 | 1.58994345 | 0.0208458 |
| SAP30L-AS1 | 0.00100148 | 0.24895268 | 0.09309784 | 1.28268133 | 1.06874415 | 1.53944364 | 0.00749307 |
| DACT3-AS1 | 0.04582518 | 0.19770236 | 0.09185659 | 1.21859963 | 1.01782374 | 1.45898058 | 0.03137423 |
| DDX11-AS1 | 0.01004008 | 0.35355262 | 0.07783906 | 1.42411792 | 1.22261361 | 1.65883303 | 5.57E-06 |
| SNHG6 | 0.00082791 | 0.21376345 | 0.07476857 | 1.2383297 | 1.06953048 | 1.43376974 | 0.00424977 |
| SBF2-AS1 | 0.00168334 | 0.33337197 | 0.08738037 | 1.39566635 | 1.1759891 | 1.65637976 | 0.00013608 |
| BAALC-AS1 | 0.00552706 | 0.12520245 | 0.05642723 | 1.13337788 | 1.01471431 | 1.26591831 | 0.02649827 |
| MIR210HG | 9.23E-05 | 0.26271003 | 0.05308663 | 1.30044957 | 1.17194194 | 1.44304853 | 7.47E-07 |
| LRP4-AS1 | 0.02224512 | 0.23707179 | 0.07173905 | 1.26753211 | 1.10127197 | 1.45889272 | 0.00095098 |
| UBA6-AS1 | 0.00921 | 0.28958658 | 0.11963344 | 1.3358751 | 1.05665614 | 1.68887702 | 0.01549425 |
| TRIM52-AS1 | 0.02524563 | 0.30163362 | 0.11263762 | 1.35206577 | 1.08422772 | 1.68606817 | 0.00740827 |
| LUCAT1 | 0.0138227 | 0.11451445 | 0.03433002 | 1.12132884 | 1.0483619 | 1.19937435 | 0.00085087 |
| NPHP3-AS1 | 0.02679227 | 0.2080385 | 0.08900055 | 1.23126057 | 1.0341715 | 1.46591025 | 0.01941337 |
| LINC01060 | 0.00178681 | 0.2925788 | 0.05456578 | 1.33987832 | 1.20397893 | 1.49111739 | 8.23E-08 |
| LINC00942 | 0.01918557 | 0.09916391 | 0.02640326 | 1.10424728 | 1.04855652 | 1.16289589 | 0.00017283 |
| OSMR-AS1 | 0.00215675 | 0.16883723 | 0.06222557 | 1.18392741 | 1.04799347 | 1.33749318 | 0.00666149 |
| LINC01511 | 0.00040081 | 0.13880492 | 0.04909229 | 1.14889995 | 1.04350559 | 1.26493918 | 0.00469235 |
| ZFPM2-AS1 | 0.00055458 | 0.13385646 | 0.03220129 | 1.14322871 | 1.07330546 | 1.21770729 | 3.23E-05 |
| LINC01094 | 0.00919998 | 0.21171769 | 0.05671911 | 1.23579896 | 1.10577924 | 1.38110666 | 0.00018941 |
| OTUD6B-AS1 | 0.02193961 | 0.28818462 | 0.11329296 | 1.33400357 | 1.0683704 | 1.66568216 | 0.01096816 |
| WAC-AS1 | 2.43E-05 | 0.48793902 | 0.11649564 | 1.62895552 | 1.29642666 | 2.04677684 | 2.81E-05 |
| MIR100HG | 0.00014694 | -0.1490467 | 0.04877699 | 0.86152887 | 0.78298021 | 0.94795755 | 0.00224553 |
| SNHG1 | 0.02447894 | 0.33175884 | 0.09447935 | 1.39341677 | 1.15787071 | 1.67688006 | 0.00044568 |
| PXN-AS1 | 0.0059725 | 0.34108681 | 0.11075494 | 1.40647533 | 1.13202844 | 1.7474586 | 0.00207242 |
| EGLN3-AS1 | 2.37E-05 | 0.24473475 | 0.05796175 | 1.27728247 | 1.14011804 | 1.43094876 | 2.42E-05 |
| LINC00519 | 0.00535483 | 0.14724999 | 0.06233607 | 1.15864357 | 1.02539052 | 1.30921332 | 0.01816706 |
| LINC00648 | 0.01712899 | 0.06773812 | 0.03061509 | 1.07008504 | 1.00776362 | 1.1362605 | 0.02692712 |
| ATP2A1-AS1 | 0.01663332 | 0.15139257 | 0.05758565 | 1.1634533 | 1.03927855 | 1.30246466 | 0.00856368 |
| FBXL19-AS1 | 0.04518438 | 0.13329222 | 0.06435658 | 1.14258384 | 1.0071813 | 1.2961895 | 0.03834466 |
| LINC01311 | 0.02776488 | 0.22642176 | 0.08206251 | 1.25410448 | 1.0677805 | 1.47294136 | 0.00579541 |
| LINC01572 | 0.04074806 | 0.14794778 | 0.06019911 | 1.15945235 | 1.030413 | 1.3046514 | 0.01398531 |
| AC140912.1 | 0.00471552 | 0.13085156 | 0.06155845 | 1.13979858 | 1.0102514 | 1.28595792 | 0.03353263 |
| AC006538.1 | 0.00786732 | 0.14135155 | 0.05847171 | 1.1518295 | 1.02711006 | 1.29169331 | 0.01563034 |
| LINC00662 | 0.01483588 | 0.28383029 | 0.10537419 | 1.32820751 | 1.08036688 | 1.6329038 | 0.00706962 |
| LINC00909 | 0.03517172 | 0.25395972 | 0.10916456 | 1.28911988 | 1.04081193 | 1.59666701 | 0.01999757 |
| L3MBTL4-AS1 | 0.02778347 | 0.17688524 | 0.07817559 | 1.19349412 | 1.02394611 | 1.39111639 | 0.0236563 |
| RUNDC3A-AS1 | 0.04617614 | 0.16768697 | 0.06187646 | 1.18256637 | 1.0475052 | 1.3350418 | 0.00672774 |
| ZNF649-AS1 | 0.01158867 | 0.19981544 | 0.08737641 | 1.22117736 | 1.02897259 | 1.44928461 | 0.02220545 |
| LINC01224 | 0.04270424 | 0.12749983 | 0.03416475 | 1.13598467 | 1.06240812 | 1.21465673 | 0.00019003 |
| HMGN3-AS1 | 0.01356133 | 0.32243266 | 0.08266218 | 1.38048193 | 1.17400124 | 1.62327798 | 9.60E-05 |
| TMCC1-AS1 | 0.00013988 | 0.4402378 | 0.07362161 | 1.55307649 | 1.3443923 | 1.79415382 | 2.23E-09 |
| CASC15 | 0.00016032 | 0.15379522 | 0.04455274 | 1.16625204 | 1.0687326 | 1.27266991 | 0.00055649 |
| LINC01297 | 0.00031333 | 0.19292499 | 0.04990992 | 1.21279182 | 1.0997725 | 1.33742569 | 0.00011088 |
| PGM5P3-AS1 | 0.01578512 | -0.219277 | 0.09746614 | 0.80309926 | 0.66344649 | 0.97214837 | 0.02446318 |
| BACE1-AS | 0.00085806 | 0.36885807 | 0.10792453 | 1.44608235 | 1.17038163 | 1.78672844 | 0.00063143 |
| AC012065.1 | 4.41E-08 | 0.51503823 | 0.09526411 | 1.67370249 | 1.38863884 | 2.0172848 | 6.43E-08 |
| AL031587.1 | 0.00308541 | 0.28570034 | 0.0825319 | 1.33069364 | 1.13194884 | 1.56433356 | 0.00053678 |
| LINC00294 | 0.00318559 | 0.26910636 | 0.10937163 | 1.30879433 | 1.05626796 | 1.62169323 | 0.01387525 |
